# *Trdn-as* directs m6A-dependent transcriptional termination for accurate triadin isoform switching, preventing aberrant dyads and cardiomyopathy

**DOI:** 10.1038/s41467-026-75985-8

**Published:** 2026-07-25

**Authors:** Theresa Hofmann, Sara Hettrich, Bio Maria Ghéo Idrissou, Christian Waechter, Maria Weiss, Salma Hachim, Silke Kreher, Maximilian Staps, Laia Cañes Esteve, Sabine Pankuweit, Hendrik Milting, Mario Looso, Isabelle Marty, Stefan Engelhardt, Thomas Braun, Thomas Boettger

**Affiliations:** 1 Department of Cardiac Development and Remodelling, Max-Planck-Institute for Heart and Lung Research, Bad Nauheim, Germany; 2 Member of the German Centre for Cardiovascular Research (DZHK), Partner site Rhein-Main, Frankfurt am Main, Germany; 3 Institute of Pharmacology and Toxicology, School of Medicine, Technical University of Munich, Munich, Germany; 4 Member of the German Centre for Cardiovascular Research (DZHK), Partner site Munich Heart Alliance, Munich, Germany; 5 Department of Cardiology, Philipps University Marburg, Marburg, Germany; 6 Heart- and Diabetescenter NRW, Klessmann-Institute for Cardiovascular Research and Development, Ruhr-University Bochum, Bad Oeynhausen, Germany; 7 Bioinformatics Core Unit, Max Planck Institute for Heart and Lung Research, Bad Nauheim, Germany; 8 Grenoble Institut Neurosciences, University Grenoble Alpes, Inserm, U1216, CHU Grenoble Alpes, Grenoble, France; 9 Present Address: Department of Cardiology III, University Hospital Muenster, Albert-Schweitzer-Campus 1, Münster, Germany; 10 Present Address: Department of Internal Medicine, Universities of Giessen and Marburg Lung Center (UGMLC), Justus-Liebig University, Giessen, Germany

**Keywords:** Calcium signalling, Heart failure, Long non-coding RNAs, Cardiac hypertrophy

## Abstract

Heart failure is a leading cause of mortality, and impaired cardiac excitation-contraction coupling represents a potentially fatal trigger for myocardial dysfunction. Long non-coding RNAs (lncRNAs) can contribute to cardiomyopathy, but comprehensive mechanistic insights remain elusive. We demonstrate that reduction of the lncRNA *TRDN-AS* in human cardiomyopathy or abrogating it in human iPSC-derived cardiomyocytes and mice causes a switch of cardiac TRDN/TRISK32 to skeletal muscle TRDN/TRISK95. Transcription of *Trdn-as* in *cis* is essential for stalling RNA Pol II at the 3’ end of the cardiac *Trdn* transcript, promoting the formation of the cardiac TRDN/TRISK32 isoform. The m6A-methyltransferase METTL3 is crucial for RNA Pol II stalling, enforcing transcriptional termination and proximal polyadenylation of the *Trdn* transcript. Here, we establish that the switch of TRDN isoforms results in a significantly altered interactome of the cardiac calcium release complex, aberrant calcium handling, altered dyad structure, QT prolongation, and dilated cardiomyopathy in mice and humans.

## Abstract (web summary)

The lncRNA *Trdn-as* regulates the formation of the cardiac-specific TRDN isoform through transcriptional interference and m6A modification. This is crucial for accurate cardiac dyad calcium release and prevents QT prolongation and cardiomyopathy.

## Introduction

The coupling of membrane depolarization to the release of calcium from intracellular stores and subsequent activation of the contractile apparatus is indispensable for heart function. Perturbation of calcium handling leads to heart failure or arrhythmias such as catecholaminergic tachycardias. Furthermore, aberrant calcium handling worsens the course of different cardiac disorders, e.g., ischemic heart disease and dilated cardiomyopathy.

The principal mechanism of excitation-contraction (E-C) coupling is similar in heart and skeletal muscle cells, but important differences exist. The triad of skeletal muscle comprises two cisternae of the sarcoplasmic reticulum (SR) and one T-tubule. The T-tubule contains high concentrations of dihydropyridine receptors (DHPR), L-type voltage-gated Ca^2+^ channels, acting as voltage sensors. In cardiac muscle, a dyad forms by the close proximity of the SR with one T-tubule at the Z-disc level. In the triads of skeletal muscle cells, membrane depolarization opens the voltage-gated Ca^2+^-channel DHPR (CACNA1S), which in turn activates the intracellular Ca^2+^-channel RYR1, leading to release of Ca^2+^ stored in the SR, a mechanism known as voltage-induced calcium release (VICR)^1,2^. In dyads of cardiomyocytes, depolarization opens the cardiac DHPR (CACNA1C) and the resulting Ca^2+^ influx stimulates release of Ca^2+^ from the SR primarily via RYR2, a process known as calcium-induced calcium release (CICR)^3,4^. It is important to realize that the RyRs do not exist alone in the SR membrane, but form a complex with triadin, junctin, and calsequestrin (CSQ).

Triadin is a crucial component of the calcium-release complex (CRC). Together with CSQ and junctin, it is necessary for RyR channels to open and respond to luminal Ca^2+^. Inactivation of triadin in skeletal muscle causes muscle weakness, whereas inactivation in the heart impairs DHPR - RYR coupling and reduces Ca^2+^ release from the SR, promoting isoproterenol-induced ventricular tachycardia^5,6^. TRDN loss-of-function mutation in humans causes catecholaminergic polymorphic ventricular tachycardia (CPVT) associated with muscle weakness^7^ as well as long QT syndrome (LQTS) with exercise-induced cardiac arrest^8,9^. Several components of the calcium-release complex are encoded by paralogous genes, generating specific isoforms, either expressed in skeletal muscles or in the heart. In contrast, the skeletal muscle and cardiac-specific isoforms of TRDN are generated from a single gene by alternative splicing and polyadenylation. In skeletal muscle cells, transcripts are produced from the *Trdn* locus coding for a protein of 51 kDa (TRISK51) and 95 kDa (TRISK95), whereas cardiomyocytes produce a transcript coding for a protein of 32 kDa (TRISK32). TRISK51/95 and TRISK32 are identical in their N-terminal part, but TRISK32 lacks the C-terminal part present in TRISK51/95 and has a specific short C-terminal end. Both proteins interact with RyR channels and calsequestrin essential for calcium release from the SR, but the long C-terminal domain of the skeletal muscle isoform TRISK51 and TRISK95 fulfills additional, skeletal muscle-specific functions^10^. Stimulating expression of the non-coding *Trdn-as* transcript, transcribed in the opposite direction of the coding transcript, has been described to reduce the abundance of a skeletal muscle *Trdn* transcript in HL-1 cardiomyocytes^11^, whereas genomic deletion of exons of *Trdn-as* attenuates generation of the cardiac TRDN isoform in the heart^12^. Thus, at present, the mechanism of *Trdn-as* function and the relevance of *TRDN-as*-mediated suppression of TRISK51/95 for cardiac function in physiological and pathological conditions is unclear.

Long non-coding RNAs (lncRNAs) such as *Trdn-as* fulfill numerous different functions by distinctive molecular mechanisms, either acting in *cis* within the same gene locus in which they are transcribed, or in *trans* at other sites within the chromatin or at other cellular locations^13^. One example of a *trans*-acting lncRNA is *mitolnc*, which acts as an allosteric activator of BCKDH, controlling branched-chain amino acid metabolism in mitochondria of cardiomyocytes^14^. *Cis*-acting lncRNAs may activate or repress gene transcription^15^. Alternatively, *cis*-acting lncRNAs may regulate the formation of specific mRNA transcripts by various molecular mechanisms, determining the generation of distinct protein isoforms as proposed for *Trdn-as*^11,12^.

In this work, we investigated the role and function of *Trdn-as-*dependent triadin isoform switching for regulating cardiac excitation-contraction coupling and dilated cardiomyopathy in mouse and human hearts. We uncovered that reduced *Trdn-as* expression is associated with defective triadin isoform switching in human cardiomyopathy, leading to accumulation of skeletal muscle TRISK51/95, in particular in patients with documented arrhythmia. Loss of *Trdn-as* impairs Ca^2+^ handling in human iPSC-derived cardiomyocytes. Analysis of *Trdn-as* deficient mice unveiled structural reconfiguration of the cardiac dyads, massive changes in CICR, and aberrant spontaneous calcium oscillations, leading to QT prolongation, bradyarrhythmia, dilated cardiomyopathy, and premature lethality. Furthermore, we demonstrate that termination of *Trdn* transcription, required for triadin isoform switching, depends on the m6A writer Mettl3.

## Results

### *TRDN-AS* regulates E-C coupling in human cardiomyopathy

**Fig. 1:**
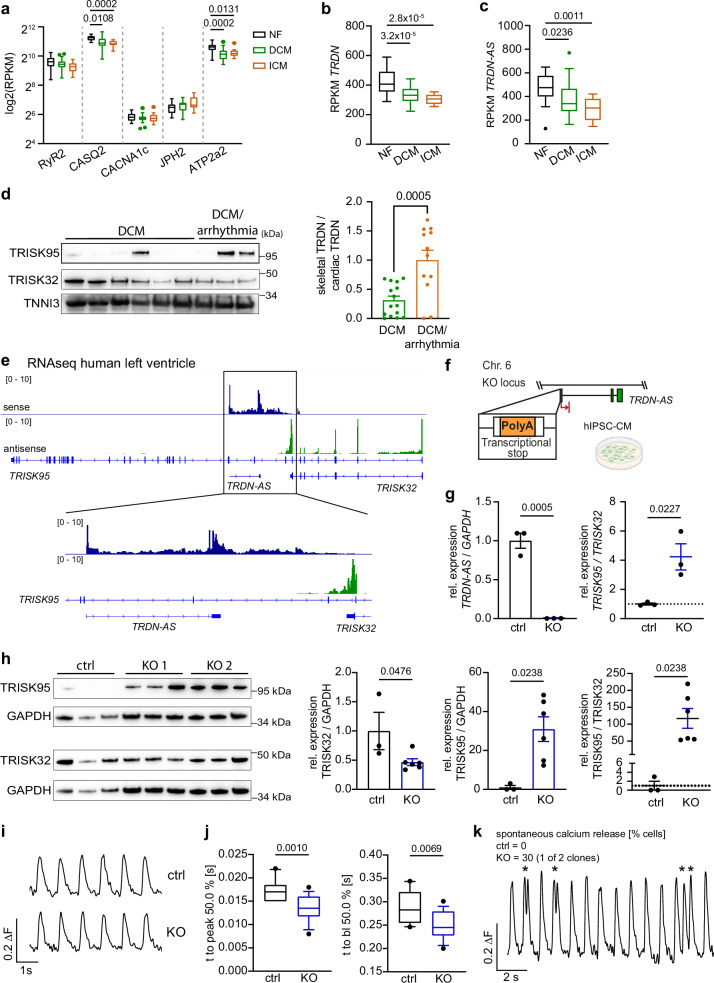
*TRDN-AS* controls expression of TRDN isoforms and depolarization-induced calcium release. **a–c** Expression of genes involved in CICR in human heart samples (non-failing hearts (NF) = 14, dilated cardiomyopathy hearts (DCM) = 37, ischemic cardiomyopathy hearts (ICM) = 13; data from GSE116250; two-tailed multiple Mann-Whitney test, Holm-Šidák correction, Tukey boxplot) and **b** expression of *TRDN* and of **c** *TRDN-AS* detected in the dataset (NF = 14, DCM = 37, ICM = 13; one-way ANOVA using Fisher’s LSD test, Tukey boxplots). **d** Western blot and quantification of TRDN isoforms TRISK95 and TRISK32 in heart samples from patients with DCM or DCM with ventricular arrhythmia. Additional samples (Supplementary Fig. 1) are included in the statistical analysis (*n* = 15 DCM, *n* = 12 DCM-arrhythmia patient samples; TRISK95/32 ratios were normalized to the DCM/arrhythmia samples of the respective blots; two-tailed unpaired Student’s t-test, mean ± SEM). **e** Genome browser view of *TRDN* and *TRDN-AS* expression in human left ventricles using stranded RNA-seq data (ENCODE, #ENCSR000AHH). **f** Disruption of *TRDN-AS* transcription by insertion of a polyA sequence into the first exon in human induced pluripotent stem cells (hiPSC). Clones were differentiated into hiPSC-derived cardiomyocytes (hiPSC-CMs). Created in BioRender. Gerhardt, T. (2026) https://BioRender.com/q9swlm0. **g** RT-qPCR analysis of *TRDN-AS* and of the skeletal muscle *TRDN*/cardiac *TRDN* isoform ratio in hiPSC-CMs (*n* = 3 ctrl clones, *n* = 3 *TRDN-AS* KO clones; two-tailed unpaired Student’s *t* test, mean ± SEM). **h** Western blot and quantification of TRDN isoforms in hiPSC-CMs detects TRISK95 and TRISK32 upon loss of *TRDN-AS* expression (*n* = 3 replicates of ctrl clone, *n* = 3 replicates for each *TRDN-AS* KO clone, two-tailed Mann-Whitney test, mean ± SEM). **i, j** IonOptix calcium measurements using control and *TRDN-AS* KO hiPSC-CMs for calcium release (time to 50.0% peak) and reuptake (time to 50.0% baseline); (*n* = 10 control and *n* = 18 *TRDN-AS* KO hiPSC-CMs, two-tailed unpaired Student's *t* test, Box: 25th–75th percentiles, whiskers: 10th–90th percentile, center: median). **k** Representative IonOptix trace illustrating spontaneous calcium-releases (*) in *TRDN-AS* KO hiPSC-CMs. 30% of *TRDN-AS* KO hiPSC-CMs exhibited spontaneous calcium-release within 1 Hz stimulation.

Heart failure and left ventricular dysfunction often lead to arrhythmias, but vice versa, tachycardias, atrial fibrillation, and premature ventricular contractions can induce reversible dilated cardiomyopathy (DCM)^16^. To explore the underlying molecular processes, we analyzed the expression of genes coding for components of the excitation-contraction coupling machinery using a published RNA-seq dataset from left ventricles of failing human hearts (GSE116250)^17^. We detected reduced expression of *TRDN*, *CASQ2*, and *ATP2A2*, but not of *CACNA1C*, *RYR2*, and *JPH2* (Fig. 1a, b). Next, we asked whether changes in the abundance of these coding transcripts may correlate with changes in the abundance of long non-coding RNAs. Interestingly, we observed reduced expression of the long non-coding RNA *TRDN-AS* (Fig. 1c), which is transcribed in an antisense direction relative to the protein-encoding human *TRDN* gene (Fig. 1e). Since *TRDN-AS* has been described to play a role in regulating the cardiac-specific isoform of TRDN^11,12^, we investigated potential changes in TRDN isoform abundance in human DCM samples with and without arrhythmias. Western blot analysis revealed the appearance of the skeletal muscle-specific TRDN (TRISK95) isoform in DCM samples, indicating that reduced expression of *TRDN-AS* can result in aberrant TRISK95 expression in parts of the myocardium of DCM patients. Moreover, in DCM patients with a record of myocardial arrhythmia, we observed an increased ratio of TRISK95/TRISK32 (Supplementary Figs. S1 and 1d).

To investigate whether reduced expression of *TRDN-AS* is critical for the presence of TRISK95 in cardiomyocytes and the advent of arrhythmias, we manipulated human iPSCs by insertion of a polyA-signal sequence into the first exon of *TRDN-AS* (Fig. 1e, f). Insertion of the polyA-site prevented expression of *TRDN-AS* after differentiation of hiPSCs towards the cardiomyocyte lineage, whereas expression of *TRDN* and *TRDN-AS* was readily detectable in unmodified hiPSC-derived cardiomyocytes. RT-qPCR analysis using isoform-specific oligonucleotide pairs revealed an increased ratio of skeletal to cardiac muscle *TRDN*-mRNA expression in mutated versus control cells (Fig. 1g and Supplementary Fig. S2a, b). These findings were even more pronounced at the protein level. Western blot analysis showed a strong raise of TRISK95 and minor decline of TRISK32, resulting in a massive increase of the skeletal/cardiac isoform ratio (Fig. 1h). To examine functional consequences of the isoform shift, we monitored depolarization-induced calcium release using the calcium sensitive dye FURA-2-AM and IonOptix system (Fig. 1i). *TRDN-AS*-depleted hiPSC-derived cardiomyocytes showed a much faster release and reuptake of calcium compared to control cells, indicated by reduced time to 50% peak and reduced time to 50% baseline (Fig. 1j). Moreover, we also observed spontaneous calcium release events in mutated but not in control hiPSC-derived cardiomyocytes, indicating a critical role of *TRDN-AS* for precise excitation-contraction coupling (Fig. 1k).

### *Trdn-as* controls the release of calcium from the SR and prevents DCM

**Fig. 2:**
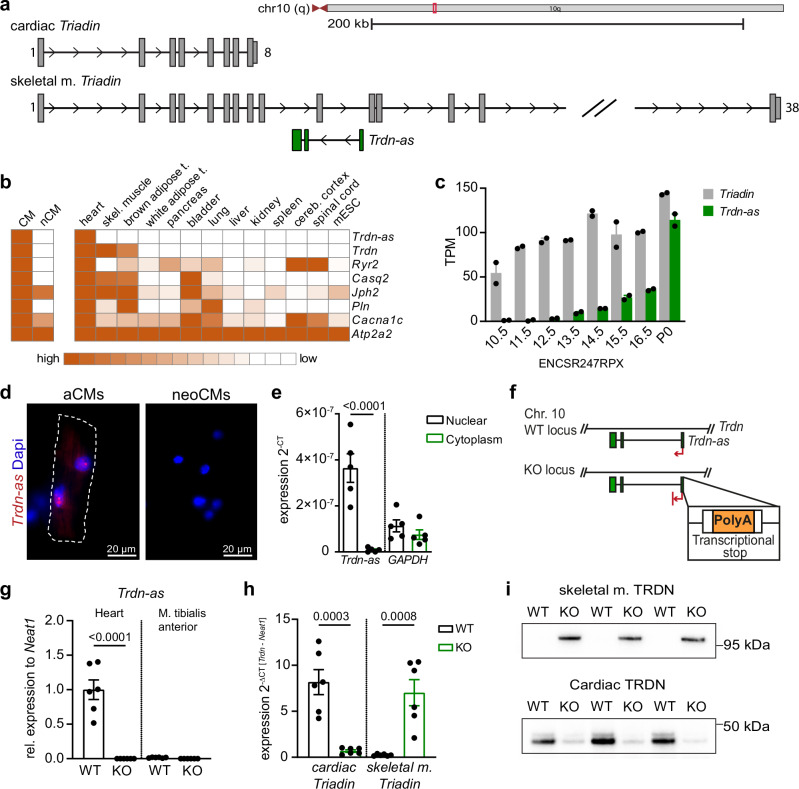
*Trdn-as* is a cardiac-specific nuclear non-coding transcript regulating triadin isoform expression. **a** Genomic localization of the protein-coding gene *triadin* and its antisense long non-coding RNA *Trdn-as* (D830005E20Rik). The two main *triadin* isoforms are depicted. The heart-specific isoform cardiac *triadin* (ENSMUST00000219982.2) and the skeletal muscle-specific *triadin* isoform (ENSMUST00000095762.5) are derived by alternative splicing. Both isoforms have unique 3’ untranslated regions (smaller bar). *Trdn* exons are depicted in grey and *Trdn-as* in green. Arrows indicate direction of transcription. **b** Microarray-based expression analysis of *Trdn-as* and additional genes involved in calcium release in cardiomyocytes across multiple mouse tissues, in isolated adult cardiomyocytes (aCMs), and non-cardiomyocytes (nCMs). **c** Expression of *triadin* (grey) and *Trdn-as* (green) during cardiac development based on the ENCODE RNA-seq dataset #ENCSR247RPX (n = 2 biological replicates/stage, mean ± SEM). **d** In-situ hybridization (RNA-FISH) detects localization of *Trdn-as* in the nucleus of adult (aCMs) but not in neonatal cardiomyocytes (neoCMs). Dotted line indicates outline of a CM. **e** RT-qPCR analysis of nuclear and cytoplasmic RNAs in adult mouse hearts reveals subcellular distribution of *Trdn-as* compared to *Gapdh* (*n* = 5, cytoplasmic and nuclear fractions, one-way ANOVA, Bonferroni’s correction, mean ± SEM). **f** Knock-out (KO) of *Trdn-as* by insertion of a premature transcription termination cassette by Cas9 genome editing. **g** RT-qPCR of *Trdn-as* in adult mouse hearts reveals loss of RNA expression in homozygous KO mice. RNA from WT and *Trdn-as* KO *tibialis anterior* muscle was used for comparison (*n* = 6 WT mice, *n* = 6 *Trdn-as* KO mice, two-tailed unpaired Student’s *t* test, mean ± SEM). **h** Distribution of the cardiac and skeletal *triadin* isoforms in WT and *Trdn-as* KO hearts was quantified by RT-qPCR using paired primers amplifying specific isoforms of *triadin* (*n* = 6 WT hearts, *n* = 6 *Trdn-as* KO hearts, two-tailed unpaired Student’s *t* test, mean ± SEM). **i** Western blot analysis of heart membrane lysates for specific TRDN isoforms demonstrates loss of cardiac TRDN and de novo expression of skeletal TRDN protein in KO mice (*n* = 3 WT hearts, *n* = 3 *Trdn-as* KO hearts).

In the mouse genome, the architecture of the *Trdn/Trdn-as* on chromosome 10 is similar to humans, placing the *Trdn-as* gene downstream of the last exon of the cardiac-specific *Trdn*-isoform within the *Trdn* gene with transcription in an antisense direction relative to the protein-coding *Trdn* gene (Fig. 2a). Expression of *Trdn-as* in mice and humans is confined to cardiomyocytes, whereas *Trdn* and different components of the calcium release complex, such as *Ryr2*, *Casq2*, and *Jph2* were found in multiple tissues (Fig. 2b and Supplementary Fig. S2c). Expression of *Trdn* during heart development starts at E10.5, whereas *Trdn-as* expression begins at E12.5 and reaches comparable levels to *Trdn* by P0 (Fig. 2c). RNA FISH and RT-qPCR using RNA from nuclear and cytoplasmic fractions of cardiomyocytes revealed a predominant nuclear localization of *Trdn-as* (Fig. 2d, e).

**Fig. 3:**
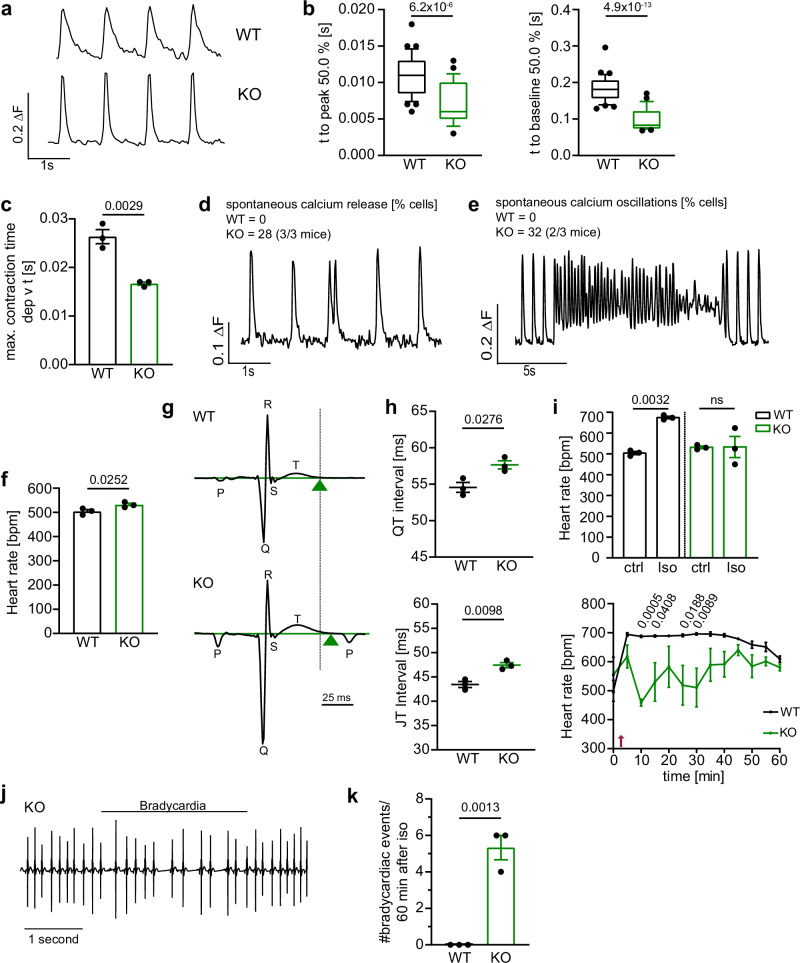
Loss of *Trdn-as* expression induces elevated calcium cycling and aberrant calcium release in adult cardiomyocytes (CMs). **a** Representative Ca^2+^ transients of isolated WT and *Trdn-as* KO adult mouse CMs under steady-state conditions (1 Hz field-stimulation). **b** Time to 50% peak and time to 50% baseline reflect the velocity of the increase and decrease of the intracellular calcium concentration, respectively (36 WT and 27 *Trdn-as* KO cells of 3 mice per group were recorded. Box: 25th–75th percentiles, whiskers: 10th–90th percentiles, center: median; two-tailed Mann–Whitney test). **c** Sarcomere contraction speed of *Trdn-as* KO CMs (*n* = 3 WT hearts, *n* = 3 *Trdn-as* KO hearts, two-tailed unpaired Student’s *t* test, mean ± SEM). **d** Spontaneous calcium release in *Trdn-as* KO cardiomyocytes. 28% of all *Trdn-as* KO CMs isolated from three *Trdn-as* KO mice exhibited extra peaks. **e** Spontaneous calcium oscillations in *Trdn-as* KO cardiomyocytes. Irregular calcium oscillations were observed in 32% of all *Trdn-as* KO CMs isolated from two of three *Trdn-as* KO mice. **f** Telemetric records of WT and *Trdn-as* KO mouse heart rates (bpm, beats per minute; steady state conditions, record 6.00 pm to 6.00 am; *n* = 3 WT mice, *n* = 3 *Trdn-as* KO mice, one-tailed unpaired Student’s *t* test, mean ± SEM). **g, h** Increased QT and JT times in ECGs (*n* = 3 WT mice, *n* = 3 *Trdn-as* KO mice, two-tailed unpaired Student’s *t* test, mean ± SEM). **i** Heart rate before and after injection of isoproterenol (Iso). The 30-min averaged heart rate (top) and the timeline (bottom) reveal a blunted response of *Trdn-as* KO mice to isoproterenol (*n* = 3 WT mice, *n* = 3 *Trdn-as* KO mice; averaged heart rate: one-way ANOVA using Fisher’s LSD test, timeline: two-way ANOVA, Šidák correction, mean ± SEM). **j, k** Representative ECG trace of persistent bradycardia observed in *Trdn-as* KO mice after isoproterenol injection. **k** Quantification of events of ventricular arrhythmia within 1 h after isoproterenol injection (*n* = 3 WT mice, *n* = 3 *Trdn-as* KO mice, two-tailed unpaired Student’s *t* test, mean ± SEM).

To expand our studies in hiPSC-derived cardiomyocytes and analyze the function of *Trdn-as* under physiological conditions in the mouse heart in vivo, we inserted a polyA cassette into the first exon of the *Trdn-as* genomic locus in mouse ES cells, which disrupts transcription of *Trdn-as* (Fig. 2f). Mutant ES cells were used to generate *Trdn-as*-deficient mouse lines. *Trdn-as*-deficient hearts showed a complete loss of *Trdn-as* expression based on RT-qPCR analysis, accompanied by loss of cardiac *Trdn* isoform expression and gain of skeletal muscle *Trdn* isoform expression. Western blot analysis confirmed the appearance of TRISK95 and massive reduction of TRISK32 in *Trdn-as*-deficient hearts, partially phenocopying the situation in human DCM patients (Fig. 2g–i). Also, we observed the expression of the skeletal muscle-specific TRISK51 isoform of TRDN (Supplementary Fig. S2e), which is derived by intron retention within the skeletal muscle-specific transcript. Similar to *Trdn-as*-deficient hiPSC-derived cardiomyocytes, we observed a faster release and subsequent faster reuptake of calcium in *Trdn-as*-deficient primary mouse cardiomyocytes, resulting in faster cardiomyocyte contraction (Fig. 3a–c). Moreover, we observed spontaneous calcium release events and spontaneous, self-terminating calcium oscillations (Fig. 3d, e). AAV-mediated ectopic expression of TRISK95 in WT cardiomyocytes similarly resulted in spontaneous calcium release events and calcium oscillations (Supplementary Fig. S3).

Telemetric ECG recordings revealed an increased heart rate at night and prolonged QT and JT intervals in 20-week-old *Trdn-as*-deficient mice under baseline conditions (Fig. 3f–h). Adrenergic stimulation by i.p. injection of isoproterenol increased the heart rate in WT animals, but not in *Trdn-as*-deficient animals (Fig. 3i). Remarkably *Trdn-as*-deficient mice developed non-sustained ventricular bradyarrhythmia after adrenergic stimulation (Fig. 3j, k).

**Fig. 4:**
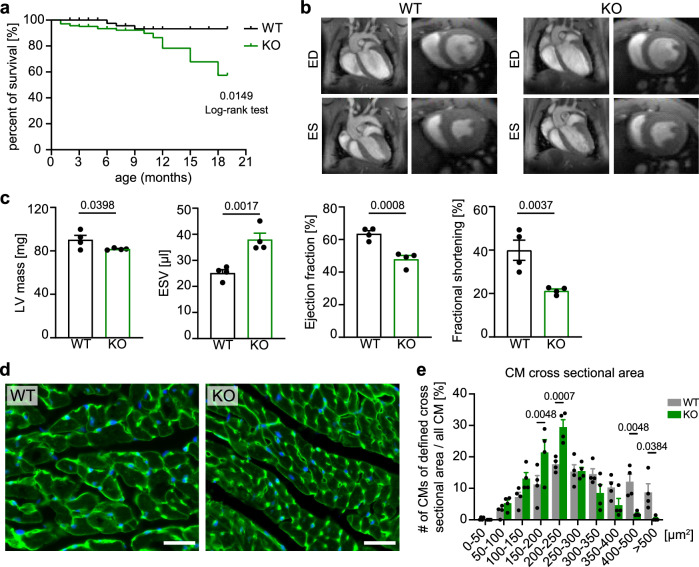
Expression of the skeletal muscle isoform of TRDN in the heart leads to dilated cardiomyopathy. **a** Survival rate of WT and *Trdn-as* KO mice until 21 months (165 WT and 207 *Trdn-as* KO mice, log-rank Mantel-Cox test). **b, c** Representative Magnetic Resonance Imaging (MRI) recordings of 40-week-old WT and *Trdn-as* KO mice in end-systole (ES) and end-diastole (ED). Left-panel images are long-axis four-chambered views and right-panel images present mid-level two-chambered short-axis views. Quantification for end-systolic volume (ESV), left ventricular (LV) mass, ejection fraction, and fractional shortening indicates a commencing dilated cardiomyopathy in KO mice (*n* = 4 WT, *n* = 4 *Trdn-as* KO mice, one-tailed unpaired Student’s *t* test, mean ± SEM). **d, e** Histological examination of left ventricular myocardium of 40-week-old WT and *Trdn-as* KO mice stained using WGA-Alexa488 to evaluate cardiomyocyte cross-sectional area. Numbers of cardiomyocytes with respective cross-sectional areas were quantified in relation to the overall number of cardiomyocytes (scale bars correspond to 25 µm, *n* = 4 WT hearts, *n* = 4 *Trdn-as* KO hearts, two-way ANOVA, Šidák correction, mean ± SEM).

Abrogation of *Trdn-as* transcription in mice reduced survival during ageing. We observed a reduction in survival beginning at 10 months, with 60% survival rate at 18 months of age (Fig. 4a). Although it is tempting to connect the reduced survival rate with the observed episodes of arrhythmia, proof for the actual cause of premature death is still missing. In addition, magnetic resonance imaging of ageing WT and *Trdn-as* KO mice showed signs of DCM in *Trdn-as*-deficient animals at 40 weeks of age (Fig. 4b), including moderately reduced heart weight/body weight ratios, increased end-systolic volume, reduced LV mass, reduced ejection fraction, and reduced fractional shortening (Fig. 4c and Supplementary Fig. S4a–d). No histological changes were observed in younger mice, however, histological quantifications of left-ventricle cross-sections in 40-week-old WT and *Trdn-as* KO mice confirmed reduced left ventricular mass and a decrease in CM cross-sectional area (Fig. 4d, e).

### Loss of *Trdn-as* expression changes the structure of the cardiac dyad

**Fig. 5:**
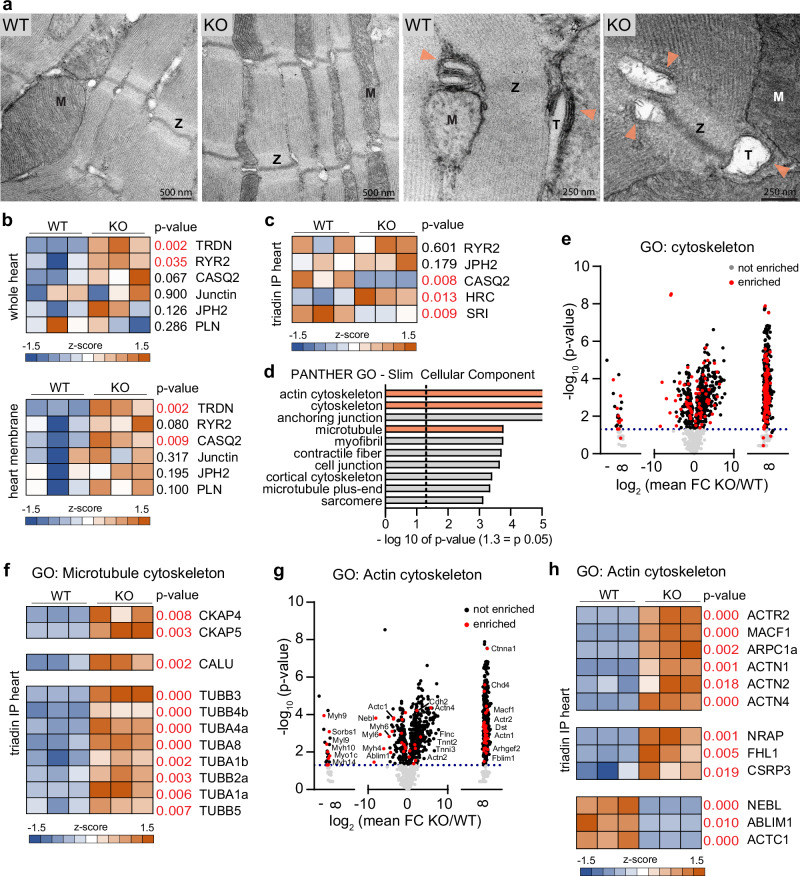
Loss of *Trdn-as* and subsequent generation of TRISK95 deforms cardiac dyads. **a** Transmission electron microscopy of WT and *Trdn-as* KO left ventricular myocardium reveals deformation of cardiac dyad structures (arrowheads: SR structures; M: mitochondria; T: T-tubule; Z: Z-disc). **b** Proteomics of whole-heart ventricular lysates and membrane fractions reveals increased abundance of CRC-related proteins (RYR2, CASQ2) in *Trdn-as* KO hearts (*n* = 3 WT hearts, *n* = 3 *Trdn-as* KO hearts, two-tailed unpaired Student’s t-test on LFQ intensities). **c** TRDN immunoprecipitation (IPs) of whole-heart lysates reveals altered interaction of TRDN isoforms with calcium-binding and regulatory proteins in KO hearts (*n* = 3 WT hearts, *n* = 3 *Trdn-as* KO hearts, two-tailed unpaired Student’s *t* test on LFQ intensities). **d** Gene ontology (GO) analysis of proteins significantly enriched in WT- or *Trdn-as* KO heart. Pan-TRDN-IPs using PANTHER indicates enrichment of TRDN-interacting proteins belonging to the cytoskeleton (*n* = 3 WT hearts, *n* = 3 *Trdn-as* KO hearts; https://www.pantherdb.org/; one-sided Fisher’s exact test, uncorrected *p*-values). **e, f** Volcano plot and heat map of proteins identified after TRDN-IP using WT- and *Trdn-as* KO heart lysates. Proteins labeled by the GO term “cytoskeleton” are marked red in the volcano plot. Proteins with FC -∞ or ∞ were exclusively detected in TRDN-IP of WT- or *Trdn-as* KO hearts, respectively. The heat map includes selected significantly enriched proteins annotated by the GO term “microtubule cytoskeleton” including the linker proteins CKAP4 and CKAP5 (*n* = 3 WT hearts, *n* = 3 *Trdn-as* KO hearts, two-tailed unpaired Student’s *t* test on LFQ intensities). **g, h** Volcano plot and heat map of proteins identified after TRDN-IP using WT- and *Trdn-as* KO heart lysates. Proteins labeled by the GO term “actin cytoskeleton” are marked in red in the volcano plot. The heat map includes selected significantly enriched proteins annotated by the GO term “actin cytoskeleton” illustrating the reconfiguration of TRDN-interactions, including reduced interaction with cardiac-specific proteins ABLIM1, ACTC1 and NEBL in *Trdn-as* KO hearts (*n* = 3 WT hearts, *n* = 3 *Trdn-as* KO hearts, two-tailed unpaired Student’s *t* test on LFQ intensities).

We reasoned that the appearance of TRISK51/95 and reduction of TRISK32 in cardiomyocytes of *Trdn-as*-deficient hearts may change the structure of the cardiac dyads, probably towards skeletal muscle triads, which facilitate direct coupling between the voltage-gated calcium channel and the skeletal muscle-specific RYR1 SR calcium release channel. This assumption was based on previous reports describing that TRISK51/95 organizes the triad and the CRC via protein-protein interactions mediated by its C-terminal part, which is absent in TRISK32^5,18,19^. In fact, recruitment of the microtubule/ER link CKAP4/CLIMP63 protein by TRISK51/95 but not TRISK32 is sufficient to restructure the ER membrane morphology in non-muscle cells^20^. Interestingly, we observed constriction of the dyad structure and the junction between plasmalemma and the SR in *Trdn-as*-deficient cardiomyocytes (Fig. 5a and Supplementary Fig. S4e–h). Analysis of the whole heart proteome revealed increased amounts of RYR2 in the heart and increased amounts of CASQ2 in respective membrane preparations (Fig. 5b). Furthermore, co-immunoprecipitation experiments revealed massive changes in the interactome of TRDN in mutant hearts. We detected a reconfiguration of the interaction with calcium-binding proteins located in the lumen of the SR that interact with RYR2 and thereby affect calcium release, whereas interactions with RYR2 and JPH2 were not altered. The interaction of TRDN with CASQ2 was reduced, and interaction with HRC was increased, which may enhance RYR2 activity^21^. In addition, we observed reduced interaction of TRDN with the calcium-binding protein SRI that is known to reduce spontaneous activity of the RYR2^22^ (Fig. 5c). Most likely, the altered interactions of TRDN with calcium-binding proteins are responsible for the increased dynamics of calcium release in *Trdn-as*-deficient cardiomyocytes. This conclusion is also supported by unbiased analysis of the TRDN-interactome in cardiomyocytes, which uncovered strong interactions of TRDN with the linker protein CKAP4, the related CKAP5, and components of the microtubular and actin cytoskeleton in *Trdn-as*-deficient hearts (Fig. 5d–h). Taken together, the newly arising interactions of TRISK95 with cytoskeletal components explain the constriction of the dyad in mutant cardiomyocytes, which together with the reduced interaction of the CRC with CASQ2 and SRI, allows faster calcium release from the SR. Faster calcium release in combination with reduced interaction of SRI may also permit faster reuptake of calcium.

### *Trdn-as* is controlled by a bidirectionally active promoter

**Fig. 6:**
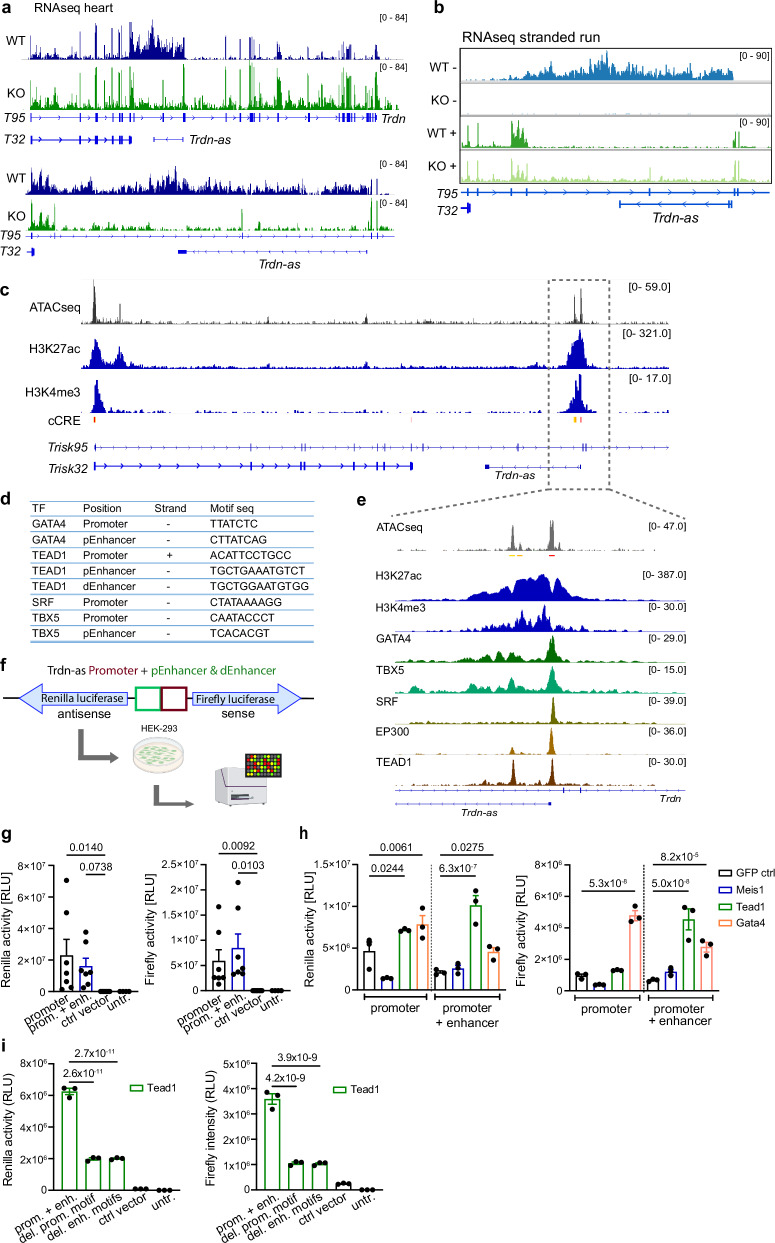
*Trdn-as* transcription is driven by a cardiac-specific, bidirectionally active promoter and extends to exon 8/9 of *Trdn.* **a** Genome browser view of the *triadin* (top) and *Trdn-as* locus (lower; including *Trdn* until exon 8 of cardiac *Trdn*, TRISK32-encoding transcript) visualizing RNA-seq data of WT and *Trdn-as* KO hearts. **b** Stranded RNA-seq of the *Trdn-as* and parts of the *Trdn* locus for WT and *Trdn-as* KO hearts (+sense reads, −antisense reads). *Trdn-as* transcription extends well beyond the last annotated exon of *Trdn-as*. *Trdn-as* KO heart RNA-seq reveals no antisense reads. **c** Accessible and active chromatin regions within the *Triadin* and *Trdn-as* locus revealed by available mouse heart ATAC-seq (#ENCSR451NAE), ChIP-seq (#GSM1264378, #ENCFF060VQA) data, and the ENCODE cCREs track (GRCm38/mm10, red cCRE mark: promoter elements, yellow: enhancer elements). cCRE elements align with chromatin accessibility and H3K27ac/H3K4me3 deposition. **d** Transcription factors (TF) potentially regulating *Trdn-as* expression, identified by TF binding analysis of the *Trdn-as* promoter (GRCm39: chr10:33132178-33132599), proximal (chr10:33130253-33130709) and distal enhancer (chr10:33130033-33130252) elements. **e** Available heart ChIP-seq data (#GSM2944730, #ENCSR777VNA, #GSM3067579, #GSM3067576, #GSM3518677) reveal TF binding with *Trdn-as* promoter and enhancer elements. GATA4, TBX5, and TEAD1 are observed at promoter and enhancer elements; SRF and EP300 are observed at the *Trdn-as* promoter element. **f** Assay used to investigate transcriptional regulation of *Trdn-as*. The identified promoter and the two enhancer elements of *Trdn-as* were inserted into a dual-Luciferase reporter and transfected into HEK-293 cells. Created in BioRender. Gerhardt, T. (2026) https://BioRender.com/2wrt5ii. **g** Luciferase assays reveal activity in antisense and sense direction (*n* = 7 transfections for promoter, promoter + enhancer and empty ctrl vector; untransfected cells *n* = 5; Kruskal–Wallis, Dunn’s multiple comparisons test, mean ± SEM). **h** Co-transfection of reporter vectors with vectors expressing GFP, MEIS1, GATA4 or TEAD1 (*n* = 3 transfections per group, one-way ANOVA, Fisher’s LSD test, mean ± SEM). **i** Co-transfection of the TEAD1 expression vector with reporter vectors containing promoter/enhancer sequences and reporter vectors after deletion of the TEAD1 binding motifs in promoter or enhancers, respectively (*n* = 3 transfections per group, one-way ANOVA, Fisher’s LSD test, mean ± SEM).

Since *Trdn-as* represses transcription of the longer, skeletal muscle-specific TRISK51/95 isoforms in cardiomyocytes, we explored the molecular features of the *Trdn* locus in more detail. RNA-seq of control hearts detected reads from *Trdn* exons coding for TRISK32 and, to a lesser extent, from *Trdn* exons coding for TRISK95. Furthermore, we detected reads originating from *Trdn-as*. Reads from *Trdn-as* were mostly absent in *Trdn-as*-deficient hearts, validating successful abrogation of *Trdn-as* transcription (Fig. 6a). Stranded RNA-seq analysis confirmed that *Trdn* is transcribed from the plus (s) strand and *Trdn-as* from the minus (as) strand. As expected, reads for *Trdn-as* from the minus (as) strand were completely absent in *Trdn-as* mutant cells (Fig. 6b). Transcription of *Trdn-as* extended well beyond the previously described last exon of *Trdn-as*, reaching exon 8 of *Trdn*. Deletion of *Trdn-as* expression in the heart increased transcription of *Trdn* downstream of *Trdn-as* (>Trdn exon 10), confirming the regulatory role of *Trdn-as* for suppressing transcription of exons coding for TRISK95 (Fig. 6a-b; Supplementary Fig. S5a). Surprisingly, we also recorded some transcription of TRISK95 exons in WT hearts (Fig. 6a-b), although TRISK51/95 protein was completely absent when *Trdn-as* is transcribed (Fig. 2i). Closer analysis revealed that the RNA reads did not belong to the cardiac *Trdn* transcript, but to a discrete transcript from the sense strand 3’ of the *Trdn-as* promoter, driven by the bidirectional *Trdn-as* promoter. This transcript starts with a previously unknown exon that is not found in the skeletal muscle *Trdn* transcript. The previously undetected 1st exon of the transcript is spliced to the remaining 3’ exons of skeletal muscle TRDN, generating an RNA that does not contain an open reading (Supplementary Fig. S5b, c).

Analysis of publicly available mouse heart datasets, including ATACseq, H3K27ac and H3K4me3 ChIP-seq data (ENCSR451NAE, GSM1264378, ENCFF060VQA, respectively) as well as the ENCODE-annotated cis-regulatory elements track (GRCm38/mm10) identified the promoter region of *Trdn-as* as well as two potential enhancer regions upstream of the first annotated exon of *Trdn-as* (Fig. 6c). Analysis of putative transcription factor binding sites and multiple ChIP-seq datasets revealed binding of GATA4, TBX5, TEAD1, EP300 and SRF to the regulatory region of *Trdn-as* (Fig. 6c–e) (GSM3067576, GSM3067579, GSM3518677, ENCSR777VNA, GSM2944730, respectively). To investigate whether the identified sequences are sufficient to drive expression of *Trdn-as*, we inserted them into a dual reporter vector, allowing detection of transcription upstream and downstream of the inserted element (Fig. 6f). Transfection of the vector with the inserted putative regulatory elements into HEK-293 cells induced bidirectional expression of the reporter genes, whereas no signals were obtained when using the control vector (Fig. 6g). Additional co-expression of GATA4 and TEAD1 increased expression of both the *Trdn-as* reporter and the sense *Trdn* 3’ reporter, whereas MEIS1 had no effects (Fig. 6h). Deletion of the TEAD1 binding sites in the enhancer or the promoter of the reporter vector reduced the TEAD1-induced activation of the reporter proteins, confirming the specificity of the TEAD1 activity (Fig. 6i). Interestingly, TEAD1 but not GATA4 and SRF are downregulated in human DCM and ICM samples, with significant correlation between *TEAD1* and *TRDN-AS* expression in NF and ICM samples, suggesting that the downregulation of *Trdn-as*, resulting in appearance of TRISK95 in such conditions, is -at least in part- due to lower TEAD1 levels (Supplementary Fig. S5d, e).

### TRDN isoform depends on transcriptional interference and m6A

**Fig. 7:**
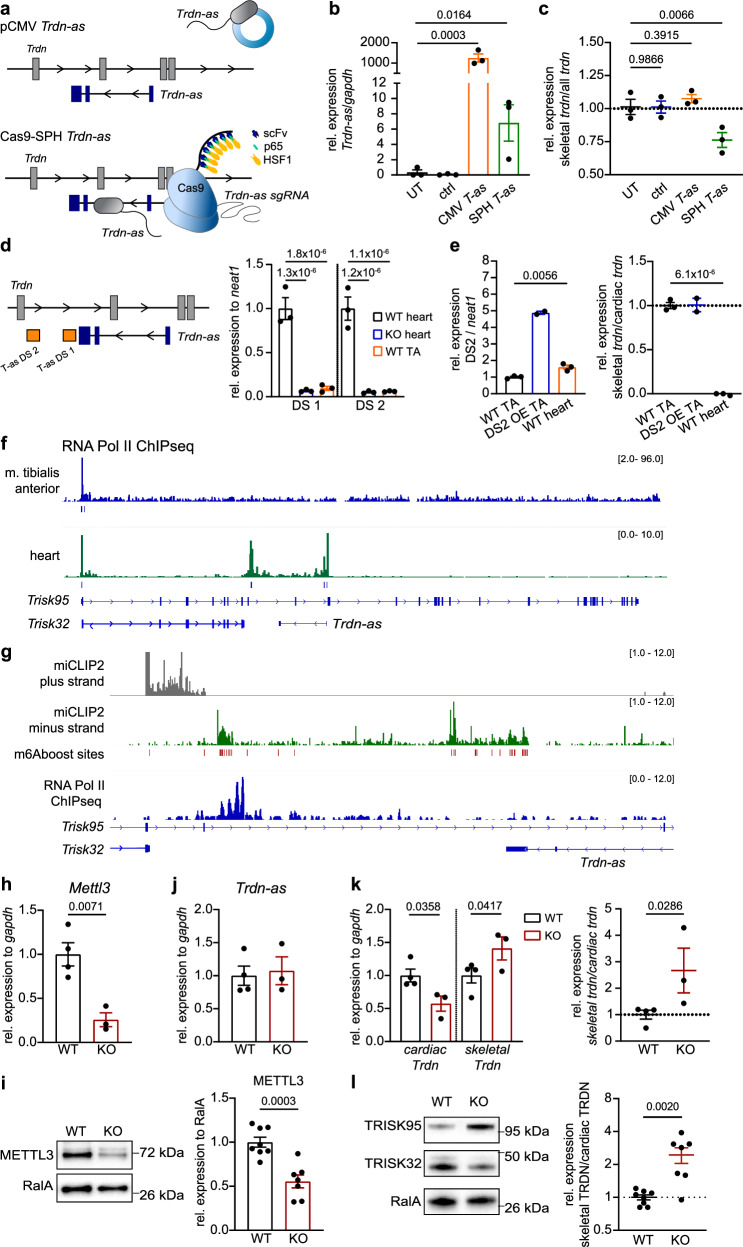
*Trdn-as* acts in *cis* via transcriptional interference and requires METTL3-dependent m6A RNA modifications to generate cardiac-specific *Trdn*-transcripts. **a** Overexpression of *Trdn-as* in isolated mouse muscle stem cells (MuSCs) utilizing a CMV promoter or dCas9-SPH mediated activation of the endogenous promoter. **b, c** RT-qPCR of *Trdn-as* overexpression in differentiated MuSCs and of the respective skeletal *triadin/*all *triadin* ratio (independent transfections: *n* = 3 untransfected (UT), *n* = 3 GFP vector (ctrl), *n* = 3 pCMV-*Trdn-as*, *n* = 3 dCas9-SPH *Trdn-as*, One-way ANOVA, Fisher’s LSD test, mean ± SEM). **d** RT-qPCR of *Trdn-as* derived downstream transcripts (DS1 and DS2) confirming transcription beyond the annotated exons of *Trdn-as* (*n* = 3 WT mice, *n* = 3 *Trdn-as* KO mice, One-way ANOVA, Fisher’s LSD test, mean ± SEM). **e** RT-qPCR using RNA of *tibialis anterior* muscle (TA) and hearts of WT and DS2 transgenic mice (*n* = 3 WT mice, *n* = 2 DS2 OE mice, One-way ANOVA, Fisher’s LSD test, mean ± SEM). **f** RNA polymerase II ChIP-seq of adult WT *tibialis anterior* muscle and adult heart using available data (#ENCFF305EHP, #GSE142518). **g** Published mouse heart miCLIP2 data (#GSE163491) and corresponding predicted m6A sites at the last exons of cardiac *Trdn* and at the extended *Trdn-as* transcript overlapping with RNA polymerase II ChIP-seq signals. **h** RT-qPCR of *Mettl3* expression in *Mettl3* KO heart (*n* = 4 WT hearts, *n* = 3 *Mettl3* KO, two-tailed unpaired Student’s *t* test, mean ± SEM). **i** Western blot analysis of METTL3 protein in heart lysates (*n* = 8 WT hearts, *n* = 7 *Mettl3* KO hearts, two-tailed unpaired Student’s *t* test, mean ± SEM). **j, k** RT-qPCR of *Trdn-as* and *triadin* isoforms in *Mettl3* KO hearts (*n* = 4 WT hearts, *n* = 3 *Mettl3* KO hearts; two-tailed unpaired Student’s *t* test for *Trdn-as*, One-way ANOVA, Fisher’s LSD test for *Trdn*/*Gapdh* ratios, one-tailed Mann-Whitney test for *skeletal Trdn*/*cardiac Trdn* ratios, mean ± SEM). **l** Western blot of TRDN isoform abundance in *Mettl3* KO hearts (*n* = 8 WT hearts, *n* = 7 Mettl3 KO hearts, two-tailed unpaired Student’s *t* test, mean ± SEM).

The localization of *Trdn* and *Trdn-as* in the same locus in an antisense orientation suggests a regulation in *cis*. To provide conclusive evidence for this hypothesis, we expressed *Trdn-as* transcript in *trans* under control of the CMV promoter and activated expression of the endogenous *Trdn-as* locus in *cis* using the dCAS9-SPH system, both in differentiating mouse muscle stem cells (MuSC) (Fig. 7a). Intriguingly, only activation of *Trdn-as* transcription in *cis* but not *trans* shifted the ratio of skeletal/cardiac *Trdn*, although abundance of *Trdn-as* transcripts was much higher in the *trans* compared to the *cis* approach (Fig. 7b, c). Since we detected abundant unspliced transcripts downstream of the annotated *Trdn-as* transcript by RNA-seq and RT-qPCR (Fig. 7d), we generated transgenic mice expressing the *Trdn-as* downstream 2 (DS2) transcript. Despite robust expression of the transgene, we did not detect a significant repression of the skeletal muscle-specific *Trdn* isoform by RT-qPCR in the *tibialis anterior* muscle (Fig. 7e). Taken together, these results indicate that neither the annotated transcript of *Trdn-as* nor downstream sequences of *Trdn-as* repress the skeletal muscle-specific isoform of Trdn in trans. Instead, repression occurs in *cis* via transcriptional activity of *Trdn-as*, as illustrated by the presence of the cardiac-specific *Trdn* in skeletal muscle cells with experimental activation of endogenous *Trdn-as*.

To gain further insights into the mechanisms by which *Trdn-as* prevents the generation of TRISK95 in cardiomyocytes, we analyzed available RNA polymerase II ChIP-seq data for heart and skeletal muscle (heart ChIP-seq, ENCFF305EHP; m. tibialis anterior ChIP-seq, GSE142518). We observed a strong peak for RNA polymerase II at the first exon of *Trdn* in heart and skeletal muscle, indicating promoter-proximal pausing. Proximal-promoter pausing for *Trdn-as* was only observed in the heart, corresponding to the transcriptional activity of *Trdn-as*. Importantly, we also observed pausing of RNA polymerase II at the exon 8-9 region of the *Trdn* gene, most likely due to *Trdn-as* transcription, which terminates at this location (Fig. 7f).

Posttranscriptional modification of RNAs by METTL3/14 methyltransferases is known to occur predominantly at the 3’ UTR of protein-coding transcripts, determining polyA site selection and facilitating termination of transcription^23,24^. Available miClip2 data and m6Aboost annotations of mouse hearts^25^ allowed us to map m6A depositions on transcripts of the *Trdn* genomic locus in a strand-specific manner. We observed strong signals on intronic sense transcripts between exons 8 and 9 of *Trdn*, indicating extensive modification of the *Trdn* pre-mRNA. Interestingly, at the antisense transcript, we also observed strong signals at the end of the annotated *Trdn-as* transcript, but also at the end of *Trdn* exon 9, overlapping with the RNA polymerase II stalling site (Fig. 7g). To investigate the functional role of m6A modifications for the formation of heart and skeletal muscle *Trdn* isoforms, we deleted *Mettl3*, the core component of the m6A writer complex, in cardiomyocytes using *Mettl3*^lox/lox^/αMHC-MerCreMer mice^26,27^. Successful tamoxifen-induced deletion of *Mettl3* in the heart was confirmed by RT-qPCR and Western blot analysis (Fig. 7h, i). The remaining expression of *Mettl3* is most likely caused by the presence of non-cardiomyocytes in the heart. Deletion of *Mettl3* did not alter expression of *Trdn-as* (Fig. 7j), but reduced the cardiac-specific and increased the skeletal muscle-specific *Trdn* transcript, resulting in a significant increase of the skeletal/cardiac transcript ratio (Fig. 7k). Similarly, the TRISK95/TRISK32 protein ratio increased in METTL3-deficient hearts (Fig. 7l), although to a lower extent than observed after deletion of *Trdn-as*. Co-immunoprecipitation of TRDN interacting proteins revealed increased interaction of TRDN with CKAP4/5 and components of the cytoskeleton (Supplementary Fig. S6a).

**Fig. 8:**
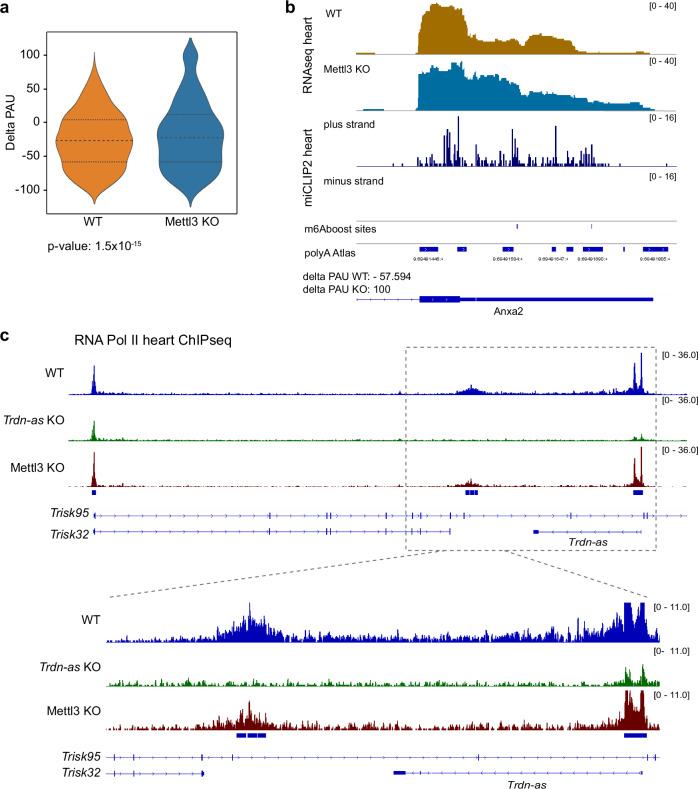
Deposition of m6A determines usage of *triadin* polyA sites and prolongs stalling of RNA polymerase II at the exon 8-9 region. **a** Violin graph comparing differential polyA usage (PAU) of proximal and distal polyA sites between WT and Mettl3 KO hearts. Changes in polyA site use were calculated as delta PAU, with negative values indicating an increase in shorter transcripts (proximal polyA site used) and positive values indicating an increase in longer transcripts (distal polyA site used). Most genes remained unchanged, but a set of genes within the Mettl3 KO group underwent a complete switch (delta PAU 100) from short to long transcripts (two-tailed paired t-test, *p* < 1.6E-15). **b** Representative genome browser view of Anxa2 expression in WT and Mettl3 KO mice using RNA-seq demonstrates complete switch (delta PAU 100) of polyA site usage after inactivation of the m6A writer Mettl3, leading to increased transcript length. Publicly available miCLIP2 heart data (GEO, #GSE163491) was used to visualize m6A modifications. The UCSC polyA atlas (https://polyasite.unibas.ch) was used to indicate potential polyA sites. **c** RNA polymerase II ChIP-seq tracks of adult WT, *Trdn-as* KO and *Mettl3* KO hearts covering the *Trdn* locus. Deletion of *Trdn-as* eliminates the promoter-associated peaks of *Trdn-as* and the peak at the exon 8-9 region, whereas cardiomyocyte-specific deletion of *Mettl3* diminishes the peak associated with the exon 8–9 region.

Since the METTL3/14/VIRMA methyltransferase complex affects selection of polyA sites^28^, we analyzed polyA usage in WT and *Mettl3* KO hearts by RNA-seq. We observed differential usage of polyA sequences in WT and Mettl3 KO hearts (Fig. 8a, b), which was restricted to a subset of transcripts with moderate conservation between mouse cardiomyocytes and human cells (Supplementary Fig. S6b, c). The presence of METTL3 selected for more proximal polyA sites, both in humans as well as in mice, in line with our observations for the TRDN locus. Interestingly, analysis of WT, *Trdn-as* KO, and *Mettl3* KO mice revealed a close correlation between RNA polymerase II stalling and polyA site selection (Fig. 8c). The absence of RNA polymerase II stalling at the exon 8–9 region after abrogation of *Trdn-as* transcription or deletion of *Mettl3* expression exactly matched the shift to an alternate polyadenylation site. However, analysis of the published RNA-seq dataset of failing human hearts (GSE116250) revealed no change in *METTL3* expression, and METTL3 protein was unaltered in DCM patients with arrhythmia compared to DCM patients (Supplementary Fig. S6d–f).

## Discussion

Here, we describe that human cardiomyopathy patients, particularly those with documented arrhythmias, show increased levels of TRISK95 and reduced expression of the non-coding RNA *TRDN-AS*. Mechanistic studies in mice uncovered that *Trdn-as* prevents formation of TRISK51/95, acting in *cis* to stall RNA polymerase II at the exon 8–9 region of the *Trdn* gene, leading to termination of transcription via a m6A-dependent process. *Trdn-as*-deficient mice generating TRISK51/95 instead of TRISK32 in cardiomyocytes display structural remodeling of the dyad and aberrant calcium-release, resulting in QT prolongation, late-onset dilated cardiomyopathy, and increased mortality. The presence of TRISK51/95 in cardiomyocytes changes the interactome of TRDN, thereby affecting the composition and function of the calcium release complex (CRC). The C-terminal domain of TRISK51/95 in mutant cardiomyocytes strongly interacts with CKAP4/CLIMP63 and the related CKAP5, facilitating an interaction of TRDN with the cytoplasmic microtubular network, similar to the situation in skeletal muscle cells^18,20^. Accordingly, we observed pronounced interactions between TRDN and the microtubular network and the actin cytoskeleton. The association of TRISK51/95 with the cytoskeleton shapes the SR terminal cisternae in skeletal muscles, but creates a problem in cardiomyocytes, leading to structural abnormalities.

Altered interactions of TRISK51/95 versus TRISK32 explain the abnormal function of the CRC in *Trdn-as*-deficient cardiomyocytes. We observed reduced interaction of TRDN with CASQ2 and SRI, but increased interaction with HRC. Calsequestrin and HRC are calcium-binding proteins localized in the lumen of the SR, where they interact with the ryanodine receptor and TRDN. Mutations in CASQ2 cause catecholaminergic polymorphic ventricular tachycardia (CPVT)^29,30^. HRC promotes recovery of RYR2 from refractoriness^21^, and mutations in HRC promote spontaneous calcium release by RYR2^31^. In contrast, CASQ2 makes RYR2 more refractory to calcium release, and SRI is an inhibitor of calcium-triggered and spontaneous calcium release by RYR2^22^. Deletion of SRI results in ventricular arrhythmia of mice following isoproterenol stimulation^32^, related to the phenotype of *Trdn-as*-deficient mice, probably due to reduced interaction of TRDN with CASQ2 and SRI. The defects in *Trdn-as*-deficient cardiomyocytes carrying TRISK51/95 instead of TRISK32 emphasize the crucial role of regulatory calcium-binding proteins, which massively impact RYR2-mediated calcium release from the SR. The changes in the interaction of these proteins, caused by the presence of TRISK51/95, increase calcium release and subsequent reuptake in mouse and human cardiomyocytes. A similar condition emerges in gain-of-function mutations of RYR2, causing CPVT^33^, highlighting the importance of precise intracellular calcium-release regulation in cardiomyocytes to maintain excitability of the heart and to prevent cardiac arrhythmia. Consistent with this, we observe altered calcium homeostasis resulting in spontaneous aberrant calcium release events in *Trdn-as*-deficient human and mouse cardiomyocytes. Primary *Trdn-as*-deficient mouse cardiomyocytes also exhibit spontaneous self-terminating calcium oscillations, and *Trdn-as*-deficient hearts show prolonged QT intervals on ECG as well as isoproterenol-induced bradyarrhythmia. Dysfunction of the CRC might be responsible for the development of DCM in *Trdn-as*-deficient hearts, either by affecting calcium handling or by inducing arrhythmias, which are known to have the potential to promote DCM.

Our analysis confirmed the cardiomyocyte-specific expression of *Trdn-as* transcription and further demonstrated that the *Trdn-as* transcript is localized to the nucleus. Analysis of the *Trdn-as* identified binding sites for GATA4, TBX5, SRF, and TEAD1 transcription factors, and expression of GATA4 or TEAD1 increased the activity of a reporter construct for the *Trdn-as* promoter. Since we also observed downregulation of *TEAD1* in human patients with ICM or DCM, we reason that downregulation of TEAD1 in ICM or DCM might be responsible for reduced *Trdn-as* transcription, thereby promoting the appearance of TRISK51/95 and the development of cardiomyopathy in these patients. However, the definitive cause for downregulation of *Trdn-as* in ICM or DCM needs to be addressed in more detail and might entail additional mechanisms. Our study also reveals that the promoter of *Trdn-as* exhibits bidirectional activity, driving expression of both *Trdn-as* and an additional sense transcript of the *Trdn* locus. This sense transcript contains a previously uncharacterized alternative first exon, which is readily detected in cardiomyocytes. The transcript lacks a functional open reading frame and does not code for the C-terminal region of skeletal-muscle TRDN.

It was previously claimed that transcriptional activity of the *Trdn-as* gene is the primary cause of alternative TRDN splicing, resulting in collision of two RNA polymerase II molecules on the two opposing DNA strands^11,12^. In contrast, our stranded RNA-seq analysis suggests precisely coordinated transcriptional interference^34^, governed by antisense transcription specifically at the DNA segment between exon 8 and exon 9 of the sense transcript. In support of this hypothesis, we detected that transcription of *Trdn-as* extends well beyond the annotated *Trdn-as* transcript, reaching the 8th exon of *Trdn*, which is the terminal exon of the TRISK32-coding transcript, containing the 3’ UTR. It is currently not completely clear why the interference occurs at this specific site. Potential reasons are the rather high GC content at the RNA polymerase II stalling site and the existence of a polyA sequence downstream of exon 8. Assembly of a polyadenylation complex may contribute to RNA polymerase II stalling and support transcriptional interference. Similar head-on transcriptional interference phenomena were previously described for ieRNAs (intragenic enhancer-RNAs), where transcription at intragenic enhancers interferes with and attenuates host gene transcription during productive elongation^35^. In the case of the *Trdn* locus, the highly active, cardiomyocyte-specific *Trdn-as* plays the part of the ieRNAs, increasing the RNA polymerase II stalling time, attenuating transcriptional elongation of the sense gene, and thereby generating a shortened transcript. Interestingly, a previous mouse model with deletion of exons 2 and 3 of *Trdn-as* showed no TRISK95 induction^12^. This suggests the deletion of *Trdn-as* exons did not disrupt as-transcription or transcriptional interference at the 8th exon of *Trdn*. This supports the view that transcriptional interference, not the *Trdn-as* RNA molecule, is crucial for the TRDN-isoform shift in cardiomyocytes.

Termination of transcription and polyadenylation apparently also play a role in this process, since we disclosed a critical function of the methyltransferase METTL3 for suppression of TRISK95 transcripts. METTL3 modifies RNAs by m6A methylation, which is one of the most prevalent co-transcriptional RNA modifications, predominantly occurring in 3’ UTRs of transcripts^23,36^. METTL3 is also known for its decisive role in the termination of transcription by introducing m6A RNA modifications^37^ and for the selection of more proximal, alternative polyA sites^28^. We detected putative m6A-sites at the 3’ end of the annotated as-transcript and downstream of *Trdn* exon 9, which matched publicly available miCLIP2 data for m6A modifications^25^. Moreover, we observed massive m6A-methylation of the sense transcript, precisely within the intronic region of exons 8 and 9 and consistent with the 3’ UTR of the TRISK32-coding transcript. Importantly, inactivation of *Mettl3* in adult cardiomyocytes selected an alternative, more distal polyA-site and profoundly changed TRDN isoform expression, without altering expression of *Trdn-as*. We concluded that repression of the skeletal muscle-specific *Trdn* transcript has different requirements: antisense transcription in a cell-specific manner, *cis*-acting determinants exactly determining the site of transcriptional interference between exon 8 and exon 9, and recruitment of the METTL3/14/VIRMA complex to support polyadenylation at exon 8. Previous reports described cardiac dilation in older mice after deletion of *Mettl3*, but did not provide a convincing explanation^38^. We propose that the appearance of TRISK51/95 in *Mettl3*-deficient cardiomyocytes might contribute to the late-onset cardiac phenotype of *Mettl3*-mutants, although other functions of *Mettl3* may be more relevant for the phenotype.

The presence of skeletal muscle-specific TRISK95 in human patients with DCM, in combination with our mechanistic studies in human iPSC-derived cardiomyocytes and different mouse models indicate that alterations of the CRC, instigated by a failed TRDN isoform switch, are highly relevant for patients with cardiomyopathies and idiopathic arrhythmia. We postulate that downregulation of *TRDN-AS* and the accompanying TRDN-isoform shift does not necessarily have to occur across the myocardium. Even a spatially restricted expression of TRISK95, associated with local dysfunction of the CRC, might be sufficient to induce electrophysiological abnormalities in the heart due to the electrical coupling of the myocardium. In our model, electrophysiological alterations, including QT prolongation and transient bradyarrhythmia, are observed prior to overt structural remodeling, suggesting that the altered calcium handling may cause these changes. At the same time, cardiomyopathy may further exacerbate these electrophysiological changes, indicating a complex interplay between calcium dysregulation, electrical disturbances, and structural remodeling. So far, no serum markers are available to identify patients at risk for arrhythmia due to abrogation of the TRDN-isoform switch. TRISK95 is abundantly present in skeletal muscle and thus cannot be easily employed for testing. Even cardiac biopsies might fail if regions of the heart are missed, in which TRISK95 emerges. Thus, further studies and the systematic assessment of the mechanism described here are necessary to identify patients at risk who will profit from additional interventions to prevent fatal arrhythmias.

## Methods

### Patients and endomyocardial biopsies

Patients with non-ischemic dilated cardiomyopathy were enrolled in the subproject TP9a of the German Competence Network Heart Failure from 2004 to 2008. All patients underwent a comprehensive evaluation, including a detailed medical history, clinical examination, laboratory testing, and echocardiographic assessment. Coronary angiography was performed in all cases to exclude an ischemic etiology of cardiac dysfunction. Additionally, patients with systemic inflammatory or autoimmune diseases, sarcoidosis, or amyloidosis were excluded^39^. From this study cohort, patients with available endomyocardial biopsies (EMB) and a history of sustained ventricular tachycardia, sustained supraventricular tachycardia, and those without a history of arrhythmia were identified and included for further analysis. EMBs were obtained from the left ventricle as described^39^. The classification of arrhythmias was carried out by experienced clinicians (specialist training in cardiology, subspecialization in rhythmology), according to the corresponding guidelines of the European Society of Cardiology^40,41^. The study protocol was approved by the German Competence Network Heart Failure and conducted in accordance with the Declaration of Helsinki (1996), the International Conference on Harmonization Good Clinical Practice (ICH-GCP), and the Ethics Committee of the University Hospital of Marburg.

Additional myocardial samples were obtained from the Heart and Diabetes Centre NRW Bad Oeynhausen. All patients had a confirmed clinical diagnosis of non-ischemic dilated cardiomyopathy (DCM) and were categorized according to the presence or absence of previously recorded arrhythmia. The study conformed to the rules of the Helsinki declaration. The study was approved by the local Ethics Committee of the Ruhr-University Bochum situated in Bad Oeynhausen, Germany (Reg. No. 2025-1371).

All patients included in the study provided written informed consent and received no compensation. The study protocols exclude a self-selection bias. Details on potential covariate-relevant population characteristics (age, genotypic information, treatment) were not considered in this study due to the limited number of patients who were included in the study. A control tissue lysate of non-failing human heart tissue was obtained from Abcam (ab29431, Lot GR238526-9). The ratios of TRISK95/TRISK32 were normalized to the ratios of the DCM + arrhythmia samples of the respective blots.

### Animal models

To generate *Trdn-as* loss-of-function mice, a polyA-site was inserted into the first exon of *Trdn-as* using a targeted Cas9 endonuclease approach^14^. To achieve homology-directed repair, a specific guide sequence targeting the first exon of *Trdn-as* (gCTATAAGCTTGGACTCCATGGG) was used in combination with a single-stranded oligo containing flanking homologous sequences and a polyA signal sequence (GAATTCTGGTTACAAATAAAGCAATAGCATCACAAATTTCACAAATAAAGCATTTTTTTC). Successful recombination of alleles was verified by PCR of individual mouse embryonic stem cell clones using a specific primer pair (TTAGTCTGGGAAAGTGACAAATGGC; TTGGACCTGGTGACATTCATGTGGC) as well as by Sanger sequencing. Embryonic stem cells were injected into blastocysts to generate chimeric mice, which were backcrossed to C57BL/6 mice (Charles River) and used to obtain homozygous *Trdn-as* KO mice.

Forced expression of the *Trdn-as* downstream transcript 2 (*Trdn-as* DS2) in *tibialis anterior* muscle was achieved by pronuclear injection of an expression cassette consisting of a CMV promoter and 1 kb genomic sequence (GRCm38/mm10, chr10:33200367-33201366 (-)) coding for the DS transcript. The construct was generated by gene synthesis and insertion into the pCDNA3.0 backbone (BioCat GmbH). The vector was digested using NruI and XhoI restriction endonucleases, and the released expression cassette was purified for subsequent pronuclear injection. Transgenic mice were screened by PCR for the presence of the transgene using CMV promoter-specific oligonucleotides (GACGTCAATGGGTGGACTATTTACG, CCATTTGCGTCAATGGGGCGGAGT).

CAG-LSL-dCas9-SunTag-p65-HSF1^pos^: Pax7^pos^ mice^42–44^ were used for ectopic expression of *Trdn-as* from its endogenous locus in isolated differentiating muscle stem cells.

Mettl3^lox/lox^: αMHC-MerCreMer^pos^ and Mettl3^lox/lox^ control mice^26,27^ aged between 10 and 16 weeks were injected intraperitoneally on seven consecutive days with 100 µg tamoxifen/1 g body weight. Tamoxifen was dissolved in medium-chain triglycerides (Miglyol 812, Caelo).

Ultra-purified custom-made AAV-MYOAAV2A virus containing cTnT> mTrdn[NM_029726.2]: T2A: mCherry: WPRE expressing TRISK95 and control cTNT> mCherry: WPRE were provided by Vectorbuilder. WT mice were tail vein injected with 1 × 1012 AAV GC (vector genome copies)^45^.

Mice were kept in individual ventilated cages with unlimited food and water, light from 7 a.m. to 6 p.m., a temperature range of 20 °C to 24 °C, and a humidity range of 45 to 65%. All animal experiments were conducted in compliance with national and European community guidelines and received approval from the Committee for Animal Protection of the State of Hessen (Regierungspraesidium Darmstadt).

### Promoter analysis

For *Trdn-as* promoter reporter vectors, a Dual Reporter vector was used (pGL4Luc-RLuc was a gift from Daniel Christophe by Addgene plasmid #64034; http://n2t.net/addgene:64034; RRID: Addgene_64034)^46^. *Trdn-as* promoter (GRCm39: chr10:33132331-33132599) or promoter-enhancer sequences (GRCm39: chr10:33101989-33102071, chr10:33130033-33130716, chr10:33132178-33132599) and mutants for TEAD1 binding sites (Supplementary sequence data) were obtained by gene synthesis (BioCat) and transferred into the reporter vector using NheI-HindIII or NheI-XhoI sites, respectively. HEK-293 cells (ATCC #CRL-1573) were transfected with a GFP-expressing control vector, the empty Dual Reporter vector, or the vectors containing the promoter or promoter/enhancer sequences using Lipofectamine 3000 (Thermo#L3000008). For additional expression of transcription factors, vectors expressing TEAD1 (hORFeome V8.1 Library), GATA-4 (hORFeome V8.1 Library), or MEIS1 (BioCat gene synthesis Uniprot: Q60954-1 in pIRES2EGFP) were co-transfected together with Dual Reporter vectors. Cells were lysed 48 h after transfection, Luciferase and Renilla luminescence were quantified using the Dual-Luciferase® Reporter Assay System (Promega, #E1910) and Mithras LB940 plate reader with MicroWin 2000 (Berthold Technologies).

### Isolation of mouse cardiomyocytes

Adult mouse cardiomyocytes were isolated as described^14^. Mice were sacrificed by i.p. injection of Ketamin/Xylariem, hearts were collected, and cardiomyocytes were isolated using the Langendorf perfusion system. Hearts were washed using perfusion buffer (113 mM NaCl; 4.7 mM KCl; 0.6 mM Na_2_HPO_4_; 0.6 mM KH_2_PO_4_; 1.2 mM MgSO_4_ × 7H_2_O; 12 mM NaHCO_3_; 10 mM KHCO_3_; 30 mM taurine; 10 mM HEPES; 10 mM 2,3-Butanedione monoxime; 5.5 mM glucose) and cells collected by digestion of the heart using enzyme buffer (perfusion buffer, Liberase DH 0.25 mg/ml (Roche, #05401089001), Trypsin 0.27 mg/ml (Sigma, #T7409), 23.2 μM CaCl_2_). To stop the enzymatic reaction, stop-buffer (perfusion buffer, FCS 5%, 12.5 μM CaCl_2_) was added to the cell lysate. Cardiomyocytes were treated with an ascending CaCl_2_ series (50 μl 10 mM; 50 μl 10 mM; 100 μl 10 mM; 30 μl 100 mM; 50 μl 100 mM) in 8 min intervals in perfusion buffer. Subsequently cardiomyocytes were filtered using a 100 µm cell strainer, briefly centrifuged at 500 × *g* for 1 min at RT and taken up in culture medium (M199 (Life Technologies, 11150-059); 5 mM Creatine × H_2_O; 2 mM L-Carnitine × H_2_O; 5 mM taurine; 25 mM HEPES; 5% FCS; 1% PS; 1% ITS supplements; 10 μM AraC: pH 7.3 with NaOH). Single adult cardiomyocytes were either plated on laminin (10 µg/ml PBS) coated 12-well chamber slides (ibidi, #81201) for RNA-FiSH or on 25 × 25 mm^2^ coverslips for IonOptix experiments.

### Quantification of cardiomyocyte cross-sectional area

To determine cross sections of cardiomyocytes, hearts were collected after cervical dislocation and fixed in 4% PFA/PBS for 2 h at 4 °C. Fixed hearts were incubated with 15% sucrose/PBS, 30% sucrose/PBS, and Tissue-Tek® O.C.T. Compound (Surgipath, FSC 22 Clear, #3801480) each overnight at 4 °C before sectioning was performed using the Leica CM3050 cryotome. Cryosections were dried for 30 min at RT, fixed again with 4% PFA/PBS for 10 min at RT, and washed three times with 0.3% Triton X-100/PBS. Sections were rinsed once with PBS, blocked for 1 h using Blocking Solution (Blocking One (Hikari, Nacalai tesque), 0.01% Triton X-100/PBS) and stained using wheat germ agglutinin (WGA-488, ThermoFisher #W11261, 1 mg/ml in Solution A (Hikari, Nacalai tesque)) overnight at 4 °C. On the next day, sections were washed three times with 0.01% Triton X-100/PBS for 10 min each, and nuclei were stained using DAPI (Invitrogen #D3571, 2 mg/ml, 1:1000 in PBS) for 5 min at RT. Sections were washed three times with PBS and embedded in Fluoromount (Sigma #F4680). Microscopic images were acquired using a Zeiss Axio observer and the cross-sectional area of cardiomyocytes was determined using manual segmentation in ImageJ. To cluster cardiomyocytes depending on cross-sectional areas, numerical binning was used for statistical analysis.

### Fluorescence in-situ hybridization (FiSH)

RNA FiSH was performed using the Stellaris RNA FiSH system (LGC Biosearch Technologies) using a pool of 30 RNA FiSH probes against the *Trdn-as* lncRNA (Supplemental Table 2). The probe set was designed using the Stellaris RNA FiSH Probe Designer and the sequence of the spliced transcript of *Trdn-as*. Each probe is individually labeled with the fluorescent dye Quasar570. Adult CMs were cultured in a 12-well silicone chamber (Ibidi GmbH, #81201) for 2 h and fixed in 3.7% PFA in 1x PBS for 15 min at room temperature. Cells were rinsed twice with GIBCO^TM^ DPBS (ThermoFisher, #14190144) and permeabilized with 70% ethanol for 1 h at 4 °C. After washing the cells once with Wash Buffer A (Cat#SMF-WA1-60) for 5 min at room temperature, a humidified box was assembled for hybridization of the cells with 100 µl of hybridization buffer (Cat#SMF-HB1-10) containing 125 nM of the RNA FiSH probe set. Hybridization was performed in a heating chamber at 37 °C for at least 16 h in the dark. Afterwards, cells were washed twice using Wash Buffer A for 30 min at 37 °C in the dark, with DAPI added 1:1000 for the last 10 min to achieve nuclear staining. After the final 5 min washing step using Wash Buffer B (Cat#SMF-WA1-20) at room temperature, the silicone chamber was removed and the cells sealed using ProLong Gold Antifade Mounting reagent (ThermoFisher, #P10144) and a cover glass. Slides were kept in the dark for 48 h before imaging was performed using a Zeiss Axio Imager Z1.

### Telemetric measurements

Telemetric measurement of ECG was performed^47^ using TA10EA-F20 implantable transmitters (Data Science International). Transmitter electrodes were placed subcutaneously on the right and left sides of the thorax. Data were sampled using Dataquest A.R.T 4 with a sample rate of 1 kHz and collected from undisturbed animals overnight and after i.p. injection of 1 µg/g bodyweight isoproterenol (Sigma Aldrich, #16504). Data were analyzed using Labchart 7Pro (ADInstruments, v7.3.8), including the ECG analysis module (v2.3.2). Segments of 20 beats were averaged, and a total of 180 averaged beats/mouse was used for the evaluation of ECG intervals. Heart rate was measured overnight from 8:00 pm to 6:00 am every 10 min for 1 min or in case of isoproterenol injection 10 min before injection until 30 min after injection every 5 min.

### Cardiac magnetic resonance imaging

Cardiac magnetic resonance imaging (MRI) was performed^47^ on a 7.0 T Bruker Pharmascan (Pharmascan 70/16, Bruker, Ettlingen, BRD) equipped with a 760 mT/m gradient system, using a cryogenically cooled four-channel phased array element 1H receiver coil (CryoProbe), a 72-mm room-temperature volume resonator for transmission, and the IntraGate self-gating tool^48^. The assessment utilizes the gradient echo technique with parameters set as follows: a repetition time of 6.2 ms, an echo time of 6.0 ms, a field of view measuring 2.20 × 2.20 cm^2^, a slice thickness of 1.0 mm, a matrix size of 128 × 128, and 100 repetitions. The imaging plane was determined using preliminary images that displayed the heart’s 2- and 4-chamber views, succeeded by capturing images in the short-axis view, perpendicular to the septum in both preliminary images. A series of contiguous short-axis slices, ranging from 7 to 10, was obtained to ensure complete visualization of the left and right ventricles. During MRI measurements, mice were anaesthetized with 1.5 to 2.0% isoflurane in 0.5 l/min air, and 0.5 l/min oxygen was supplied via an inhalation mask, while body temperature was maintained at 37 °C via a thermostatically regulated water flow system. Acquired MRI data were analyzed using the Qmass Mass4Mice digital imaging software (Medis Imaging Systems, Leiden, Netherlands).

### Transmission electron microscopy

Transmission electron microscopy was carried out by Dr. Ulrich Gärtner at the EM facility of the medical faculty of the Justus-Liebig-University Giessen. In brief, whole hearts were perfused with 1x PBS and fixed in a solution containing 1.5% PFA (Sigma, #6148), 1.5% glutaraldehyde (Sigma, #G5882), and 0.15 M HEPES (Sigma, #H3375) for at least 24 h. Fixed hearts were stained using 1% osmium tetroxide solution, embedded in epoxy resin, and underwent ultrathin sectioning. Imaging was done on a Zeiss EM 902. Transmission electron microscopy images were analyzed using Fiji (ImageJ)^49^. Constricted or completely collapsed dyads, defined by massively reduced/absent jSR density, discontinuous junctional membranes, or misalignment of the jSR relative to T-tubules, were counted and excluded from quantitative analyses. Junctional gap width (Junction) and junctional sarcoplasmic reticulum (jSR) width were quantified by averaging at least seven orthogonal measurements per dyad. Measurements were restricted to classical dyads exhibiting continuous and parallel membrane apposition between T-tubules and jSR.

### RNA-seq and RT-qPCR

For RT-qPCR, RNA was isolated from snap-frozen tissues or freshly cultivated cells using the TRIzol method. For cDNA synthesis, 1 µg of tissue and 200 ng of cell RNA was used in combination with the Takara PrimeScript RT-PCR Kit with gDNA eraser (Takara, #RR047A) following the manufacturer’s recommendations. RT-qPCR was performed using the Blue S´Green qPCR Kit (Biozym. #F410-L/F415-L) in combination with a StepOne Plus System (ThermoScientific, #4376600). RT-qPCR oligonucleotide sequences are listed in Supplemental Table 1.

Nuclear and cytoplasmic RNA was recovered from isolated mouse cardiomyocytes and differentiated C2C12 cells. After washing with ice-cold PBS, cells were centrifuged at 1000 × *g* for 10 min at 4 °C, and the pellet was resuspended in buffer A (10 mM Tris-HCL pH8.0; 140 mM NaCl; 1.5 mM MgCl_2_; 0.5% Nonidet P-40). Samples were incubated for 5 min on ice, centrifuged again at 1000 × *g* for 3 min at 4 °C, and the supernatant containing the extra nuclear fraction was collected and mixed with TRIzol. The remaining pellet was washed twice with buffer A and then re-suspended in buffer B (Buffer A + 1% Tween-40 and 0.5% deoxycholic acid). Following another centrifugation for 3 min, the pellet containing the nuclear fraction was taken up in TRIzol for RNA isolation. For RNA-seq, RNA was isolated by miRNeasy mini Kit (Qiagen, #217004) combined with on-column DNase digestion (RNase-Free DNase Set, Qiagen, #79254) to avoid contamination by genomic DNA. RNA and library preparation integrity were verified with LabChip Gx Touch (Perkin Elmer).

### Library construction

RNA amounts were normalized, and 500 ng (*Trdn-as* KO) or 1 µg (*Mettl3* KO) of total RNA was used as input for SMARTer Stranded Total RNA Sample Prep Kit HI Mammalian (Takara Bio). Sequencing was performed on the NextSeq500 platform (Illumina) using P3 flowcell with 75 bp single-end setup (*Trdn-as* KO) or on the NextSeq2000 platform (Illumina) using P4 flowcell with 72 bp single-end setup (*Mettl3* KO).

### RNA-seq analysis

Trimmomatic version 0.39 was employed to trim reads after a quality drop below a mean of Q15 in a window of 5 nucleotides and keeping only filtered reads longer than 15 nucleotides^50^. Reads were aligned versus Ensembl mouse genome version mm10 (GRCm38) with STAR 2.6.1 d (*Trdn-as* KO) or versus Ensembl mouse genome version mm39 (Ensembl release 109) with STAR 2.7.11a (*Mettl3* KO)^51^. Alignments were filtered to remove: duplicates with Picard 2.21.1 (*Trdn-as* KO) or Picard 3.1.1 (*Mettl3* KO) (Picard: A set of tools (in Java) for working with next-generation sequencing data in the BAM format), multi-mapping, ribosomal, or mitochondrial reads.

Gene counts were established with featureCounts 1.6.5 (*Trdn-as* KO) or featureCounts 2.0.4 (*Mettl3* KO) by aggregating reads overlapping exons on the correct strand, excluding those overlapping multiple genes^52^. The raw count matrix was normalized with DESeq2 version 1.18.1 (*Trdn-as* KO) or DESeq2 version 1.36.0 (*Mettl3* KO)^53^. Contrasts were created with DESeq2 based on the raw count matrix.

### Analysis of polyA utilization

To examine polyA utilization, we used the QAPA software (version 1.3.3)^54^. By utilizing Gencode polyA sites and additional sites from the polyA database (https://polyasite.unibas.ch), we generated a genome-wide UTR file. Next, we used salmon (v1.10.0) (https://github.com/COMBINE-lab/salmon)^55^ to generate counts for quantification of 3′ UTR isoforms. We performed one run per condition (WT/KO) and summarized the four replicates for each on the count level. Finally, we calculated the PAU (in %) and the TPM per gene per UTR isoform via the QAPA quant module. For downstream analysis, we used Python to filter UTRs with a TPM > 1, filtered for genes with exactly 2 UTR isoforms in use, calculated a delta PAU per condition (WT / KO) by subtracting the short UTR usage from the long UTR usage, and plotted violins via Seaborn. The mm10 assembly was used for all UTR analysis.

### Chromatin immunoprecipitation (ChIP-seq)

ChIP-seq of mouse ventricles was performed using the Diagenode iDeal ChIP-seq kit for Transcription Factors (Diagenode, #C01010055) in combination with the Diagenode RNA Pol II antibody recognizing the B1 subunit (Diagenode, #C15200253) according to the manufacturer’s recommendation with minor changes. Snap-frozen ventricles from two adult mouse hearts were thawed in 3 mL of ice-cold lysis buffer (5 mM CaCl₂, 3 mM MgAc, 2 mM EDTA, 0.5 mM EGTA, 10 mM Tris-HCl pH 8.0), protease inhibitor cocktail Roche, (Roche, #04693159001), RNasin (Promega, #N2611, 10 μl/ml) for 5 min and the tissue homogenized using the gentleMACS Dissociator (Miltenyi Biotec). Triton X-100 was added to a final concentration of 0.2% and the suspension was sheared by repeated aspiration through a syringe on ice. The homogenate was filtered through a 40 µm cell strainer, centrifuged for 5 min at 1000 × *g*, 4 °C, resuspended in 800 µL of lysis buffer with 0.2% Triton X-100 and layered onto 3 mL of 1 M sucrose in a 15 mL tube. After centrifugation (5 min at 1000 × *g*, 4 °C) nuclei were taken up in fixation buffer containing 1% formaldehyde for 8 min at RT with gentle rotation for cross-linking. Nuclei were washed in ice-cold PBS, then sequentially lysed using lysis buffers iL1b for 20 min at 4 °C and iL2 for 10 min at 4 °C, each supplemented with protease inhibitor cocktail (1:200, Diagenode). The lysed nuclei were resuspended in shearing buffer iS1b (Diagenode) containing protease inhibitors and incubated on ice for 10 min. Samples were aliquoted into microtubes and subjected to sonication using a Bioruptor Pico (Diagenode) to shear chromatin to an average length of 200–600 bp. After centrifugation (10 min at 16,000 × *g*, 4 °C), supernatants containing sheared chromatin were pooled. Protein A magnetic beads were prewashed with ChIP buffer iC1b and incubated with ChIP reaction mix containing either anti-RNA polymerase II antibody, CTCF positive, or IgG negative control. Bead-antibody complexes were incubated for 4 h at 4 °C with rotation, followed by addition of 250 µL of sheared chromatin. Input controls (2.5 µL) were retained for normalization. Chromatin immunoprecipitations proceeded overnight at 4 °C. Beads were washed with wash buffers iW1 through iW4. Chromatin complexes were eluted from beads with 100 µL of buffer iE1 for 30 min at room temperature, followed by addition of 4 µL of buffer iE2. Input samples were treated identically. All samples were incubated at 65 °C for 4 h to reverse cross-link. Following addition of a DNA carrier and isopropanol, DNA was purified using IPure v3 magnetic beads according to manufacturer instructions. DNA was eluted in 25 µL of buffer C and quantified by Qubit dsDNA HS Assay Kit (ThermoFisher Scientific).

Library Construction Protocol: A maximum of 10 ng of DNA per sample was used as input for TruSeq ChIP Library Preparation Kit (Illumina) with following modifications. Instead of gel-based size selection before final PCR step, libraries were size selected by SPRI-bead based approach after final PCR with 18 cycles. In detail, samples were first cleaned up by 1x bead:DNA ratio to eliminate residuals from PCR reaction, followed by 2-sided-bead cleanup step with initially 0.6x bead:DNA ratio to exclude larger fragments. Supernatant was transferred to new tube and incubated with additional beads in 0.2x bead:DNA ratio for eliminating smaller fragments, like adapter and primer dimers. Bound DNA samples were washed with 80% ethanol, dried and resuspended in TE buffer. Library integrity was verified with LabChip Gx Touch 24 (Perkin Elmer). Sequencing was performed on NextSeq2000 platform (Illumina) using P3 flowcell with 72 bp single-end setup.

ChIP-seq analysis: Trimmomatic version 0.39 was employed to trim reads after a quality drop below a mean of Q15 in a window of 5 nucleotides and keeping only filtered reads longer than 15 nucleotides^50^. Reads were aligned versus Ensembl mouse genome version mm39 (Ensembl release 109) with STAR 2.7.11b^51^. Alignments were filtered to remove: duplicates with Picard 3.1.1 (Picard: A set of tools (in Java) for working with next generation sequencing data in the BAM format), spliced, multi-mapping, ribosomal, or mitochondrial reads. Peak calling was performed with Macs version 3.0.0a7 with FDR < 0.001 and enrichment vs. input > 2 × ^56^. Peaks overlapping ENCODE blacklisted regions (known misassemblies, satellite repeats) were excluded. Remaining peaks were unified to represent a common set of regions for all samples. Counts were produced with featureCounts^52^. The raw count matrix was normalized with DESeq2 version 1.36.0 (Love et al., Moderated estimation of fold change and dispersion for RNA-Seq data with DESeq2). Peaks were annotated with the promoter (TSS ± 5000 nt) of the nearest gene based on Ensembl release 109.

### Calcium transient and contraction analysis

The Myocyte Calcium and Contractility System IonOptix (IonOptix 2006, Westwood, MA, USA) along with the ratiometric calcium dye Fura-2-AM (ThermoFisher Scientific, #F1201) were used to investigate calcium transients in isolated mouse and hiPSC-derived cardiomyocytes. Cardiomyocytes were isolated from adult, 12-weeks-old mice and plated on 25 × 25 mm^2^ glass-coverslips prior covered with 10 µg/ml PBS laminin. Fura-2-AM was stored as 1 mg/ml stocks in DMSO at -20 °C and diluted 1:500 in buffer EB (EB: 11 mM NaCl; 2.8 mM KCl; 0.6 mM MgCl_2_; 1.2 mM KH_2_PO_4_; 1.2 mM CaCl_2_; 10 mM HEPES; 20 mM Glucose; pH 7.3). Cardiomyocytes were incubated with Fura-2-AM in EB for 15 min in the dark whilst slow shaking, afterwards rinsed twice with EB and left in the dark for 20 min at 37 °C to allow an equal distribution of the dye. Baseline calcium transients were recorded using the IonOptix MyoPacer Field Stimulator (IonOptix 2006, Westwood, MA, USA) with 1 Hz field stimulation at 20 V and 5 msec duration. Multiple cardiomyocytes were measured per animal, with a recording time of at least 10 transients/cardiomyocyte. Calcium transients, sarcomere, and whole cell shortening were analyzed using the IonWizard (IonOptix, Version 6.3) Software. All recorded cardiomyocyte measurements were screened for aberrant calcium cycling.

### Western blot analysis and membrane protein isolation

Whole heart protein lysates for immunoprecipitation, western blot, and mass spectrometry analysis were obtained by mincing snap-frozen hearts using a mortar on dry ice. The minced tissue was taken up in 1 ml of RIPA buffer (50 mM Tris-HCl pH 7.4, 1% NP-40, 0.25% sodium deoxycholate, 150 mM NaCl, 1× protease inhibitor Cocktail 3 (Merck, #539134), 1 U RNasin (Promega, #N2611)) and sonicated (30 power/5 cycle/20 sec). For cardiac membrane protein isolation, snap-frozen hearts were minced using a mortar on dry ice, and the sample was collected in 700 μl of homogenizing buffer (125 mM NaCl; 20 mM Tris/HCl, pH 8.0; 4.5 mM EDTA pH 8.0; 1x Complete (Roche, #04693159001)). After sonication, the lysate was centrifuged at 1000 × *g* for 5 min at 4 °C and the supernatant was centrifuged at 135,000 × *g* for 1 h at 4 °C. The pellet containing the membrane protein fractions was dissolved in 200 µl sample buffer (RiPA; 4 mM pefablock; 1x Complete) and used for western blot and mass spectrometry analysis. Protein concentrations were assessed using the BioRad DC protein assay kit (BioRad, #5000111). For western blot analysis 10 µg of protein lysate per lane was loaded onto a 4–12% BisTris SDS PAGE (Invitrogen, #NP0321BOX) in 1x Laemmli buffer. For blotting, a nitrocellulose membrane (GE Healthcare, Protran BA85) was used, and blocking was carried out using 5% milk/TBS-T. The following primary and secondary antibodies were used in 3% BSA/TBS-T: anti-TRISK32, anti-TRISK51 (gifted by Dr. I. Marty,^57^, 1:10000), anti-TRDN mouse and human (Abcam, #ab247008, 1:500), anti-human TRDN (LifeSpan BioSciences, #LS-C749822, 1:500), anti-Mettl3 (Abcam, #ab195352, 1:1000), anti-GAPDH (Cell Signaling Technology, #2118, 1:2000), anti-cTNNI3 (Abcam, #ab56357, 1:1000), anti-RalA (BD, #610221, 1:5000), anti-mouse HRP (Pierce #1858413, 1:10000), anti-rabbit HRP (Pierce #31460, 1:10000). SuperSignal™ West Femto Maximum Sensitivity Substrate (ThermoFisher, #34095) was used with a ChemiDoc imaging system (BioRad).

### Immunoprecipitation

Immunoprecipitation (IP) was carried out as previously^14^. In brief, 2 mg of heart protein lysate was combined with 30 µl of G-Sepharose-beads (Sigma Aldrich, #P3296) and 2 µg of anti-Triadin (Abcam, #ab247008) or IgG (Cell Signaling, #2729S) antibody for overnight incubation at 4 °C. After 3 times of washing with RIPA buffer, beads were taken up in 200 µl RIPA buffer and used for Western blot and mass spectrometry analysis. In case of western blotting, an anti-rabbit IgG Trueblot HRP antibody (Ebioscience, #18-8816-31, 1:5000) was used.

### Mass spectrometry

Heart tissue was homogenized in 4% SDS, 100 mM Tris, pH 7.6 with protease inhibitors. Solubilized proteins were precipitated by 80% acetone at −20 °C overnight. Precipitated proteins were resolubilized in digestion buffer (6 M Urea/2 M thiourea), reduced with 10 mM DTT for 30 min at RT, and alkylated with 55 mM IAA for 30 min at RT/Dark. Each sample (100 µg) was digested with 10.4 µg of Lys-C for 3 h at RT, diluted with 330 µl of 0.1 M TEAB and subsequently digested with 2 µg of trypsin overnight. Digested peptides were then fractionated to 8 fraction using a High pH Reversed-Phase Peptide Fractionation Kit (Thermo Scientific, Pierce).

For membrane proteomics proteins were extracted by 4% SDS, 100 mM Tris, pH 7.6 with protease inhibitors. Solubilized proteins were precipitated by 80% acetone at −20 °C overnight. Precipitated proteins were resolubilized in digestion buffer (6 M Urea/2 M thiourea), reduced with 10 mM DTT for 30 min at RT and alkylated with 55 mM IAA for 30 min at RT/Dark. Each sample (100 µg) was digested with 10.4 µg of Lys-C for 3 h at RT, diluted with 330 µl of 0.1 M TEAB and subsequently digested with 2 µg of trypsin overnight.

For mass spectrometry of immunoprecipitated samples, washed beads were mixed with 30 µl digestion buffer (6 M Urea/2 M thiourea), reduced with 10 mM DTT for 30 min at RT, and alkylated with 55 mM IAA for 30 min at RT in the dark. Each sample was diluted with 60 µl of 0.1 M TEAB and digested with 0.5 µg of trypsin overnight. Digested proteins were desalted using a C18 stage tip. Desalted peptides were measured by LC-MS/MS.

For all samples, quantitative analyses were performed on an Orbitrap Q-exactive HF mass spectrometry system (Thermo Scientific) coupled to an EASY-nLC capillary nano-chromatography system (Thermo Scientific). Desalted peptides were separated on an in-house-packed capillary column (150 mm × 1.7 µm × 75 µm) using ReproSil-Pur 120 C18-AQ resin (Dr. Maisch). The mobile phases were (A) 2% Acetonitrile, 0.1% formic acid (B) 90% acetonitrile, 0.1% formic acid. Peptides were separated using a 230 min (150 min for heart-MS) acetonitrile gradient at room temperature. The mass spectrometer was operated in positive electrospray ionization (ESI) mode, and MS/MS data were collected in data dependent (data independent for *Mettl3* KO TRDN-IPs) analysis mode with a resolution of 60,000 for precursor mass spectra and 15,000 (30,000 for DIA) for tandem mass spectra. Normalized collision energy was set to 28 and exclusion time was set to 30 s. Collected data were processed using Maxquant software (FragPipe for *Mettl3* KO TRDN-IPs).

### FACS based isolation of MuSC, cell culture and transfection of cells

Isolation and culture of primary mouse muscle stem cells (MuSC; satellite cells; SC) derived from skeletal muscle tissue was done as described with minor changes^44,58^. Skeletal muscle tissue was dissected from individual animals in DMEM (Merck, #D5796) containing 2% Penicillin-Streptomycin. After tissue-chopping, the muscle mass was digested using Dispase (BD, #354-235) and Collagenase type II (Worthington, #LS004185) for 1 h in a 37 °C shaker. Ten percent FCS was added, and the suspension was centrifuged briefly at 1000 × *g*. The supernatant containing the MuSCs was filtered using 100, 70, and 40 μm cell strainer (Greiner, EASYstrainer^TM^), centrifuged again briefly at 1000 × *g* and taken up in 200 μl cell sorting buffer (1x PBS, 1 mM EDTA, pH 8.0, 25 mM HEPES, pH 7.0, 1% FCS). The following antibodies were added 1:100 for 30 min at 4 °C prior to FACS sorting: anti-CD45-APC, anti-CD31-APC, anti-Ly-6A/E(Sca-1)-APC (eBioscience, #17-0451, #17-0311, #17-5981) and α-integrin FITC (MBL, #K0046-4). After a short wash, cells were incubated with anti-APC MicroBeads (Miltenyi, #130-090-855) for 20 min at 4 °C and the Milteny AutoMACS system was used to deplete CD31, CD45 and Sca-1 positive cells. The remaining cells were treated with DAPI (Invitrogen, #D1306) and underwent FACS (Aria III, BD FACS Diva v8 Software) sorting. 4 × 10^5^ Isolated muscle stem cells were seeded on Matrigel (Corning #CB356238) coated 24-well plates in DMEM high glucose GlutaMAX medium containing 20% FCS; 2% Penicillin/Streptomycin (PS) and 0.1% basic fibroblast growth factor (bFGF) for proliferation. Upon reaching 80% confluence, cells were transfected using Lipofectamine 3000 (ThermoFisher, #L3000075) according to the manufacturer’s recommendation in differentiation medium (DMEM high glucose GlutaMAX, 2% horse serum, 1% PS) for 48 h. U6 promoter-sgRNA expression cassettes were generated by PCR using a previously described strategy^44,59^, based on amplification of the U6 promoter from an appropriate plasmid vector (GTAAAACGACGGCCAGTGAGGGCCTATTTCCCATGATTC, GGTGTTTCGTCCTTTCCAC) and subsequent amplification of expression cassettes using the U6 promoter fragment, phosphorothioate-modified oligonucleotides for amplification (G*T*AAAACGACGGCCAGTGAGGGCCTATTTCCCATGATTC, A*G*GAAACAGCTATGACCATGAAAAAAAGCACCGACTCGGTGCCAC), an oligonucleotide providing the termination sequence (AAAAAAAGCACCGACTCGGTGCCACTTTTTCAAGTTGATAACGGACTAGCCTTATTTTAACTTGCTATTTCTAGCTCTAAAAC) and oligonucleotides providing guide RNA sequence (GTGGAAAGGACGAAACACCgGTGACATTCATGTGGCAGTGGTTTTAGAGCTAGAAATAG or GTGGAAAGGACGAAACACCgATTAACTCAAAATAGAATGGGTTTTAGAGCTAGAAATAG or GTGGAAAGGACGAAACACCgTTCTGTCCTAGGCAACCTTGGTTTTAGAGCTAGAAATAG). The expression cassettes were purified by agarose gel electrophoresis, pooled and transfected into dCas9-SPH^pos^: Pax7^pos^ MuSCs.

### hiPSC culture, transfection and differentiation

Somatic cells were obtained from a healthy, female donor with informed consent and upon approval of the Ethics committees of Technical University of Munich and the local regulatory board (Regierung von Oberbayern, Munich, Germany). Additional information about the donor, generation, and characterization of the line has been published previously^60,61^. Induced pluripotent stem cells were cultured as described in previous work^61^. In short, hiPSCs were maintained on Geltrex-coated plates in E8-flex medium (ThermoFisher Scientific, #A1413202, #A2858501) at 37 °C, 5% CO_2_. Upon passage with Versene (ThermoFisher Scientific, #15040066), cells were cultured in E8-flex Medium supplemented with 2 µM Thiazovivin (Merck, #420220) for 24 h.

To generate annealed oligos, the crRNA (AGGCATAGCCACATAGCAGG, IDT) and tracrRNA (IDT, #10007810, both 100 µM in Nuclease Free Duplex Buffer, IDT, #11040201), were mixed at a ratio of 1:1 and incubated at 95 °C for 5 min, then cooled to room temperature. The RNP complex was generated by mixing 3 µl of the annealed oligo and 2 µl Alt-R Cas9 Hifi Enzyme V3 (IDT, #1081060) and incubating for 50 min at RT. A single-cell solution of hiPSCs was prepared using Accutase solution (Sigma, #A6964) with 2 µM Thiazovivin (10 min, 37 °C), which was nucleofected using the P3 Primary Cell 4D-Nucleofector® X Kit (Lonza, #V4XP-3024). For this purpose, 800,000 single cells were resuspended in 100 µl Nucleofector Solution (18% Supplement in P3) and mixed with 120 pmol Alt-R Cas9 Electroporation Enhancer (IDT, #1075916) and 120 pmol HDR Donor Oligo (CTGAGACAGCCTTGGTCTGAGGGAGCAAAGACAGAGAATTCACGTGCTGCCCAGGCATAGGAATTCTGGTTACAAATAAAGCAATAGCATCACAAATTTCACAAATAAAGCATTTTTTTCACATCCCTGGACACTTTTCTGGTAAGCATAATCATTTTAGATAACAAAGTCCTGTACTAATACATAGTTGTTGAAATTGA) in IDTE-buffer (IDT, #11010202). Cells were mixed with the RNP complex, transferred to the cuvette, and nucleofected using an Amaxa 4D Nucleofector on the setting CA137. After incubation for 10 min at 37 °C, the cells were cultured for 12 h in E8-flex medium with 2 µM Thiazovivin and 1 µM HDR enhancer (IDT, #10007910), after which the cells were maintained in medium without supplement.

Edited hiPSCs were seeded in a serial dilution to generate single-cell clones and were propagated in E8-flex containing Thiazovivin until sufficiently large colonies had formed. Colonies were isolated, and mutated clones were identified and confirmed by PCR (TGTGTAACGTTGTAGACTGCTA, AAGAAAGTGTTGTCTTTGATCTTGT) followed by Sanger Sequencing of the PCR product (Eurofins Genomics). Differentiation of hiPSCs to hiPSC-CMs has been described extensively elsewhere^62^. In short, around 75% confluent hiPSCs were treated with differentiation medium (RPMI 1640 with HEPES with GlutaMax, 0.5 mg/ml Albumin, 0.2 mg/ml L-Ascorbic Acid 2-Phosphate) supplemented with a sequence of WNT modulators (4 µM CHIR99021 for 48 h, then 5 µM IWP2 for 48 h; Merck, #361559 and #681671).

Differentiated hiPSC-CMs were maintained in culture medium (RPMI 1640 with HEPES and GlutaMax, supplemented with 2% B27 Supplement; Thermo Scientific, #72400054 and #17504044) from day 8 onwards. Metabolic selection was carried out between days 15 and 20 for a duration of 5 days (RPMI 1640 without glucose, Thermo Scientific, #11879020; 0.5 mg/ml Albumin, Sigma, #A9731; 0.2 mg/ml L-Ascorbic Acid 2-Phosphate, Sigma, #A8960; 4 mM lactate, Sigma, #71718) before switching back to the original culture medium. Cells were dissociated using Collagenase II (Worthington Biochemical, 400 U/ml in RPMI) before treatment with 0.25% Trypsin-EDTA (ThermoFisher Scientific, #25200-056), and replated in a monolayer format. Cells recovered for 2 days in culture medium with 20% FCS (Sigma, #F7524) and 2 µM Thiazovivin before maturing in culture medium until at least day 60 of differentiation.

### Statistical analysis

All statistical analyses were performed using GraphPad Prism 10 Version 10.2.3. To assess data distribution, the Shapiro-Wilk normality test was performed. For data passing the normal distribution, an unpaired Student’s *t* test was used. In case of non-normal distribution, the non-parametric Mann–Whitney test was performed. For multiple comparisons and a single variable, one-way ANOVA with Fisher’s LSD test was employed. In case of several variables, two-way ANOVA and Šídák’s multiple comparisons test were used. Outliers were identified using the ROUT method implemented in GraphPad Prism (*Q* = 1%) and excluded from subsequent analyses.

### Reporting summary

Further information on research design is available in the Nature Portfolio Reporting Summary linked to this article.

## Supplementary information

Supplementary Information

Reporting Summary

Transparent Peer Review File

## Source data

Source Data 2

Source Data 1

## Data Availability

The following datasets generated in this study were submitted to NCBI GEO, Arrayexpress and jPOST: GSE301757 [https://www.ncbi.nlm.nih.gov/geo/query/acc.cgi?acc=GSE301757] (*Trdn-as* KO RNA-seq) GSE301758 (*Mettl3* KO RNA-seq) GSE301905 (RNA PolII ChIP-seq). E-MTAB-15342 (Transcriptome of cardiomyocytes/non-cardiomyocytes) JPST003939 (heart proteome, proteome of heart membrane preparations, TRDN IP) JPST004477 (TRDN IP). The following previously published ENCODE and GEO datasets were used in this study: GSE116250 [https://www.ncbi.nlm.nih.gov/geo/query/acc.cgi?acc=GSE116250] (human heart failure RNA-seq) GSE15998 (mouse multiple tissue) ENCSR247RPX (mouse developmental expression of triadin and *Trdn-as*) ENCFF305EHP (mouse heart RNA polymerase II RPB ChIP-seq) GSE142518 (mouse m. *tibialis anterior* RNA polymerase II RPB ChIP-seq) ENCSR451NAE (mouse heart ATAC-seq) ENCSR000AHH (human left ventricle RNA-seq) GSM2944730 (mouse heart SRF ChIP-seq) ENCSR777VNA (mouse heart EP300 ChIP-seq) GSM3067579 (mouse heart TBX5 ChIP-seq) GSM3067576 (mouse heart GATA4 ChIP-seq) GSM3518677 (mouse heart TEAD1 ChIP-seq) GSM1264378 (mouse heart H3K27ac,) ENCFF060VQA (mouse heart H3K4me3 ChIP-seq) GSE163491 (mouse heart miCLIP). Source data are provided with this paper.
