# Supplementary material for: Trdn-as directs m6A-dependent transcriptional termination for accurate triadin isoform switching, preventing aberrant dyads and cardiomyopathy
Source: Nat Commun. 2026 Jul 25;17:7269. doi: 10.1038/s41467-026-75985-8 (PMC13401598; doi:10.1038/s41467-026-75985-8)
Supplement: Supplementary file 1 — Supplementary Information [file 41467_2026_75985_MOESM1_ESM.pdf]

Supplementary Information

for

Trdn-as directs m6A-dependent transcriptional termination for accurate triadin isoform switching, preventing aberrant dyads and cardiomyopathy.

by Hofmann et al.

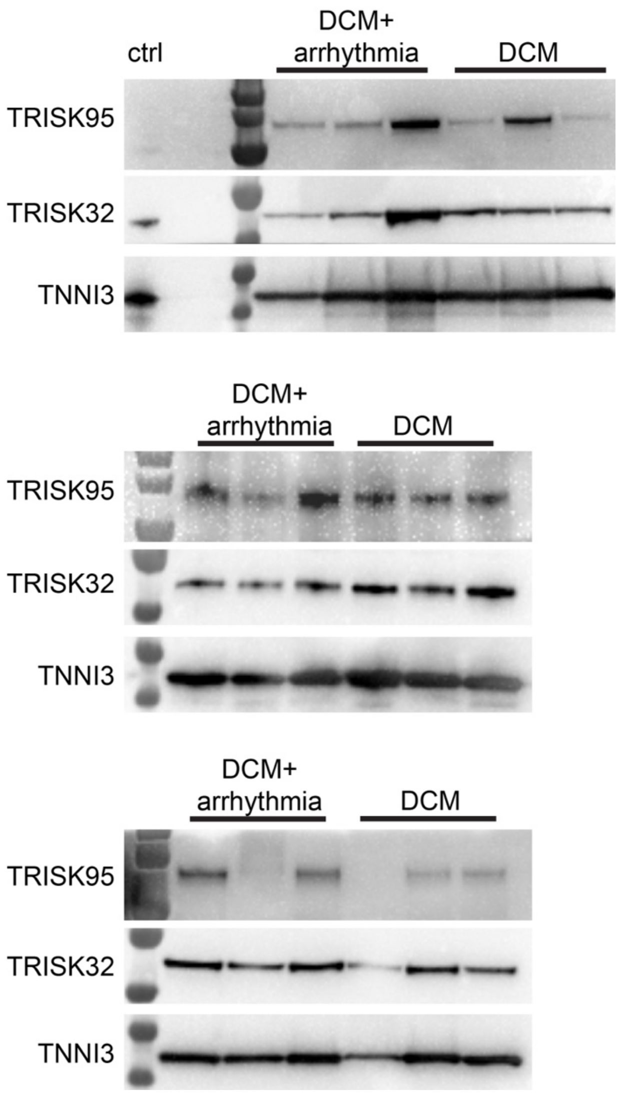

Supplementary Figure S1: Western blot analysis of myocardial samples derived from dilative cardiomyopathy patients.

Western blot analysis was performed on myocardial samples derived from patients with dilative cardiomyopathy with or without previous record of myocardial arrhythmia and probed for detection of TRISK95, TRISK32 and TNNI3. A control tissue lysate of non-failing human heart tissue is used in the upper blot (abcam #ab29431).

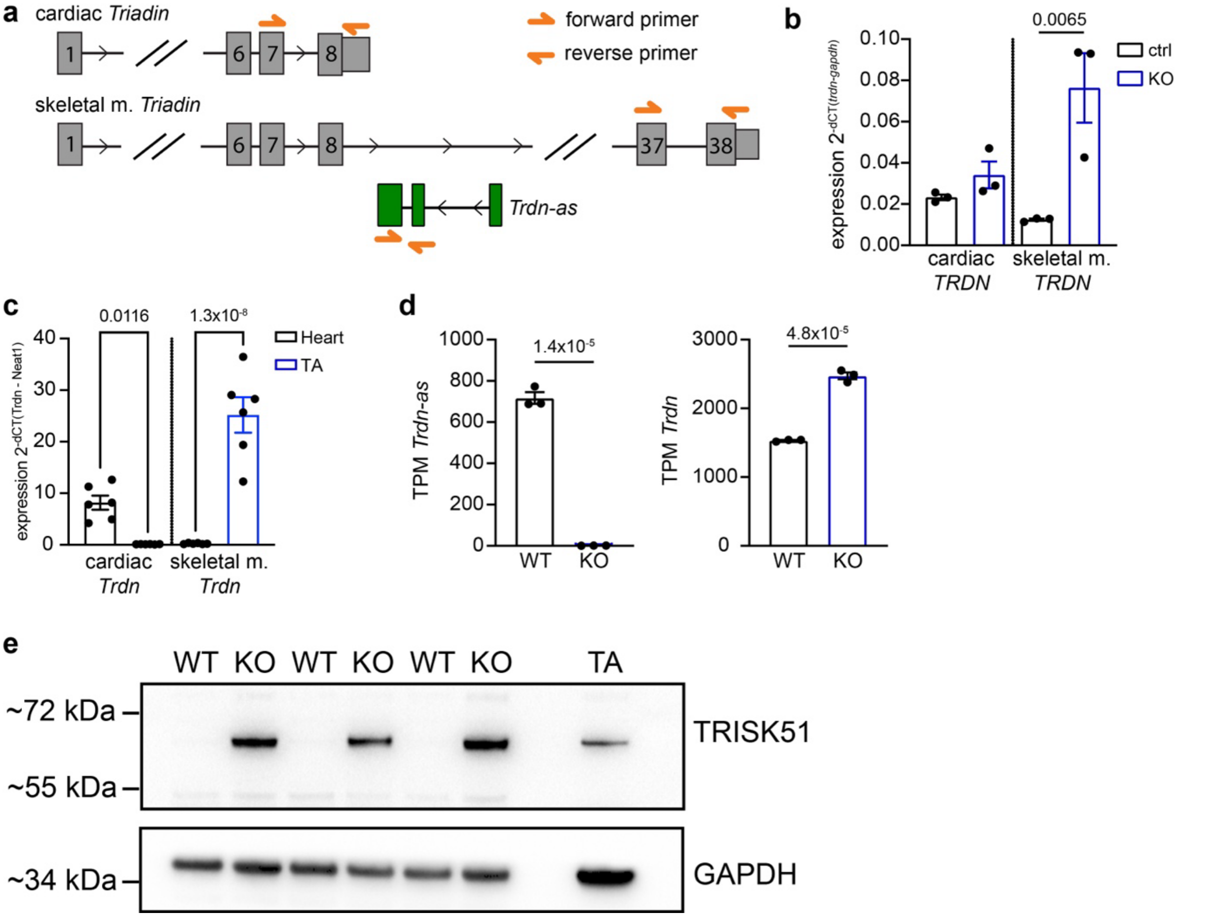

Supplementary Figure S2: Transcription of the cardiac-specific *Trdn* isoform is governed by *Trdn-as*.

a Schematic representation of the localization of *Trdn*-isoform specific RT-qPCR primer combinations. A reverse primer targeting the cardiac-specific extension of exon 8 was used to detect cardiac *Trdn* (coding for TRISK32), whereas skeletal muscle specific *Trdn* was detected using primers spanning exons 37 and 38.

b RT-qPCR of cardiac and skeletal muscle *TRDN* in control and *TRDN-AS* KO hiPSC-CMs (n = 3 ctrl clones, n = 3 *TRDN-AS* KO clones, Kruskal-Wallis test, Dunn's multiple comparisons test, mean ± SEM).

c Comparison of the absolute cardiac and skeletal muscle *Trdn* expression in WT heart and *M. tibialis anterior* (TA) using RT-qPCR (n = 6 WT hearts, n = 6 WT TAs, one-way ANOVA, Šídák's multiple comparisons test, mean ± SEM).

d RNA-seq analysis of WT and *Trdn-as* KO hearts confirms the loss of *Trdn-as* expression and increased expression of skeletal muscle-specific *Trdn* exons following polyA insertion (n = 3 WT hearts, n = 3 *Trdn-as* KO hearts, two-tailed unpaired Student's t-test, mean ± SEM).

e Western blot analysis of heart tissue lysates of WT and *Trdn-as* KO hearts reveals the expression of TRISK51 (Vassilopoulos, S. et al., JBC (2005) 280(31): 28601-9) in *Trdn-as* KO hearts. *M. tibialis*

anterior (TA) lysate serves as a control. The TRISK51 encoding transcript is derived from the skeletal muscle-specific *Trdn* transcript by intron retention following exon 21. In mice there is no evidence for TRISK49, previously detected in rat skeletal muscle.

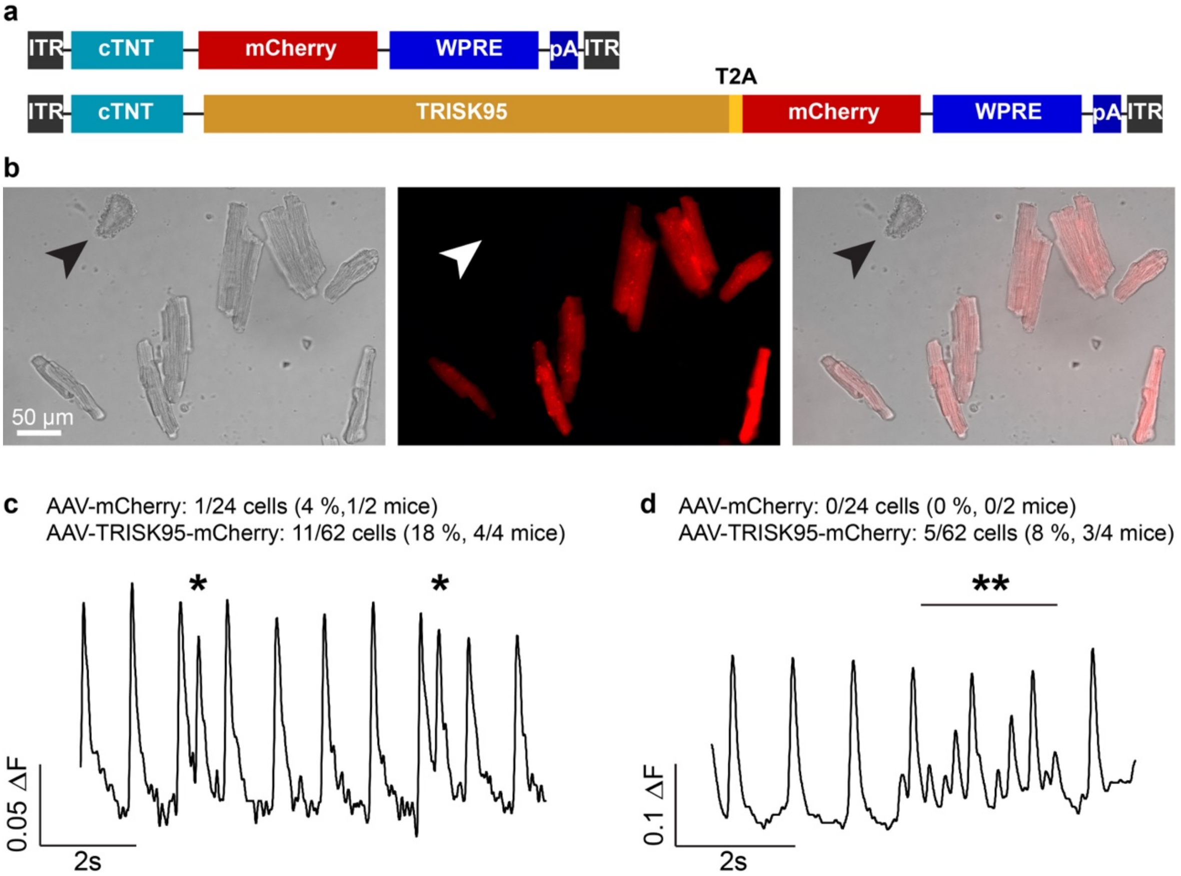

Supplementary Figure S3: AAV-driven ectopic expression of TRISK95 in cardio–myocytes.

a Schematic representation of AAVs used in this study.

b Expression of mCherry in isolated mouse cardiomyocytes used for calcium imaging. Arrowhead indicates a mCherry-negative non-cardiomyocyte (brightfield, mCherry, overlay images).

c Cardiomyocytes isolated from WT animals injected with AAV-TRISK95-mCherry reveal spontaneous calcium transients (tagged *), while this was rarely observed in AAV-mCherry controls (n = 2 ctrl animals, n = 4 TRISK95 animals).

d Cardiomyocytes isolated from WT animals injected with AAV-TRISK95-mCherry reveal spontaneous self-terminating calcium oscillations (tagged **), while this was not observed in AAV-mCherry controls (n = 2 ctrl, n = 4 TRISK95 animals).

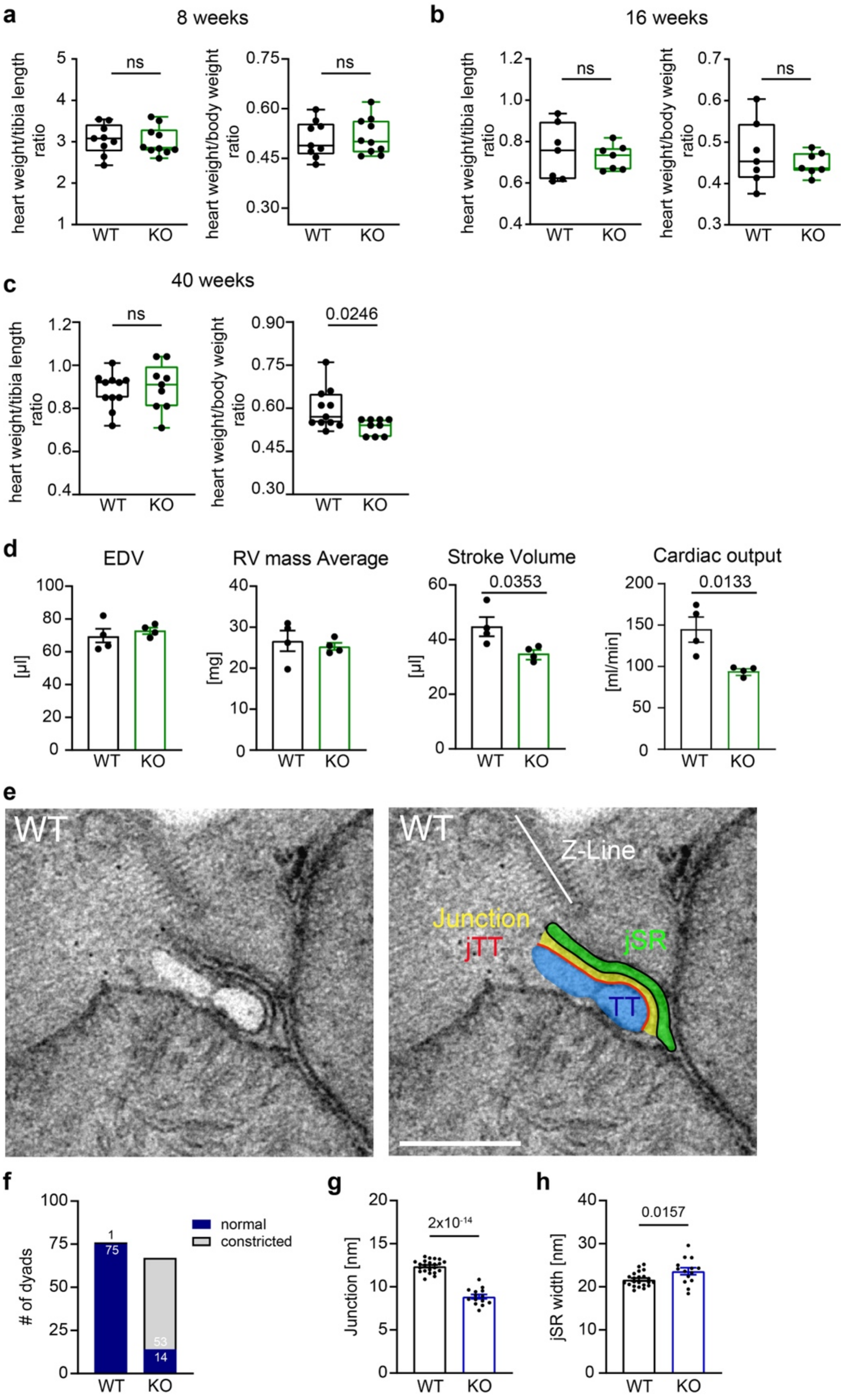

Supplementary Figure S4: *Trdn-as* is required for normal function of the heart.

a-**c** Decreased heart weight to body weight ratios in ageing *Trdn-as* KO mice (each filled circle represents one mouse (**a** n = 9 WT, n = 10 *Trdn-as* KO, **b** n = 7 WT, n = 7 *Trdn-as* KO. **c** n = 11 WT, n = 9 *Trdn-as* KO; Box, 25th–75th percentiles; whiskers, min – max all points shown, center: median; two-tailed unpaired Student’s t-tests).

d MRI measurements of 40-week old WT and *Trdn-as* KO mice, demonstrating reduced stroke volume and cardiac output, whereas end-diastolic volume (EDV) and right ventricular mass (RV mass) are not altered (n = 4 WT mice, n = 4 *Trdn-as* KO mice, two-tailed unpaired Student's t-test, mean ± SEM).
e Quantitative analysis of T-tubule/junctional SR coupling by transmission electron microscopy. Representative transmission electron microscopy image of a WT dyad showing a T-tubule (TT, blue) apposed to the junctional sarcoplasmic reticulum (jSR, green). Junctional cleft demonstrating the gap between TT and jSR (Junction, yellow) and the length of the T-tubule membrane in close proximity to the junctional cleft (jTT, red). The Z-line is indicated by a white line. The scale bar corresponds to 250 nm.
f-h Quantification of dyad parameters revealing the number of constricted or collapsed dyads (**f**; n = 76 WT dyads, n = 67 *Trdn-as* KO dyads analyzed), and the Junction and jSR width (**g**, **h**) comparing 3 WT and 3 *Trdn-as* KO hearts (n = 22 WT dyads, n = 14 *Trdn-as* KO dyads, collapsed dyads excluded from analysis; two-tailed unpaired Student's t-test, mean ± SEM).

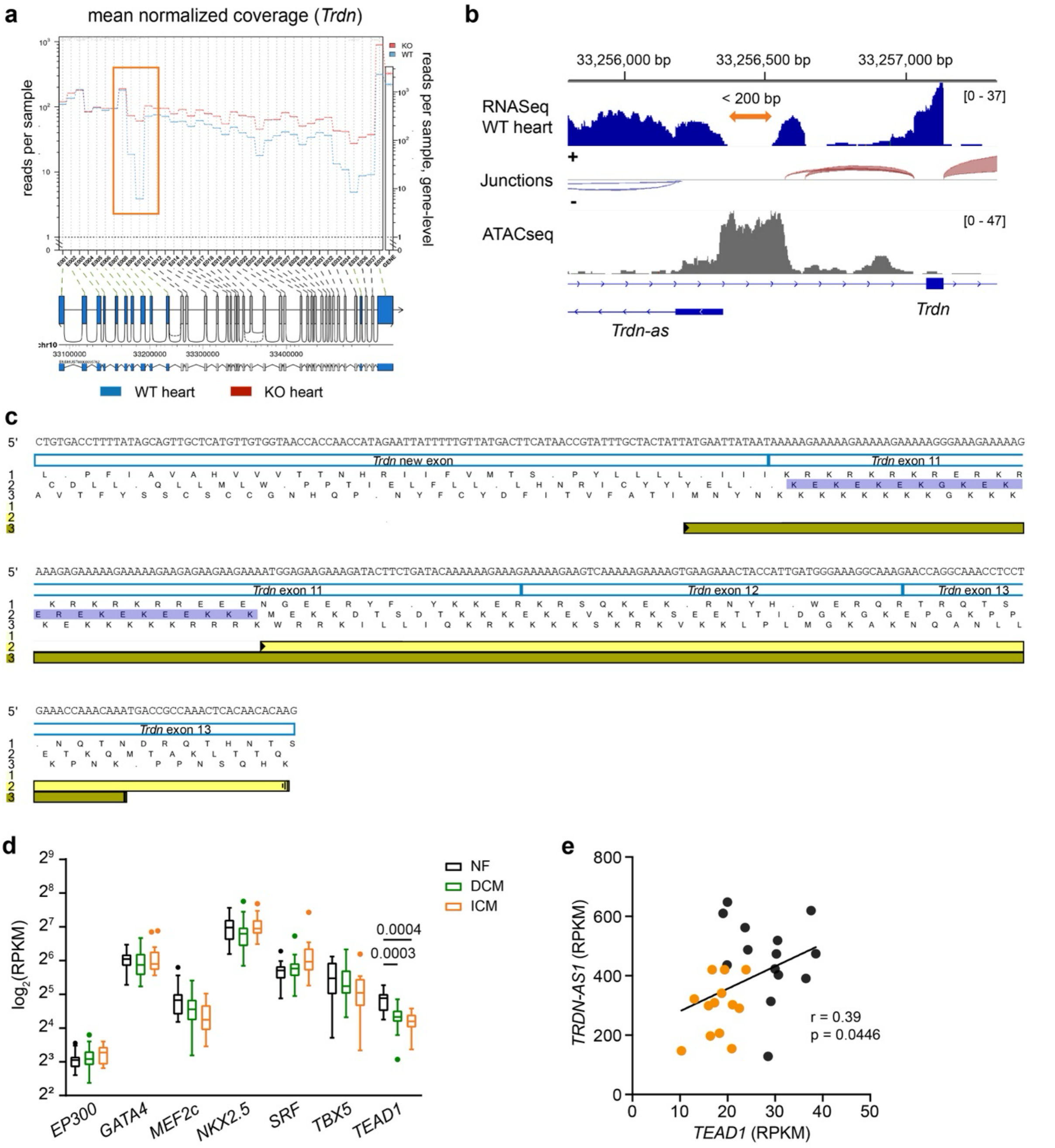

Supplementary Figure S5: Expression of *Trdn-as* is directed by a bidirectionally active promoter.

a JunctionSeq analysis of the *triadin* and *Trdn-as* locus illustrating the mean normalized exon coverage in WT and *Trdn-as* KO hearts. Loss of *Trdn-as* transcription increases expression and splicing over exon 8 – 10 (orange square), exons 11-38 remained virtually unchanged.

b Genome browser view of the *Trdn-as* promoter region showing chromatin accessibility (ATACseq, #ENCSR451NAE) and RNA-seq signals in WT hearts. Expression of a heart-specific non-annotated exon upstream of the *Trdn-as* promoter, which is spliced to the skeletal muscle specific *triadin* exon

11. The close proximity (< 200 bp) between the newly identified exon and the first *Trdn-as* exon indicates a possible bi-directionality of the *Trdn-as* promoter.

c Predicted open reading frames (ORFs) at the sense transcript driven by the *Trdn-as* promoter. A potential ORF starts in the newly identified exon in frame 3, resulting in a frame shift and early translational stop, whereas frame 2 resembles truncated skeletal muscle TRDN harboring the characteristic 'KEKE' motif but starts beyond the potential ORFs in frame 3.

d Expression of candidate transcription factors potentially regulating *TRDN-AS* in RNA-seq data from human heart failure patients (#GSE116250). Patients diagnosed with dilated cardiomyopathy (DCM) and ischemic cardiomyopathy (ICM) show significant downregulation of *TEAD1* expression (NF = 14, DCM = 37, ICM = 13; multiple Mann-Whitney, Holm-Šidák method. Tukey boxplot.).

e Correlation between *TEAD1* and *TRDN-AS1* expression in RNA-seq data of human heart failure patients (GSE116250). Significant correlation between *TEAD1* and *TRDN-AS1* expression was detected in ICM but not in DCM patients (NF = 14, ICM = 13; Pearson correlation analysis, two-tailed).

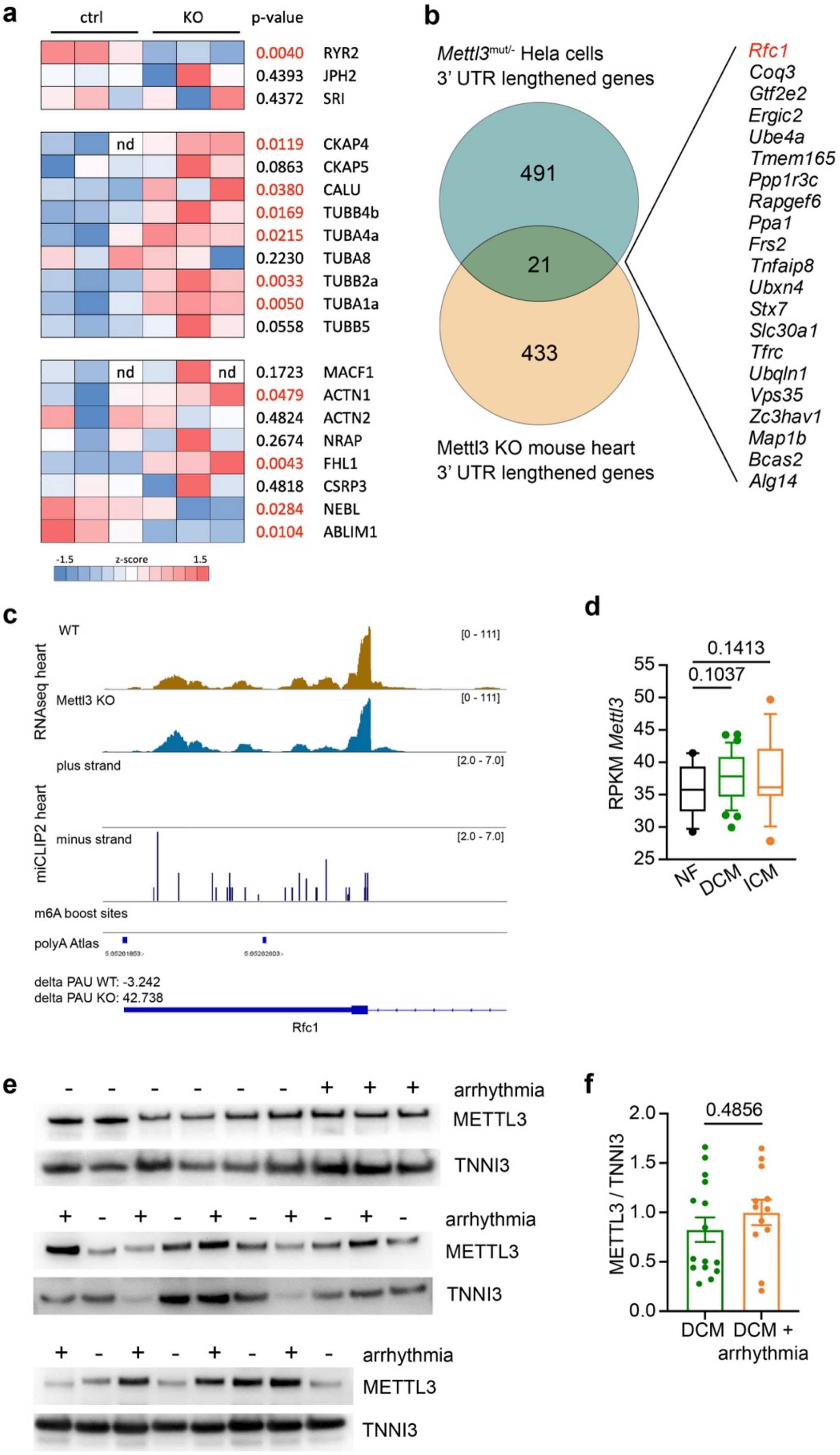

Supplementary Figure S6: METTL3 is involved in the selection of polyA sites.

a TRDN-IP using WT and *Mettl3* KO heart lysates. Proteins detected in this experiment and highlighted for the TRDN-IP using WT and *Trdn-as* KO mice (Fig. 5c, f, h) are shown (z-score of LFQ data, one-

tailed unpaired Student's t-test on LFQ intensities, n = 3 ctrl hearts, n = 3 αMHC-MerCreMer x *Mettl3*$^{lox/lox}$ hearts).

b Comparison of differentially polyadenylated genes after inactivation of *Mettl3* in human HeLa cells and mouse hearts. Based on data generated in this study and by Yue Y, et al. Cell Discov. (2018) 4:10.

c Representative genome browser view of *Rfc1* identified in **b**, with RNA-seq expression data of WT and *Mettl3* KO mice, delta PAU, miCLIP2 data and polyA sites similar to Fig. 8b.

d Analysis of *METTL3* expression in samples from human non-failing hearts (NF), dilated cardiomyopathy (DCM), and ischemic cardiomyopathy (ICM). Data are from GSE116250 (NF = 14, DCM = 37, ICM = 13; one-way ANOVA, uncorrected fishers LSD test, Box: 25th–75th percentiles, whiskers: 10th–90th percentile, center: median).

e Western blot analysis of METTL3 and TNNI3 in human heart samples from patients with DCM (-) or DCM with documented arrhythmia (+).

f Statistical evaluation of METTL3 and TNNI3 Western blot data. The ratio METTL3 / TNNI3 was normalized to the DCM-arrhythmia (+) samples of the respective blots (two-tailed Mann-Whitney test, mean ± SEM).

Supplementary Table 1: Oligonucleotides used in this study for RT-qPCR.

Gene info	Sequence 5’ > 3’	Species
Trdn-as	GTTGTAGTATGTCTGAATGCTATGC	mouse
	GTCCAAGCTTATAGCTAGGCAGCAC	
Cardiac triadin	GAGATGCCAGCTGTGCATGAGCAG	
	CAGGCTTGTTGGCTTGGATCTC	
Skeletal muscle triadin	GCCACCACCATCATTGACACCTTCC	
	GCCTGGTTCTTTGCCTTTCCCATC	
Trdn-as DS 1	GTATCTCCAGCGTTGCCTCACTTTG	
	CAGGAAAATATAACGAAAGAGATAG	
Trdn-as DS 2	CGGACCCATGGCTCCAGCCACATATG	
	CATCCTGATTCTATGAGGGTGTTCAG	
Neat1	AGGAGGCCATCGTTGAAGTCAGCTT	
	CCATTCATGCATCCGCAAAGAATGA	
Gapdh	ACCACAGTCCATGCCATCAC	
	CATGCCAGTGAGCTTCCCGT	
Mettl3	CCTCCGATGTTGATCTGGAGATAG	
	GGTCCCATAATCACAAAATTCTTGC	
TRDN-AS	CCCCAATCCTGCAAACAATGAATG	human
	CTGTGTTTTCCATTCTCTGCC	
Cardiac triadin	GTACAGAAAACACCATCAAAACCC	
	CCCCCATTTGAAGTCTGATTTTGG	
Skeletal muscle triadin	CCAAGTGACAAACAAGTAAAAGC	
	CTTTCAGAATTGAAGAAGTCTTCCC	
GAPDH	GTCTCCTCTGACTTCAACAGCG	
	ACCACCCTGTTGCTGTAGCCAA	

Supplementary Table 2: *Trdn-as* FiSH probes.

Probes	Sequence
AS-T_1	GCATAGCATTCAGACATACT
AS-T_2	CCAGGAAACTTCATGGAACC
AS-T_3	GGCATGCTCAGATATCTGAA
AS-T_4	TATCTTTCTTGGCTCTAGTC
AS-T_5	TTATAGCTAGGCAGCACTTG
AS-T_6	AAAAAATGCCCATGGAGTCC
AS-T_7	TAGAGACACACCGGTACATC
AS-T_8	TTTCCTCGGCACATGAGAAA
AS-T_9	GTCTTTGAGATGCCTGTAAC
AS-T_10	TGTCCAAGAAGCTCTTGTTG
AS-T_11	CTGAAGATGAAGCCCCGAAG
AS-T_12	TGAAGTGAACTCTCCTTTGG
AS-T_13	TACAAGATGGTGTTCTCACC
AS-T_14	GTGAACTGGTCTCTGAGAGA
AS-T_15	CTCTTGCAGCAAAGGTACAC
AS-T_16	TGTCTGGCTAGAGCTTATTT
AS-T_17	GTGGTCTTGGTTTCTGTTAA
AS-T_18	TCACTGTGGTGGAAGTACTG
AS-T_19	ATCTGAATCTTGCTTTTCCG
AS-T_20	TTCAAGGACTTCTACCTGTT
AS-T_21	TGAGGGATTTTTATGCTTCC
AS-T_22	GTGTATGCTCTTTAAGGACT
AS-T_23	CCACACAATTGGGTTGGAAC
AS-T_24	CGGATGTCTGGAGCTTGAAA
AS-T_25	CTCCTTTTGCAGGGAAGATG
AS-T_26	AGACTAGTCTTGGAGGGATC
AS-T_27	AGAAGCTGTGTTGCTTTGAC
AS-T_28	GCAGAAGATGAAGGCCTGAA
AS-T_29	CAGCAGACCTGGGAAACTAG
AS-T_30	GTTGTCTCTGGTTAGAGATT

Supplementary DNA-Sequence Data

renilla::gctagccTGAGGCTTGGCTTCTGGGTTCCTCATAGAGACACACCGGTACATCCATT
TATACCTAGACACTAAGTAAAGAAATAATGCTGGAAGTCTTgaattCAGGCCAATTTCTAC
CACCCCAAAGAAACAATGTGTCAGTGAAG**TGGAATGCTGGAATGTGGTGTCA**TGGAAC
AACTGGGACCTGATTTAACTGGCAACAAAAATACACCAACACTGGCCCCATAGAAATGA
AAAGTATTCCTCTGCTATGTAGAGTTAGGATAAGCTCCATAGTACGAAGACTTGGAGAC
AACAAAACAAAAGGCCCAATTAGACCTACgaattcTTGCATTTGTTTCTTAGTTTTGTTGGA
AACAGAGTTTTAGAAAGCCTTACAACTATAATTAACTGTTGTGTGCTTTCTGGTTTTGAG
AAGTCATATAAACATTTAAAGGGAAGAGAAAAGCACAGAGTAAGGACTTAGGAAGTAAA
AATTAAGACTTCTCATTTTTCTTTCATTGATATTATTTTATGCAGAAGCTAATAACATTTTT
CTTGGGTCAGAGCTTTAGGTTTTAGCCAAGATTCCTTCATGAAAAAGCCATCATTTTTGG
GCTTTGTACAATTTTGTCAGAGAAGGACAATGTGCTGATAAGTCCAGCTGCTTCACACG
TGTTTAATTGCTAACTGACTGAATCCAATATCATTAAAGGCTATAACCCTTCTCCAAGTG
TTGCAAACAAAACAGCAAGCACATTAGCAGAC**AAGGCTGCTGAAATGTCTAAGTG**AAT
GCACAGTCACTTggaagacttggAGACTTACCAGAAAAAAATGCCCATGGAGTCCAAGCTTA
TAGCTAGGCAGCACTTGACTATCTTTCTTGGCTCTAGTCATACCATGGCATGCTCAGAT
ATCTGAAAGAGGAATCGTCCAGGAAACTTCATGGAACCATACATGATTTTGCATAGCAT
TCAGACATACTACAACCTTGGTGTACCCAAGAACTCTACTTTATAAAACTGTTCAGAAAG
GGTATTGA**AGGTAACATTCCTGCCACACT**GCCACATGAATGTCACCAGGTCCAAATATC
TGTTGACAAGAGCCAGAGATAAAAAAGAAAAAAAATTAACTCAAAATAGAATGGTGGAG
CACAGTAGTTCTTAACCTTGCTAGTGCTGTGACCTTTTATAGCAGTTGCTCATGTTGTGG
TAACCACCAACCATAGAATTATTTTTGgcctcgag::firefly

DNA sequence used in the reporter vector containing promoter and enhancer sequences indicating TEAD1 ChIP peaks (grey background), DNA binding motifs: TEAD1, GATA4, SRF, TBX5, **deletions (bold)** introduced into the reporter vectors to demonstrate specificity of TEAD1 activation and linker sequences (lower case).
