# Supplementary material for: Trdn-as directs m6A-dependent transcriptional termination for accurate triadin isoform switching, preventing aberrant dyads and cardiomyopathy
Source: Nat Commun. 2026 Jul 25;17:7269. doi: 10.1038/s41467-026-75985-8 (PMC13401598; doi:10.1038/s41467-026-75985-8)
Supplement: Supplementary file 2 — Reporting Summary [file 41467_2026_75985_MOESM2_ESM.pdf]

nature portfolio

Corresponding author(s): Thomas Braun
Thomas Boettger

Last updated by author(s): Mar 12, 2026

Reporting Summary

Nature Portfolio wishes to improve the reproducibility of the work that we publish. This form provides structure for consistency and transparency in reporting. For further information on Nature Portfolio policies, see our Editorial Policies and the Editorial Policy Checklist.

Statistics

For all statistical analyses, confirm that the following items are present in the figure legend, table legend, main text, or Methods section.

n/a	Confirmed	
☐	☒	The exact sample size (*n*) for each experimental group/condition, given as a discrete number and unit of measurement
☐	☒	A statement on whether measurements were taken from distinct samples or whether the same sample was measured repeatedly
☐	☒	The statistical test(s) used AND whether they are one- or two-sided<br>*Only common tests should be described solely by name; describe more complex techniques in the Methods section.*
☒	☐	A description of all covariates tested
☐	☒	A description of any assumptions or corrections, such as tests of normality and adjustment for multiple comparisons
☐	☒	A full description of the statistical parameters including central tendency (e.g. means) or other basic estimates (e.g. regression coefficient) AND variation (e.g. standard deviation) or associated estimates of uncertainty (e.g. confidence intervals)
☐	☒	For null hypothesis testing, the test statistic (e.g. *F*, *t*, *r*) with confidence intervals, effect sizes, degrees of freedom and *P* value noted<br>*Give P values as exact values whenever suitable.*
☒	☐	For Bayesian analysis, information on the choice of priors and Markov chain Monte Carlo settings
☒	☐	For hierarchical and complex designs, identification of the appropriate level for tests and full reporting of outcomes
☒	☐	Estimates of effect sizes (e.g. Cohen's *d*, Pearson's *r*), indicating how they were calculated

Our web collection on statistics for biologists contains articles on many of the points above.

Software and code

Policy information about availability of computer code

Data collection	ZEISS ZEN 3, Dataquest A.R.T 4, ImageLab 5, StepOne 2, IonWizard 6, MicroWin 2000
Data analysis	GraphPad Prism 10, IGV 2.16, GSEA 4, Labchart 7Pro, Qmass Mass4Mice, Lasergene 17, Affy TAC 4, Image J, Maxquant, FragPipe, Trimmomatic 0.39, STAR 2.6.1d /2.7.11, Picard 2.21.1 / 3.1.1, DESeq2 version 1.18.1/ 1.36.0, Macs 3, QAPA software 1.3.3, salmon 1.10.0

For manuscripts utilizing custom algorithms or software that are central to the research but not yet described in published literature, software must be made available to editors and reviewers. We strongly encourage code deposition in a community repository (e.g. GitHub). See the Nature Portfolio guidelines for submitting code & software for further information.

Data

Policy information about availability of data

All manuscripts must include a data availability statement. This statement should provide the following information, where applicable:

- Accession codes, unique identifiers, or web links for publicly available datasets
- A description of any restrictions on data availability
- For clinical datasets or third party data, please ensure that the statement adheres to our policy

The following datasets generated in this study were submitted to NCBI GEO, Arrayexpress and jPOST:
GSE301757 [https://www.ncbi.nlm.nih.gov/geo/query/acc.cgi?acc=GSE301757] (Trdn-as KO RNA-seq)
GSE301758 [https://www.ncbi.nlm.nih.gov/geo/query/acc.cgi?acc=GSE301758] (Mettl3 KO RNA-seq)

GSE301905 [https://www.ncbi.nlm.nih.gov/geo/query/acc.cgi?acc=GSE301905] (RNA PolII ChIP-seq).
E-MTAB-15342 [https://www.ebi.ac.uk/biostudies/ArrayExpress/studies/E-MTAB-15342] (Transcriptome of cardiomyocytes / non-cardiomyocytes)
JPST003939 [https://repository.jpostdb.org/entry/JPST003939] (heart proteome, proteome of heart membrane preparations, TRDN IP)
JPST004477 [https://repository.jpostdb.org/entry/JPST004477] (TRDN IP).
The following previously published ENCODE and GEO datasets were used in this study:
GSE116250 [https://www.ncbi.nlm.nih.gov/geo/query/acc.cgi?acc=GSE116250] (human heart failure RNA-seq)
GSE15998 [https://www.ncbi.nlm.nih.gov/geo/query/acc.cgi?acc=GSE15998] (mouse multiple tissue)
ENCSR247RPX [https://www.encodeproject.org/organism-development-series/ENCSR247RPX/] (mouse developmental expression of triadin and Trdn-as)
ENCFF305EHP [https://www.encodeproject.org/search/?searchTerm=ENCFF305EHP] (mouse heart RNA polymerase II RPB ChIP-seq)
GSE142518 [https://www.ncbi.nlm.nih.gov/geo/query/acc.cgi?acc=GSE142518] (mouse tibialis anterior RNA polymerase II RPB ChIP-seq)
ENCSR451NAE [https://www.encodeproject.org/search/?searchTerm=ENCSR451NAE] (mouse heart ATACseq)
ENCSR000AHH [https://www.encodeproject.org/search/?searchTerm=ENCSR000AHH] (human left ventricle RNA-seq)
GSM2944730 [https://www.ncbi.nlm.nih.gov/geo/query/acc.cgi?acc=GSM2944730] (mouse heart SRF ChIP-seq)
ENCSR777VNA [https://www.encodeproject.org/experiments/ENCSR777VNA/] (mouse heart EP300 ChIP-seq)
GSM3067579 [https://www.ncbi.nlm.nih.gov/geo/query/acc.cgi?acc=GSM3067579] (mouse heart TBX5 ChIP-seq)
GSM3067576 [https://www.ncbi.nlm.nih.gov/geo/query/acc.cgi?acc=GSM3067576] (mouse heart GATA4 ChIP-seq)
GSM3518677 [https://www.ncbi.nlm.nih.gov/geo/query/acc.cgi?acc=GSM3518677] (mouse heart TEAD1 ChIP-seq)
GSM1264378 [https://www.ncbi.nlm.nih.gov/geo/query/acc.cgi?acc=GSM1264378] (mouse heart H3K27ac,)
ENCFF060VQA [https://www.encodeproject.org/search/?searchTerm=ENCFF060VQA] (mouse heart H3K4me3 ChIP-seq)
GSE163491 [https://www.ncbi.nlm.nih.gov/geo/query/acc.cgi?acc=GSE163491] (mouse heart miCLIP).
Source data are provided with this paper.

Research involving human participants, their data, or biological material

Policy information about studies with human participants or human data. See also policy information about sex, gender (identity/presentation), and sexual orientation and race, ethnicity and racism.

Reporting on sex and gender
Sex or gender were not considered in this study. Information on sex or gender were not collected.

Reporting on race, ethnicity, or other socially relevant groupings
Socially constructed or socially relevant categorization variables were not collected and were not used in this study.

Population characteristics
Details on potential covariate-relevant population characteristics (age, genotypic information, treatment) were not considered in this study due to the limited number of patients who were included in the study.

Recruitment
Patients with non-ischemic dilated cardiomyopathy were enrolled in the subproject TP9a of the German Competence Network Heart Failure from 2004 to 2008. All patients underwent a comprehensive evaluation, including a detailed medical history, clinical examination, laboratory testing, and echocardiographic assessment. Coronary angiography was performed in all cases to exclude an ischemic etiology of cardiac dysfunction. Additionally, patients with systemic inflammatory or autoimmune diseases, sarcoidosis, or amyloidosis were excluded 39. From this study cohort, patients with available endomyocardial biopsies (EMB) and a history of sustained ventricular tachycardia, sustained supraventricular tachycardia, and those without a history of arrhythmia were identified and included for further analysis. EMBs were obtained from the left ventricle as described 39. The classification of arrhythmias was carried out by experienced clinicians (specialist training in cardiology, subspecialization in rhythmology), according to the corresponding guidelines of the European Society of Cardiology 40, 41. The study protocol was approved by the German Competence Network Heart Failure and conducted in accordance with the Declaration of Helsinki (1996), the International Conference on Harmonization Good Clinical Practice (ICH-GCP), and the Ethics Committee of the University Hospital of Marburg.
Additional myocardial samples were obtained from the Heart and Diabetes Centre NRW Bad Oeynhausen. All patients had a confirmed clinical diagnosis of non-ischemic dilated cardiomyopathy (DCM) and were categorized according to presence or absence of previously recorded arrhythmia. The study conformed with the rules of the Helsinki declaration. The study was approved by the local Ethics Committee of the Ruhr-University Bochum situated in Bad Oeynhausen, Germany (Reg. No. 2025-1371).
All patients included in the study provided written informed consent and received no compensation. The study protocols exclude a self-selection bias.

Ethics oversight
The study was approved by the German Competence Network Heart Failure and the ethics committee of the University Hospital of Marburg and the ethics committee of the Ruhr-University Bochum situated in Bad Oeynhausen, Germany

Note that full information on the approval of the study protocol must also be provided in the manuscript.

Field-specific reporting

Please select the one below that is the best fit for your research. If you are not sure, read the appropriate sections before making your selection.

☒ Life sciences ☐ Behavioural & social sciences ☐ Ecological, evolutionary & environmental sciences

For a reference copy of the document with all sections, see nature.com/documents/nr-reporting-summary-flat.pdf

Life sciences study design

All studies must disclose on these points even when the disclosure is negative.

Sample size	No sample size calculation was performed, sample size were chosen based on empirical considerations or based on the available material.
Data exclusions	Data were excluded from analysis using unbiased outlier identification methods included in GraphPad Prism 10 (ROUT method).
Replication	Biological and technical replicates were used to verify the reproducibility of experimental findings. All attempts at replication were successful.
Randomization	Samples were randomly allocated to experimental groups to exclude any experimental bias in the specific experiments.
Blinding	Investigator were blinded to group allocation during data collection and analysis for: EM data, histological data, ECG data, calcium transients, MRI data and all other experiments were applicable by revealing identity of samples only after the respective assay.

Reporting for specific materials, systems and methods

We require information from authors about some types of materials, experimental systems and methods used in many studies. Here, indicate whether each material, system or method listed is relevant to your study. If you are not sure if a list item applies to your research, read the appropriate section before selecting a response.

Materials & experimental systems

n/a	Involved in the study	
☐	☒	Antibodies
☐	☒	Eukaryotic cell lines
☒	☐	Palaeontology and archaeology
☐	☒	Animals and other organisms
☒	☐	Clinical data
☒	☐	Dual use research of concern
☒	☐	Plants

Methods

n/a	Involved in the study	
☐	☒	ChIP-seq
☒	☐	Flow cytometry
☒	☐	MRI-based neuroimaging

Antibodies

Antibodies used	anti-TRISK32 (gifted by Dr. I. Marty, 1:10000, Vassilopoulos S, et al. J Biol Chem 280, 28601-28609 (2005))<br>anti-TRISK51 (gifted by Dr. I. Marty, 1:10000, Vassilopoulos S, et al. J Biol Chem 280, 28601-28609 (2005))<br>anti-TRDN mouse and human (Abcam, #ab247008, 1:500)<br>anti-human TRDN (LifeSpan BioSciences, #LS-C749822, 1:500)<br>anti-Mettl3 (Abcam, #ab195352, 1:1000)<br>anti-GAPDH (Cell Signaling Technology, #2118, 1:2000)<br>anti-cTNNI3 (Abcam, #ab56357, 1:1000)<br>anti-RalA (Bd, #610221, 1:5000)<br>anti-mouse HRP (Pierce #1858413, 1:10000)<br>anti-rabbit HRP (Pierce #31460, 1:10000)<br>anti-rabbit IgG Trueblot HRP (Ebioscience, #18-8816-31, 1:5000)
Validation	Antibodies were validated using existing supplier documentation and literature, based on size, sensitivity and specificity of the signals at the Western blots. Alternative antibodies were used to confirm the results. Control tissues with known absence or presence of the respective protein were used (skeletal muscle tissue or extracts of differentiated HSMM cells for TRISK isoforms; Mettl3 KO tissue for Mettl3). Secondary antibodies were validated using blots without primary antibody.

Eukaryotic cell lines

Policy information about cell lines and Sex and Gender in Research

Cell line source(s)	HEK239 (ATCC CRL-1573); hiPSC are described: Gramlich, M. et al. EMBO Mol Med 7, 562-576 (2015); Esfandyari, D. et al. Nat Commun 13, 220 (2022).
Authentication	All cell lines used in this study were authenticated by the provider. Morphology of the cells was continuously confirmed according to the information provided by the culture collection.
Mycoplasma contamination	Cell lines were tested negative for mycoplasma contamination twice annually.
Commonly misidentified lines (See ICLAC register)	No commonly misidentified cell line were used.

Animals and other research organisms

Policy information about studies involving animals; ARRIVE guidelines recommended for reporting animal research, and Sex and Gender in Research

Laboratory animals	- Mus musculus, C57Bl6 (Charles River)<br>- Trdn-as KO and WT (this study).<br>- CAG-LSL-dCas9-SunTag-p65-HSF1pos: Pax7pos mice (Zhou H, et al. Nat Neurosci 21, 440-446 (2018); Keller C, et al. Genes Dev 18, 2608-2613 (2004); Klockner I, et al. Nat Commun 13, 3180 (2022)).<br>- Trdn-as DS2 pos. and neg. (this study).<br>- Mettl3 lox/lox x MerCreMer pos. and Mettl3 lox/lox x MerCreMer neg. (Geula S, et al. Science 347, 1002-1006 (2015), Sohal DS, et al. Circ Res 89, 20-25 (2001)).
Wild animals	The study did not involve wild animals.
Reporting on sex	Sex of animals was not considered in study design.
Field-collected samples	The study did not involve samples collected from the field.
Ethics oversight	All animal experiments were conducted in compliance with national and european community guidelines and received approval from the Committee for Animal Protection of the State of Hessen (Regierungspraesidium Darmstadt), Germany.

Note that full information on the approval of the study protocol must also be provided in the manuscript.

Plants

Seed stocks	n/a
Novel plant genotypes	n/a
Authentication	n/a

ChIP-seq

Data deposition

☒ Confirm that both raw and final processed data have been deposited in a public database such as GEO.

☐ Confirm that you have deposited or provided access to graph files (e.g. BED files) for the called peaks.

Data access links<br>*May remain private before publication.*	https://www.ncbi.nlm.nih.gov/geo/query/acc.cgi?acc=GSE301905 (secure token shyhycmetpgjxyj)
Files in database submission	GSM9092115: Polr2a-WT_1<br>GSM9092116: Polr2a-WT_2<br>GSM9092117: Polr2a-ko-Mettl3_1<br>GSM9092118: Polr2a-ko-Mettl3_2<br>GSM9092119: Polr2a-ko-Trdn_1<br>GSM9092120: Polr2a-ko-Trdn_2<br>GSM9092121: IgG-WT_1<br>GSM9092122: IgG-ko-Mettl3_1<br>GSM9092123: IgG-ko-Trdn_1<br>GSM9092124: WT_1_input<br>GSM9092125: ko-Mettl3_1_input<br>GSM9092126: ko-Trdn_1_input<br>GSM9092109: Ctcf-ko-Mettl3_1<br>GSM9092110: Ctcf-ko-Mettl3_2<br>GSM9092111: Ctcf-ko-Trdn_1<br>GSM9092112: Ctcf-ko-Trdn_2<br>GSM9092113: Ctcf-WT_1<br>GSM9092114: Ctcf-WT_2
Genome browser session<br>(e.g. UCSC)	n/a

Methodology

Replicates

ChIP n=2 replicates, Input n=1 replicate, IgG n=1

Sequencing depth

single ended, 72 bp read length,
id, reads processed, uniquely mapped reads
experiment, reads processed, uniquely mapped reads
Polr2a-WT_1, 26,837,604, 8,693,921
Polr2a-WT_2, 25,166,147, 11,694,116
Polr2a-ko-Mettl3_1, 37,526,580, 10,878,672
Polr2a-ko-Mettl3_2, 36,248,034, 7,540,239
Polr2a-ko-Trdn_1, 42,371,581, 12,080,914
Polr2a-ko-Trdn_2, 39,493,961, 10,682,858
IgG-WT_1, 46,465,992, 16,547,284
IgG-ko-Mettl3_1, 37,296,245, 7,526,834
IgG-ko-Trdn_1, 30,457,811, 5,268,859
WT_1_input, 57,096,279, 36,109,340
ko-Mettl3_1_input, 70,837,656, 46,265,217
ko-Trdn_1_input, 63,749,682, 41,550,839
Ctcf-ko-Mettl3_1, 34,002,201, 21,979,692
Ctcf-ko-Mettl3_2, 43,640,813, 26,462,725
Ctcf-ko-Trdn_1, 40,052,812, 24,069,321
Ctcf-ko-Trdn_2, 31,052,348, 17,995,900
Ctcf-WT_1, 34,680,194, 20,258,742
Ctcf-WT_2, 45,535,880, 28,526,650

Antibodies

anti-RNA Pol II antibody (Diagenode, #C15200253), anti-CTCF antibody (included in Diagenode #C01010055; control experiment)

Peak calling parameters

Trimmomatic version 0.39 was employed to trim reads after a quality drop below a mean of Q15 in a window of 5 nucleotides and keeping only filtered reads longer than 15 nucleotides. Reads were aligned versus Ensembl mouse genome version mm39 (Ensembl release 109) with STAR 2.7.11b. Alignments were filtered to remove: duplicates with Picard 3.1.1 (Picard: A set of tools (in Java) for working with next generation sequencing data in the BAM format), spliced, multi-mapping, ribosomal, or mitochondrial reads. Peak calling was performed with Macs version 3.0.0a7 with FDR < 0.001 and enrichment vs. input > 2x. Peaks overlapping ENCODE blacklisted regions (known misassemblies, satellite repeats) were excluded. Remaining peaks were unified to represent a common set of regions for all samples. Counts were produced with featureCounts. The raw count matrix was normalized with DESeq2 version 1.36.0 (Love et al., Moderated estimation of fold change and dispersion for RNA-Seq data with DESeq2). Peaks were annotated with the promoter (TSS +- 5000 nt) of the nearest gene based on Ensembl release 109.

Data quality

Quality control was performed based on peak calling using the following parameters: MACS3: mode: tf; filter: FDR <= 0.001, minimum treatment reads >= 1, enrichment >= 2, log10(qvalue) <= 0; not overlapping blacklisted regions like satellite repeats or misassemblies (ENCODE)
experiment, # peaks raw, # peaks filtered
Polr2a-WT_1, 32,916, 32,154
Polr2a-WT_2, 39,542, 38,680
Polr2a-ko-Mettl3_1, 34,236, 33,471
Polr2a-ko-Mettl3_2, 30,929, 30,217
Polr2a-ko-Trdn_1, 34,683, 33,884
Polr2a-ko-Trdn_2, 19,453, 18,917
IgG-WT_1, 30, 3
IgG-ko-Mettl3_1, 98, 48
IgG-ko-Trdn_1, 55, 11
Ctcf-ko-Mettl3_1, 77,853, 76,485
Ctcf-ko-Mettl3_2, 71,522, 70,244
Ctcf-ko-Trdn_1, 71,821, 70,475
Ctcf-ko-Trdn_2, 70,671, 69,373
Ctcf-WT_1, 71,611, 70,283
Ctcf-WT_2, 79,235, 77,834
union, , 101,256 (union/merge of peaks of all samples)

Software

See peak calling parameters description.
