# Supplementary material for: Trdn-as directs m6A-dependent transcriptional termination for accurate triadin isoform switching, preventing aberrant dyads and cardiomyopathy
Source: Nat Commun. 2026 Jul 25;17:7269. doi: 10.1038/s41467-026-75985-8 (PMC13401598; doi:10.1038/s41467-026-75985-8)
Supplement: Supplementary file 3 — Transparent Peer Review File [file 41467_2026_75985_MOESM3_ESM.pdf]

nature portfolio

Peer Review File

Trdn-as directs m6A-dependent transcriptional termination for accurate triadin isoform switching, preventing aberrant dyads and cardiomyopathy

Corresponding Author: Professor Thomas Boettger

This file contains all reviewer reports in order by version, followed by all author rebuttals in order by version.

Version 0:

Reviewer comments:

Reviewer #1

(Remarks to the Author)
Hofmann et al. present intriguing findings showing that reduced TRDN-AS expression leads to defective triadin isoform switching, resulting in increased skeletal muscle TRISK95 levels in cardiomyocytes and a series of cardiac abnormalities. The authors conducted a detailed analysis of the functional consequences of TRDN-AS deficiency using hiPSC-derived cardiomyocytes and mouse models, and established a signaling axis involving TEAD1/TRDN-AS/m^{6}A/TRISK95 that mediates these effects. They further explored the regulatory mechanisms of each layer within this axis, highlighting the crucial roles of TRDN-AS transcription in cis and METTL3-mediated RNA Pol II stalling in controlling the triadin isoform. However, to better assess the relevance of these findings, additional information regarding the significance of TRISK95 in the abnormal phenotypes associated with TRDN-AS deficiency should be provided, and the mechanistic analyses should be further strengthened.

1. The authors describe the functional consequences of TRDN-AS deficiency; however, it remains unclear whether these outcomes result specifically from the generation of TRISK95 and the subsequent alteration of the cardiac calcium release complex interactome. Would a selective reduction of TRISK32 produce similar effects? What happens to calcium release if TRISK32 is restored, or if TRISK95 is silenced in TRDN-AS-depleted hiPSC-derived cardiomyocytes? Addressing these points would help clarify the underlying mechanisms.
2. The authors propose that METTL3 is crucial for the formation of cardiac and skeletal muscle Trdn isoforms in a mouse model. Have METTL3 mRNA levels been analyzed using RNA-seq data from human heart failure patients? Additionally, was METTL3 protein expression examined in DCM samples with arrhythmias? Such analyses could provide valuable insight into the clinical significance of METTL3 in human cardiomyopathy.
3. The authors propose that TRDN-AS downregulation is at least partly due to reduced TEAD1 levels, supported by public ChIP-seq data showing TEAD1 binding at the TRDN-AS regulatory region. If the TEAD1 binding motif in the TRDN-AS reporter construct is mutated, does reporter expression decrease accordingly? Additionally, as both TRDN-AS and TEAD1 mRNA levels are decreased in human heart failure datasets, have the authors examined their correlation?
4. Figure 1D shows four patients with supraventricular tachycardia; however, the figure legend incorrectly indicates n = 5.
5. On page 8, “multiple ChIPseq data sets” should be corrected to “multiple ChIP-seq datasets.”

Reviewer #2

(Remarks to the Author)
Review:

It is well known that loss of triadin, as seen in patients with heart failure, leads to calcium leaks and catecholaminergic polymorphic ventricular tachycardia that can lead to ventricular fibrillation and sudden cardiac death. In this manuscript Hofmann et al. demonstrate that a reduction in the lncRNA TRDN-AS results in a switch of the cardiac isoform TRISK32 (cardiac) to TRISK95 (skeletal) in vitro and in vivo. Furthermore, they present evidence that TRISK95 causes altered dyad structure and calcium handling, resulting in arrhythmias and DCM. They show data from transgenic mice and iPSC-derived cardiomyocytes and some patient data supporting this. Mechanistically, they demonstrate METTL3-dependent TRDN

isoform switching and demonstrated a stalling of RNA Pol II and no RNA Pol II collision mechanism, as had previously been suggested.
This is an important and interesting topic, the results are well presented, and the paper is well written. However, to emphasize the new aspects, there are several major points that need to be addressed:

Major points:
1. A gain-of-function experiment should be performed. If the proposed new pathomechanism is true, overexpression of TRISK95 in CMs should induce CPVT in mice and spontaneous calcium release in iPSC-derived cardiomyocytes. This can be done in mice e.g. with an AAV9, carrying a codon optimized TRISK95 that is unaffected by TRDN-AS. In human iPSC-cardiomyocytes either a stable line could be generated or transduction with a TRISK95 expression construct in AAV6.

2. The authors convincingly demonstrate an influence of Mettl3 on isoform switching using the cardiomyocyte-specific Mettl3 knockout. However, an analysis of the phenotype is missing. Do the mice show the same phenotype as TRDN-AS KO mice, as would be expected?

3. The authors did not analyze other TRDN-isoforms, such as TRISK51 and Trisk49. They need to check whether they are expressed and play a role in cardiomyocytes. They should also mention them in the introduction and briefly in the discussion.

4. A knockout of Trdn-AS in mice has been published before (Zhao et al., 2022 Circulation). However, the authors did not fond TRISK95, neither in mice nor in failing hearts wherein cardiac triadin was significantly decreased. The authors should comment on this and discuss this contradiction to their data.

5. NT group size in Figure 1D is too small to produce meaningful statistics (NT=2; tachycardia SVT=5; ventr. Tachycardia n=3). Please increase the number for NT patients.

6. Are structural reconfigurations of the cardiac dyads also seen in patient hearts? Please provide EM pictures of human samples to see if the mechanism is conserved between species.

Reviewer #4

(Remarks to the Author)
I co-reviewed this manuscript with one of the reviewers who provided the listed reports. This is part of the Nature Communications initiative to facilitate training in peer review and to provide appropriate recognition for Early Career Researchers who co-review manuscripts.

Reviewer #5

(Remarks to the Author)
The MS describes a novel regulation by a lnc RNA in the triadin gene (lncRNA TRDN-AS) that causes a switch from cardiac to skeletal triadin in the heart. My major concerns relate to the biological consequences that supposedly are triggered by a reduction or loss of lncRNA TRDN-AS.

1. The biological consequences of ablation of the TRDN gene has been extensively studied in mice. I will focus on the heart phenotype, which is the subject of the current MS. Loss of cardiac triadin causes reduction in RYR2 protein, smaller junctional SR, slower Ca transient rise due to reduced EC coupling and slower Ca transient decay due to slower L-type Ca channel inactivation. The only difference is that in TRDN KO mice, both cardiac and skeletal triadin are lost, whereas in the TRDN-AS KO mice, there is an upregulation of skeletal triadin. Yet, the results reported here are complete opposite (faster Ca transient rise and decay, no change in RyR2, junctional SR remodeling not quantified, SR Ca content not quantified, heart failure phenotype (which is not present in TRDN KO mice). I find it difficult to understand that a modest upregulation of skeletal triadin in the heart is responsible for this dramatic and opposite phenotype. Maybe the way the TRDN AS KO mouse was generated had other unintended gene-regulatory consequences. At a minimum, the authors need to demonstrate what are the consequences of expressing skeletal triadin in the mouse heart, i.e. by AAV gene transfer.

2. The link to clinical data is a stretch. Everyone knows that is close to impossible to characterize patients with cardiomyopathy as having ventricular arrhythmias or not due to the stochastic nature of the phenotype, and sparse clinical phenotyping. Further confounding is that the authors lumped SVT and VT together, which have completely different etiology. Furthermore, using endocardial biopsies as tissue source is problematic, given the inherent variability of the tissue samples. Finally, the study is vastly underpowered. I would recommend removing the clinical data, or get a lot more samples from explanted hearts, not biopsies.

3. EM data need to quantified in blinded fashion. As presented, it is difficult to know what is real and what is selection bias.

4. The authors seem to lack expertise in electrophysiological and calcium handling phenotyping. For example, in Fig 3 J, the trace is a clear motion artifact, not VT. A common mistake by non-expert readers of murine telemetry records. Numerous other experimental concerns (lack of appropriate controls, no action potential or membrane current measurements, no

quantification of SR calcium content) make this reviewer concern about the validity of the presented functional data.

Version 1:

Reviewer comments:

Reviewer #1

(Remarks to the Author)
My concerns have been addressed, and I recommend publication.

Reviewer #2

(Remarks to the Author)
The manuscript has been significantly improved, and the hypothesis is now much more clearly structured and better supported. All major points of concern have been addressed. However, I have two more minor points/questions:

1. do the authors see PVTs in mice treated with AAV-TRISK95-T2A-mCherry?

2. do the EMs mentioned in point 6 refer to patients with altered TRDN expression, or to patients with general DCM/ICM where this has not been investigated?

Reviewer #5

(Remarks to the Author)
The authors have addressed most of my concerns, except the claims regarding arrhythmia.

The authors have no evidence for ventricular tachyarrhythmia, but only bradyarrhythmia and QT prolongation. Presenting a link to arrhythmia is not supported by the data reported, since QT prolongation, bradyarrhythmia and conduction blocks are a common bystander of dilated cardiomyopathy.

The primary phenotype is impaired EC coupling and dilated cardiomyopathy, not arrhythmia. It is ALSO NOT A primary long QT phenotype (by definition, ventricular structure and function is normal in genetic long QT). The human biopsy data also do NOT support a link between a primary arrhythmia phenotype and trdn-AS.

I strongly suggest refocusing the MS including title on cardiomyopathy, not arrhythmia.

Version 2:

Reviewer comments:

Reviewer #2

(Remarks to the Author)
All of my concerns have been addressed, and I appreciate the shift in focus toward cardiomyopathy and impaired calcium handling.

Reviewer #5

(Remarks to the Author)
The revised MS addressed my previous comments. No further concerns. Excellent work!

Open Access This Peer Review File is licensed under a Creative Commons Attribution 4.0 International License, which permits use, sharing, adaptation, distribution and reproduction in any medium or format, as long as you give appropriate credit to the original author(s) and the source, provide a link to the Creative Commons license, and indicate if changes were made.
In cases where reviewers are anonymous, credit should be given to 'Anonymous Referee' and the source.
The images or other third party material in this Peer Review File are included in the article's Creative Commons license, unless indicated otherwise in a credit line to the material. If material is not included in the article's Creative Commons license and your intended use is not permitted by statutory regulation or exceeds the permitted use, you will need to obtain permission directly from the copyright holder.
To view a copy of this license, visit https://creativecommons.org/licenses/by/4.0/

RESPONSES TO REVIEWERS' COMMENTS

Reviewer #1 (Remarks to the Author):

Hofmann et al. present intriguing findings showing that reduced TRDN-AS expression leads to defective triadin isoform switching, resulting in increased skeletal muscle TRISK95 levels in cardiomyocytes and a series of cardiac abnormalities. The authors conducted a detailed analysis of the functional consequences of TRDN-AS deficiency using hiPSC-derived cardiomyocytes and mouse models, and established a signaling axis involving TEAD1/TRDN-AS/m^{6}A/TRISK95 that mediates these effects. They further explored the regulatory mechanisms of each layer within this axis, highlighting the crucial roles of TRDN-AS transcription in cis and METTL3-mediated RNA Pol II stalling in controlling the triadin isoform. However, to better assess the relevance of these findings, additional information regarding the significance of TRISK95 in the abnormal phenotypes associated with TRDN-AS deficiency should be provided, and the mechanistic analyses should be further strengthened.

Response: We thank the reviewer for the insightful evaluation of our manuscript.

1. The authors describe the functional consequences of TRDN-AS deficiency; however, it remains unclear whether these outcomes result specifically from the generation of TRISK95 and the subsequent alteration of the cardiac calcium release complex interactome. Would a selective reduction of TRISK32 produce similar effects? What happens to calcium release if TRISK32 is restored, or if TRISK95 is silenced in TRDN-AS-depleted hiPSC-derived cardiomyocytes? Addressing these points would help clarify the underlying mechanisms.

Response: The isolated effect of loss of the cardiac TRDN on cardiomyocyte physiology has been described by the group of B. Knollmann in the seminal study (Chopra N, et al., Proc Natl Acad Sci U S A. 2009;106(18):7636-41.). This study describes a phenotype that is in many aspects opposite to the phenotype we observed after termination of *Trdn-as* expression and the subsequent TRDN isoform switch.

After deletion of cardiac TRDN the general structure of the dyads is maintained, although alterations of several parameters including jSR contact length or changed jSR width and more importantly slower calcium transients were observed in isolated cardiomyocytes. Deletion of cardiac triadin reduces expression of RYR2, CASC-2, JP-1/2 and almost abrogates expression of Junctin. In contrast, the switch of the TRDN isoforms causes fundamentally different effects, showing increased expression of RYR2 and CASC2, while expression of Junctin and JP2 was not significantly altered.

Deletion of *Trdn-as* results in an almost complete replacement of cardiac Triadin (TRISK32) with the larger isoform TRISK95 (and TRISK51, new Supplementary Fig. S2e). These larger isoforms contain an additional C-terminal protein domain that is assumed to extend into the lumen of the jSR. The additional C-terminal part of the protein contains amino acids and motifs that are essential for protein-protein interactions, in particular together with the cytoskeleton-associated CLIMP-63/CKAP4. Presence of this C-terminal part results in deformation of the ER in cultured cells (Fourest-Lieuvin, A et al. J Cell Sci. 2012;125(14):3443-53; Osseni, A et al. J Cell Sci. 2016;129(20):3744-55). The IP-MS analysis in *Trdn-as*-deficient cardiomyocytes that had undergone the TRDN isoform switch revealed interaction of TRDN with CKAP4/5 and with multiple components of the cytoskeleton. These molecular changes are in line with the previous observation of ER-deformation upon TRISK95 expression, which results in massive deformation of the dyad structure with a reduced junctional cleft, implying that these changes are primarily related to the expression of the TRISK95 isoform of TRDN, rather than to the loss of TRISK32. In contrast to TRDN-deletion in cardiomyocytes, the TRDN isoform switch also results in faster calcium transients in isolated cardiomyocytes and spontaneous calcium release without stimulation of cardiomyocytes.

The massive differences in phenotype comparing complete TRDN-deletion and TRDN isoform switching are not surprising. Even after complete isoform switching, the TRISK95 isoforms still contains the almost complete TRISK32 protein, but with a C-terminal extension. Thus, the isoform switch does

not result in a loss of function for TRISK32, but in additional properties, enabling interactions with the cytoskeleton, causing deformation of the dyad structure and subsequent aberrant calcium release. Deletion of TRISK95 after isoform switching also removes the core TRISK32 component of TRDN essentially resulting in the previously described TRDN loss of function phenotype.

To mimic the situation of TRISK95 expression in cardiomyocytes with additional presence of TRISK32 protein and to address the contribution of TRISK95 to the *Trdn-as* induced phenotype, we expressed TRISK95 in WT cardiomyocytes using AAVs. We observed increased numbers of spontaneous calcium transients (new Supplementary Fig. S3), very similar to the condition after inactivation of *Trdn-as*. We also analyzed the TRDN-interactome after cardiomyocyte-specific deletion of Mettl3, which recapitulated the changes in the TRDN interactome seen in *Trdn-as* mutant cells, although the triadin isoform switch was less pronounced than in *Trdn-as* mutant cardiomyocytes.

2. The authors propose that METTL3 is crucial for the formation of cardiac and skeletal muscle Trdn isoforms in a mouse model. Have METTL3 mRNA levels been analyzed using RNA-seq data from human heart failure patients? Additionally, was METTL3 protein expression examined in DCM samples with arrhythmias? Such analyses could provide valuable insight into the clinical significance of METTL3 in human cardiomyopathy.

Response: We now include data on *METTL3* expression in human hearts based on the previously analysed GSE116250 RNA-seq data. No significant reduction of *METTL3* RNA was detected using this data (new Supplementary Fig. S6d). We also performed Western blot analysis for METTL3 using human heart samples derived from DCM or DCM patients with additional arrhythmia. Again, we detected no significant difference in METTL3 expression (new Supplementary Fig. S6e, f). We would like to point out that deletion of *Mettl3* in cardiomyocytes induces only a moderate switch of TRDN isoforms when compared to deletion of *Trdn-as*. We now include new data, demonstrating that even this moderate isoform switch is sufficient to change the interactome of TRDN. We observed stronger, most likely CKAP-mediated, interactions with the cytoskeleton (new Supplementary Fig. S6a). We reason that the less pronounced triadin isoform switch is not sufficient to significantly change calcium release properties of isolated cardiomyocytes (not shown). In addition, analysis of RNA-seq data from Mettl3-deficent hearts revealed multiple additional transcriptional changes that have the potential to modulate the phenotype caused by the TRDN isoform switch.

We demonstrate that transcriptional interference as well as enhanced proximal polyadenylation by m6A modifications are important for the isoform switch of TRISK95 to TRISK32 in cardiomyocytes. Transcription of *Trdn-as* is absolutely required, whereas m6A modifications seem to have less pronounced effects. We conclude that modulation of TRDN-isoform expression by METTL3 most likely has very limited clinical significance in human cardiomyopathy, which is in line with the absence of detectable changes in METTL3 expression in DCM or DCM patients with additional arrhythmia. METTL3 may have other effects on the heart by deregulating polyA site selection at other transcipts but this was not the focus of our study. We assume that the switch of TRIADIN-isoform expression in human DCM patients is primarily driven by the modulation of *TRDN-AS* transcription, e.g. by the reduced binding of the transcription factor TEAD1, which is also supported by analysis of available ChIP-seq data. We have modified the discussion of our work accordingly.

3. The authors propose that TRDN-AS downregulation is at least partly due to reduced TEAD1 levels, supported by public ChIP-seq data showing TEAD1 binding at the TRDN-AS regulatory region. If the TEAD1 binding motif in the TRDN-AS reporter construct is mutated, does reporter expression decrease accordingly? Additionally, as both TRDN-AS and TEAD1 mRNA levels are decreased in human heart failure datasets, have the authors examined their correlation?

Response: We thank the reviewer for these excellent suggestions. We now provide new data confirming the importance of the TEAD1 binding motifs at the *Trdn-as* promoter and enhancer (new

Fig. 6i). In addition, we detected a significant correlation between expression of TEAD1 and *TRDN-AS* using published RNA-seq data for NF and ICM patients (new Supplementary Fig. S5e). However, we did not find a significant correlation for DCM patients. These new data are now included in the manuscript.

4. Figure 1D shows four patients with supraventricular tachycardia; however, the figure legend incorrectly indicates n = 5.

Response: We are sorry for this mistake, which was corrected. We now only compare DCM patients to DCM patients with ventricular arrhythmia and added additional samples to the analysis (Fig. 1d, new Supplementary Fig. S1).

5. On page 8, "multiple ChIPseq data sets" should be corrected to "multiple ChIP-seq datasets."

Response: We revised the spelling throughout the manuscript.

Reviewer #2 (Remarks to the Author):

It is well known that loss of triadin, as seen in patients with heart failure, leads to calcium leaks and catecholaminergic polymorphic ventricular tachycardia that can lead to ventricular fibrillation and sudden cardiac death. In this manuscript Hofmann et al. demonstrate that a reduction in the lncRNA TRDN-AS results in a switch of the cardiac isoform TRISK32 (cardiac) to TRISK95 (skeletal) in vitro and in vivo. Furthermore, they present evidence that TRISK95 causes altered dyad structure and calcium handling, resulting in arrhythmias and DCM. They show data from transgenic mice and iPSC-derived cardiomyocytes and some patient data supporting this. Mechanistically, they demonstrate METTL3-dependent TRDN isoform switching and demonstrated a stalling of RNA Pol II and no RNA Pol II collision mechanism, as had previously been suggested.

This is an important and interesting topic, the results are well presented, and the paper is well written. However, to emphasize the new aspects, there are several major points that need to be addressed:

Major points:

1. A gain-of-function experiment should be performed. If the proposed new pathomechanism is true, overexpression of TRISK95 in CMs should induce CPVT in mice and spontaneous calcium release in iPSC-derived cardiomyocytes. This can be done in mice e.g. with an AAV9, carrying a codon optimized TRISK95 that is unaffected by TRDN-AS. In human iPSC-cardiomyocytes either a stable line could be generated or transduction with a TRISK95 expression construct in AAV6.

Response: The reviewer raised an important point. To analyze the impact of TRISK95 on heart function after loss of *Trdn-as* transcription, we performed gain of function experiments by expressing TRISK95 in WT hearts using AAVs. We injected AAV-mCherry or AAV-TRISK95-T2A-mCherry into WT mice via the tail vein. Cardiomyocytes were isolated and calcium release of cardiomyocytes were analyzed using the IonOptix system. We observed increased numbers of spontaneous calcium release (SCR) in TRISK95-expressing cardiomyocytes as well as increased numbers of spontaneous calcium oscillations (new Supplementary Fig. S3). These data indicate that the presence of skeletal muscle TRISK95 is sufficient to modulate important characteristics of the cardiomyocyte calcium release, despite expression of TRISK32.

We would also like to stress that the luminal domains of TRISK95, which are not present in TRISK32, are essential for CKAP4-mediated interactions with the cytoskeleton. The CKAP4-mediated interactions with the cytoskeleton are responsible for deformation of the ER in cultured cells (Fourest-Lieuvin, A et al. J Cell Sci. 2012;125(14):3443-53; Osseni, A et al. J Cell Sci. 2016;129(20):3744-55), which directly corresponds to the deformation of dyad structures in cardiomyocytes after loss of *Trdn-as* transcription and the TRISK32 to TRISK95 switch.

Taken together the data demonstrate that the phenotype described after termination of *Trdn-as* transcription is caused by the switch of TRDN isoforms, which adds the C-terminal TRDN domain that mediates interaction with CKAPs and the cytoskeleton. Our results unequivocally show that expression of skeletal muscle TRISK95 is sufficient for induction of spontaneous calcium transients in cardiomyocytes.

2. The authors convincingly demonstrate an influence of Mettl3 on isoform switching using the cardiomyocyte-specific Mettl3 knockout. However, an analysis of the phenotype is missing. Do the mice show the same phenotype as TRDN-AS KO mice, as would be expected?

Response: The comment relates to comment #2 of reviewer #1. We previously demonstrated that METTL3 is involved in controlling selection of proximal polyA sites, thereby affecting expression of TRDN-isoforms. We followed the reviewer’s advice and performed a closer analysis of the cardiomyocyte-specific *Mettl3* mutants. We now demonstrate that inactivation of *Mettl3* changes interaction of proteins with TRDN, partially mirroring observations after termination of *Trdn-as* transcription. In particular we demonstrate increased interaction of TRDN with CLIMP-63/CKAP4, in

line with increased interaction of TRDN with proteins of the cytoskeleton as demonstrated for the *Trdn-as* KO (new Supplementary Fig. S6a). However, we did not observe faster calcium release after termination of *Trdn-as* transcription.

We would like to emphasize that transcriptional interference as well as promotion of proximal polyadenylation of the TRDN transcript contribute to the switch of TRDN isoforms. However, the impact of transcriptional interference mediated by *Trdn-as* is much stronger compared to m6A modifications. Deletion of *Mettl3* only causes a moderate increase of TRISK95, whereas termination of *Trdn-as* transcription causes a nearly complete switch from TRISK32 to TRISK95 (compare Fig. 2i vs. Fig. 7l). The difference in the efficiency of triadin isoform switching explains the lower physiological impact of *Mettl3* deletion compared to termination of *Trdn-as* transcription. Nevertheless, previous studies reported a late onset (8 months) DCM phenotype in *Mettl3*-mutants, which resembles findings in the *Trdn-as* mutants (Dorn et al. Circulation 139.4 (2019): 533-545). Our analysis was done 2-3 weeks after deletion of *Mettl3* so that we did not observe these changes.

Deletion of *Mettl3* induces a complex phenotype in cardiomyocytes with numerous transcriptional changes. RNA-seq data analysis revealed massive changes in the transcriptome of *Mettl3* mutants, which are much stronger and do not overlap with the changes induced after termination of *Trdn-as* transcription (Figure 1 for reviewer). Termination of *Trdn-as* essentially affected only the *Trdn*-locus, with little additional effects at the transcript level, whereas deletion of *Mettl3* deregulated multiple transcripts. Thorough understanding of the molecular function of METTL3 and its pathophysiological functions will require additional dedicated studies. We reason that the additional effects of *Mettl3* deletion may alter the phenotype induced by loss of proximal *Trdn* polyadenylation.

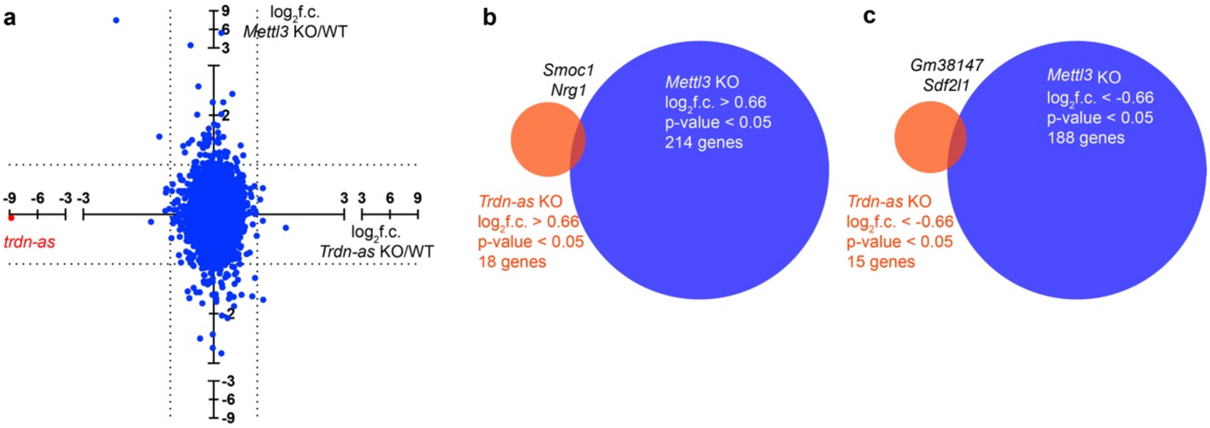

Figure 1 for reviewer: RNA-Seq. data of hearts of *Trdn-as* KO and *Mettl3* KO mice compared to controls.

a Genes detected with average TPKM > 10 were plotted with the respective log_2fold change *Trdn-as* KO / WT (X-axis) and *Mettl3* KO / WT (Y-axis). Dotted lines indicate a log_2f.c. > 1 or < -1, respectively. At this level, there is no overlap of genes similarly deregulated in both models.

b, c Venn diagrams using significantly up- (**b**) or downregulated (**c**) genes with a log_2f.c. > 0.66 or < -0.66 after deletion of *Trdn-as* transcription or deletion of METTL3, respectively.

3. The authors did not analyze other TRDN-isoforms, such as TRISK51 and Trisk49. They need to check whether they are expressed and play a role in cardiomyocytes. They should also mention them in the introduction and briefly in the discussion.

Response: We thank the reviewer for this suggestion. We now include an additional western blot demonstrating that termination of *Trdn-as* transcription in the heart also enables expression of TRISK51 (new Supplementary Fig. S2e). Previous studies have addressed the function of different

skeletal muscle specific TRDN-isoforms in detail, revealing that TRISK95 and TRISK51 share several features. The function of the luminal domains of TRISK95 and TRISK51 in the organization of the reticulum membrane are mediated by conserved cysteine residues and by a coiled-coil domain, containing KEKE motifs (Fourest-Lieuvin, A et al. J Cell Sci. 2012;125(14):3443-53; Osseni, A et al. J Cell Sci. 2016;129(20):3744-55.; Marty I, 2016, J Physiol.). TRISK51 lacks one of the cysteines present in TRISK95, but the other cysteine and the coiled-coil domain are present in both TRISK95 and TRISK51. The coiled-coil domain is the important motif mediating interaction with CLIMP-63/CKAP4 that is essential for linking triads and microtubules (Osseni et al., 2016). These studies suggest that major functions of skeletal muscle TRDN are shared between these two TRDN isoforms also in respect to ER deformation in cultured cells.

TRISK49 so far has only been identified in rat skeletal muscle and it is unclear whether such an isoform exists in mouse or human skeletal muscle. Analysis of RNA-seq data revealed intron retention after exon 21 of TRDN in *Trdn-as* KO heart and TA muscle, which allows generation of the TRISK51-encoding transcript, in line with the presence of TRISK51 protein, detected by the additional western blot. In contrast, splicing seems occurred efficiently at the junction of exon 20 – 21 (the site where TRISK49 would be encoded after intron retention), indicating there is no significant translation of TRISK49-encoding transcripts in the mouse.

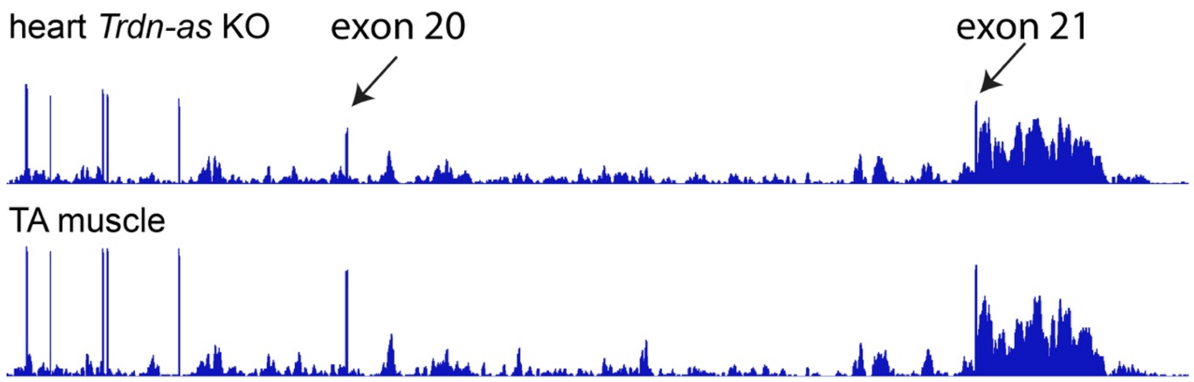

Figure 2 for reviewer: Intron retention results in alternative formation of TRDN C-termini.

TRISK51 is encoded by transcripts produced by intron retention following exon 21 of *Trdn*, whereas TRISK49 in principle, based on amino acid sequences, would be encoded by intron retention after exon 20. So far TRISK49 was only found in skeletal muscles of rats. RNA-seq data for mouse heart after deletion of *Trdn-as* transcription or in TA muscle indicate intron retention after exon 21, but not between exon 20 to exon 21, preventing generation of TRISK49.

4. A knockout of Trdn-AS in mice has been published before (Zhao et al., 2022 Circulation). However, the authors did not fond TRISK95, neither in mice nor in failing hearts wherein cardiac triadin was significantly decreased. The authors should comment on this and discuss this contradiction to their data.

Response: We thank the reviewer for the opportunity to describe differences between the model of Zhao et al. and our model. In contrast to our model, Zhao et al. deleted exon 2 and exon 3 of *Trdn-as* and observed a reduction of the expression of TRISK32 but no TRISK95 expression. Functionally, the approach by Zhao et al generates a partial TRDN loss-of-function model, similar to the previously described triadin knockout models. It does not allow an isoform switch or gain-of-function scenario.

Our data reveal that *Trdn-as* expression extends well beyond the previously annotated *Trdn-as* transcript. We show that the antisense transcription results in transcriptional interference and enables

m6A-mediated selection of a proximal polyA site at exon 9 of *Trdn*, resulting in polyadenylation of the sense *Trdn* transcript. Deletion of exons 2/3 of the annotated *Trdn-as* transcript in the Zhao et al. model does not terminate transcription of the *Trdn-as* locus. Thus, it does not prevent transcriptional interference at exon 8/9 and consequently does not result in TRISK95 expression. Comparison to the model of Zhao et al. supports our concept that transcriptional interference, together with m6A modification of the transcipts, is essential for proximal termination and polyadenylation of *Trdn* and TRISK32 expression. We now discuss the differences of the models in more detail in the revised manuscript.

5. NT group size in Figure 1D is too small to produce meaningful statistics (NT=2; tachycardia SVT=5; ventr. Tachycardia n=3). Please increase the number for NT patients.

Response: The reviewer is right. We previously had only limited access to human samples and therefore could only provide proof-of-principle findings, showing that TRISK95 can be expressed in diseased human heart. We now have included a larger group of human samples from patients with DCM, which allows a proper statistical analysis. Analysis of the TRISK95 / TRISK32 ratio revealed that patients with ventricular arrhythmia (revised Fig. 1d) or proven record of ECG abnormalities (new Supplementary Fig. S1) are characterized by an increased TRISK95 / TRISK32 ratio.

6. Are structural reconfigurations of the cardiac dyads also seen in patient hearts? Please provide EM pictures of human samples to see if the mechanism is conserved between species.

Response: Heart samples from DCM and ICM patients or from animal models already have been analyzed in great detail by electron microscopy in the past (Hong & Shaw, 2017, Physiol Rev 97: 227). Different groups demonstrated that heart failure often results in massive restructuring and dilation of T-tubules, jSR dilation and fragmentation, reduced coupling of the dyads, and increased dyad cleft width or unzipping of the dyad structures. Several molecules were identified to potentially participate in these processes including JPH2 (Zhang HB, et al., Cardiovasc Res. 2013;98(2):269-76) and BIN1 (Hong TT, et al., Heart Rhythm. 2012;9(5):812-20). The studies illustrate that regulation of the dyad structure in human cardiomyopathy is a complex and dynamic process, probably depending on several causes. Such a situation is rather different from a monocausal genetic model. Since dyads have already been investigated in human DCM and ICM patients by EM and the availability of EM-quality samples from human hearts is limited, we would like to refer to the published examples and not pursue additional EM studies in humans.

Reviewer #4 (Remarks to the Author):

I co-reviewed this manuscript with one of the reviewers who provided the listed reports. This is part of the Nature Communications initiative to facilitate training in peer review and to provide appropriate recognition for Early Career Researchers who co-review manuscripts.

Response: We thank this reviewer for co-reviewing the manuscript.

Reviewer #5 (Remarks to the Author):

The MS describes a novel regulation by a lnc RNA in the triadin gene (lncRNA TRDN-AS) that causes a switch from cardiac to skeletal triadin in the heart. My major concerns relate to the biological consequences that supposedly are triggered by a reduction or loss of lncRNA TRDN-AS.

1. The biological consequences of ablation of the TRDN gene has been extensively studied in mice. I will focus on the heart phenotype, which is the subject of the current MS. Loss of cardiac triadin causes reduction in RYR2 protein, smaller junctional SR, slower Ca transient rise due to reduced EC coupling and slower Ca transient decay due to slower L-type Ca channel inactivation. The only difference is that in TRDN KO mice, both cardiac and skeletal triadin are lost, whereas in the TRDN-AS KO mice, there is an upregulation of skeletal triadin. Yet, the results reported here are complete opposite (faster Ca transient rise and decay, no change in RyR2, junctional SR remodeling not quantified, SR Ca content not quantified, heart failure phenotype (which is not present in TRDN KO mice). I find it difficult to understand that a modest upregulation of skeletal triadin in the heart is responsible for this dramatic and opposite phenotype. Maybe the way the TRDN AS KO mouse was generated had other unintended gene-regulatory consequences. At a minimum, the authors need to demonstrate what are the consequences of expressing skeletal triadin in the mouse heart, i.e. by AAV gene transfer.

Response: We thank the reviewer for the opportunity to elaborate on the differences between *Trdn-KO* and *Trdn-as* KO mouse model in more detail. The only isoform of TRDN expressed in the healthy heart is TRISK32. Deletion of *Trdn* results in the phenotype described by the reviewer. In contrast, termination of *Trdn-as* transcription results in extended transcription of the sense *Trdn* locus, generating a longer transcript encoding the skeletal muscle specific isoform of *Trdn*, TRISK95. TRISK95 contains most of the cardiac specific TRDN protein and additional luminal protein domains, which are normally only present in skeletal muscle. The substantial (not modest) presence of TRISK95 instead of TRISK32 explains why the phenotype of *Trdn-as* KO differs substantially from *Trdn-KO* mice. TRISK51/95 contains additional luminal parts, extending into the SR lumen, which are not included in TRISK32. The luminal parts mediate interactions with cytoskeletal proteins via CLIMP-63 / CKAP4 and deformed the ER in cultured cells (Fourest-Lieuvin, A et al. J Cell Sci. 2012;125(14):3443-53; Osseni, A et al. J Cell Sci. 2016;129(20):3744-55). These features correspond exactly to the molecular changes observed in the TRDN-IP experiments: we observed interactions with CKAPs and components of the cardiomyocytes cytoskeleton due to formation of TRISK95, following termination of *Trdn-as*. We also observed deformation of dyads, in line with the deformation of the ER observed upon expression of TRISK95. Moreover, concentrations of RYR2, JPH-2, CASC-2 and Junctin are stable in *Trdn-as* KO mice but severely reduced in *Trdn-KO* hearts.

We followed the reviewer's advice and analyzed whether the presence of TRISK95, containing the luminal domains of TRDN, is sufficient to induced pathological calcium handling seen in *Trdn-as* KO. We expressed TRISK95 in WT cardiomyocytes using AAVs, uncovering that the presence of TRISK95 induces spontaneous calcium transients and calcium oscillations (new Supplementary Fig. S3). We reason that these new findings confirm the pathophysiological role of TRISK95 in cardiomyocytes.

The reviewer is right that genetic manipulation of mice might result in unintended gene-regulatory consequences. However, additional transcriptome analysis of *Trdn-as* KO hearts did not reveal major transcriptional changes. We only observed a relatively low number of significantly deregulated genes. Thus, we are confident that the *Trdn-as* KO phenotype is primarily caused by the TRDN isoform switch. Moreover, the *Trdn-as* KO phenotype was fully recapitulated in human hiPSC-derived cardiomyocytes, in which termination of *Trdn-as* transcription was accomplished by a different approach.

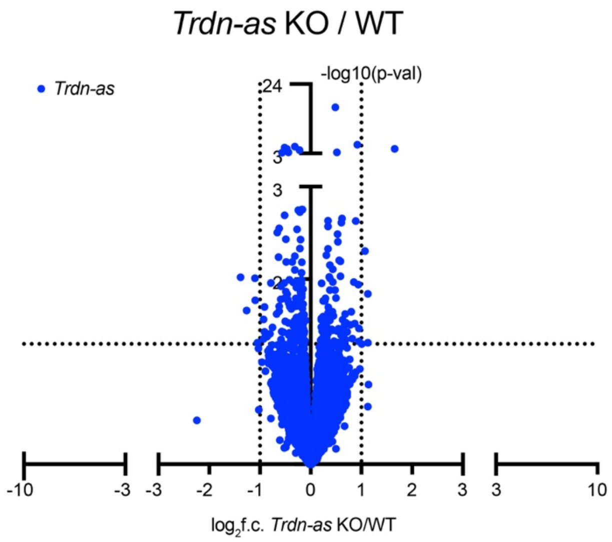

Figure 3 for reviewer: Volcano blot of *Trdn-as* KO vs WT heart transcriptome.

While we observe a massive downregulation of the *Trdn-as* transcript, only very little additional significant changes were detected in the *Trdn-as* transcriptome (10 transcripts are $\log_2$(f.c.) > 1 / < -1 and $-\log_{10}$(p-value) > 1.3; n = 3 KO / 3 WT hearts, average detection > 10 TPKM).

2. The link to clinical data is a stretch. Everyone knows that is close to impossible to characterize patients with cardiomyopathy as having ventricular arrhythmias or not due to the stochastic nature of the phenotype, and sparse clinical phenotyping. Further confounding is that the authors lumped SVT and VT together, which have completely different etiology. Furthermore, using endocardial biopsies as tissue source is problematic, given the inherent variability of the tissue samples. Finally, the study is vastly underpowered. I would recommend removing the clinical data, or get a lot more samples from explanted hearts, not biopsies.

Response: We apologise for the confusion regarding SVT and VT samples. We are well aware of the differences in the etiology, but due to the limited access to human samples, we simply wanted to make the point that TRISK95 can indeed be present in the diseased human heart.

Following the request of the reviewer, we enlarged the number of human myocardial biopsy samples from DCM patients. We observed presence of TRISK95 in the majority of samples from patients with DCM. Patients with ventricular arrhythmia (revised Fig. 2d) or proven record of ECG abnormalities (new Supplementary Fig. 1) showed an increased ratio of TRISK95 / TRISK32 compared to DCM patients without electrophysiological abnormalities.

One of our co-authors is a cardiologist specialized in rhythmology. Thus, we are well aware that arrhythmia is difficult excluded for patients with cardiomyopathy. It is possible that DCM patients in the cohort without documented electrophysiological symptoms may have undiagnosed arrhythmia. Removal of these patients most likely would increase significance of these results. However, we can only rely on diagnosed symptoms, which we did. We would also like to stress the striking parallels between the mouse and human model systems and in the clinical findings in human biopsies.

3. EM data need to quantified in blinded fashion. As presented, it is difficult to know what is real and what is selection bias.

Response: Thank you for this comment. We have now re-analyzed all EM data in a blinded fashion by two independent investigators. The analysis revealed a high proportion (approx. 80 %) of dyads with a collapse of the junctional cleft / dyad structure in *Trdn-as* KO hearts. Since collapse of the junctional

cleft prevents quantification of the width of the junctional cleft, such a quantification was only done for the dyads without collapse. Junctional clefts and jSR widths were quantified and compared to WT hearts. Quantification revealed a highly significant reduction of junctional cleft width and increased jSR width. The new data are included in Supplementary Fig. S4e-h.

4. The authors seem to lack expertise in electrophysiological and calcium handling phenotyping. For example, in Fig 3 J, the trace is a clear motion artifact, not VT. A common mistake by non-expert readers of murine telemetry records. Numerous other experimental concerns (lack of appropriate controls, no action potential or membrane current measurements, no quantification of SR calcium content) make this reviewer concern about the validity of the presented functional data.

Response: We respectfully disagree with the reviewer's opinion. ECG and calcium-contractility measurements were independently evaluated by investigators with long-standing expertise in cardiac electrophysiology and calcium handling, including Dr. Isabel Marty (accomplished expert in calcium signaling and excitation–contraction coupling with several papers about TRDN), Dr. Christian Wächter (senior cardiologist with extensive clinical and experimental experience in electrophysiology), and Dr. Hendrik Milting (expert in translational cardiac research and human cardiac phenotyping). We have routinely performed murine ECG analyses for many years, using established classifications (see for example Besser J, et al., PLoS One. 2014 Nov 21;9(11):e113449). We are well aware of motion artifacts that occur frequently, even when using implanted ECG-transmitters. We classified VTs based on the extended lag of the ECG signal following the disturbed signal, which never happened in control animals. To avoid any confusion, we removed Figure 3j and reassessed the ECG-data and quantified bradycardial episodes.

We would also like to emphasize that we did not want to deliver a comprehensive electrophysiological assessment of *Trdn-as* mice but primarily wanted to understand the consequences of the lack of *Trdn-as*, which resulted in increased levels of TRISK95, change of the dyad configuration, altered protein-protein interactions of TRDN and structural changes. In this context, we do not see any immediate benefits from measurement of action potentials, membrane currents or quantification of SR calcium content.

Responses to reviewers' comments

Reviewer #1 (Remarks to the Author):

My concerns have been addressed, and I recommend publication.

We thank the reviewer for his insightful comments that helped to improve the manuscript.

Reviewer #2 (Remarks to the Author):

The manuscript has been significantly improved, and the hypothesis is now much more clearly structured and better supported. All major points of concern have been addressed. However, I have two more minor points/questions:

1. do the authors see PVTs in mice treated with AAV-TRISK95-T2A-mCherry?

Response: We did not observe PVTs in mice treated with AAV-TRISK95-T2A-mCherry. We demonstrate that ectopic expression of TRISK95 induces extra spontaneous calcium transients as well as self-ending calcium oscillations, confirming our model. The absence of PVTs after isolated expression of TRISK95 may have different reasons, including expression level of TRISK95, normal expression of TRISK32, which is reduced in Trdn-as mutant hearts, or others.

2. do the EMs mentioned in point 6 refer to patients with altered TRDN expression, or to patients with general DCM/ICM where this has not been investigated?

Response: The EMs discussed in point 6 do not specifically refer to patients with altered TRDN expression, but to patients with DCM/ICM. Alterations in TRDN isoform were not analysed in the context of the EM studies, since it was not known before that changes in TRDN isoforms are pathophysiologically relevant. We are currently attempting to gather human samples for a comprehensive analysis that also includes ultrastructural examinations to corroborate the link between alterations in the dyad structure and increased TRISK95 expression. However, such samples are not easy to come and it will take substantial efforts to complete a thorough analysis, which should also address numerous other parameters.

Reviewer #5 (Remarks to the Author):

The authors have addressed most of my concerns, except the claims regarding arrhythmia.

The authors have no evidence for ventricular tachyarrhythmia, but only bradyarrhythmia and QT prolongation. Presenting a link to arrhythmia is not supported by the data reported, since QT prolongation, bradyarrhythmia and conduction blocks are a common bystander of dilated cardiomyopathy.

Response: We agree with the reviewer that we observe bradyarrhythmia but not ventricular tachyarrhythmia. Bradyarrhythmia clearly is also a form of arrhythmia, but we do not insist to mention this term in the title, since it may lead readers on the wrong track. We now write about 'aberrant Ca2+ handling', 'aberrant dyad structure' or 'bradyarrhythmia and QT prolongation' to be more precise, avoiding a general reference to arrhythmia.

The primary phenotype is impaired EC coupling and dilated cardiomyopathy, not arrhythmia. It is ALSO NOT A primary long QT phenotype (by definition, ventricular structure and function is normal in genetic long QT). The human biopsy data also do NOT support a link between a primary arrhythmia phenotype and trdn-AS.

I strongly suggest refocusing the MS including title on cardiomyopathy, not arrhythmia.

Response: We agree about the reviewer's statement regarding impaired EC coupling, which causes QT prolongation. Impaired EC coupling in the heart is recognized as a key component in the pathophysiology of Long QT Syndrome (https://doi.org/10.1093/eurheartj/ehac135). We do not think that the electrophysiological abnormalities are solely the consequence of dilated cardiomyopathy in Trdn-as mutant hearts, because of a simple striking argument. QT prolongation, bradyarrhythmia, and conduction block occur in Trdn-as mutant hearts well before any signs of dilated cardiomyopathy. We detected the electrophysiological abnormalities in 20-weeks-old Trdn-as mutants, which at this stage do not show any morphological signs of dilated cardiomyopathy. Symptoms of dilated cardiomyopathy only become apparent in 40-weeks-old Trdn-as mutants. To emphasize this point, we have included a Figure for the Reviewer, which demonstrates no significant change in cross sectional area of cardiomyocytes, i.e. no cardiomyopathy in 20-weeks-old Trdn-as mutants. Unfortunately, we did not point out this finding in the previous version of the manuscript. From the succession of events, we conclude that QT prolongation and bradyarrhythmia are not mere consequences of dilated cardiomyopathy but the result of impaired EC coupling due to aberrant dyad structures, caused by the TRDN isoform switch. We discuss these findings in the updated version of the manuscript and also make clear that impaired E/C coupling occurs well before any signs of dilated cardiomyopathy.

As mentioned above, we have changed the wording of the manuscript, including title and abstract, avoiding a general reference to arrhythmia, instead featuring cardiomyopathy and impaired EC coupling.

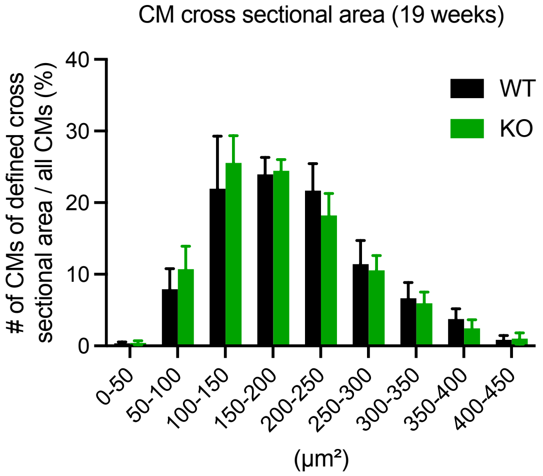

Figure for reviewer: Quantitative assessment of cross-sectional areas of cardiomyocyte at 19 weeks of age (n = 5 WT / 5 *Trdn-as* KO hearts). Cross-sectional areas were determined using WGA-stained heart sections.
